# Ruthenium Complexes of Atovaquone Acting on Multiple Stages of the *Plasmodium* Life Cycle

**DOI:** 10.1021/acs.jmedchem.5c02978

**Published:** 2026-04-11

**Authors:** Camila Fabbri, Pedro Henrique S. Marcon, Aline de Sousa Santiago, Caroline Conceição Sousa, Helenita Costa Quadros, Larissa de Sena Lamar Nunes, Dione D. Maciel de Menezes, Rosa Amélia Gonçalves Santana, Silvia Cássia B. Justiniano, Sarah D’Alessandro, Nicoletta Basilico, Diogo R. M. Moreira, João Honorato de Araujo-Neto, Stefanie Costa Pinto Lopes

**Affiliations:** † Fundação de Medicina Tropical Dr. Heitor Vieira Dourado (FT-HVD), Unidade de Pesquisa Clínica Carlos Borborema, 69040-000 Manaus, AM, Brazil; ‡ Instituto Leônidas e Maria Deane. Fiocruz Amazônia, 69057-070 Manaus, AM, Brazil; § Departamento de Química Fundamental. Instituto de Química, Universidade de São Paulo (USP). São Paulo, 05508-000 São Paulo, SP, Brazil; ∥ Instituto Gonçalo Moniz. Fiocruz Bahia, 40296-710 Salvador, BA, Brazil; ⊥ Dipartimento di Scienze Farmacologiche e Biomolecolari, Università degli Studi di Milano, 20133 Milan, Italy; # Dipartimento di Scienze Biomediche, Chirurgiche e Odontoiatriche, Universitá degli Studi di Milano, 20133 Milan, Italy

## Abstract

Here, we present the synthesis, characterization, and pharmacological evaluation of ruthenium complexes of broad-spectrum drug Atovaquone (ATV). Structure–activity relationships revealed key determinants for antiplasmodial activity, such as the importance of the oxidation state [Ru­(III) *versus* Ru­(II)] and of hydrophilic or lipophilic coligands. These complexes demonstrated broader activity against both asexual and sexual parasite stages than ATV. Due to this broader effect, complexes exhibited faster action in antiplasmodial activity than ATV. Ruthenium content from the complexes’ treatment gradually and selectively accumulates in the parasite cell milieu. Efficacy was assessed *in vivo* against asexual and sexual stages. Complexes were capable of blocking parasite transmission from humans to insects, and this was achieved for both gametocytes and oocyst stages, while ATV solely blocked oocysts. This is the first example of metal complexes inhibiting both asexual and sexual parasites with similar potency, broadly expanding the therapeutic utility of metalladrugs in medicinal chemistry.

## Graphical abstract

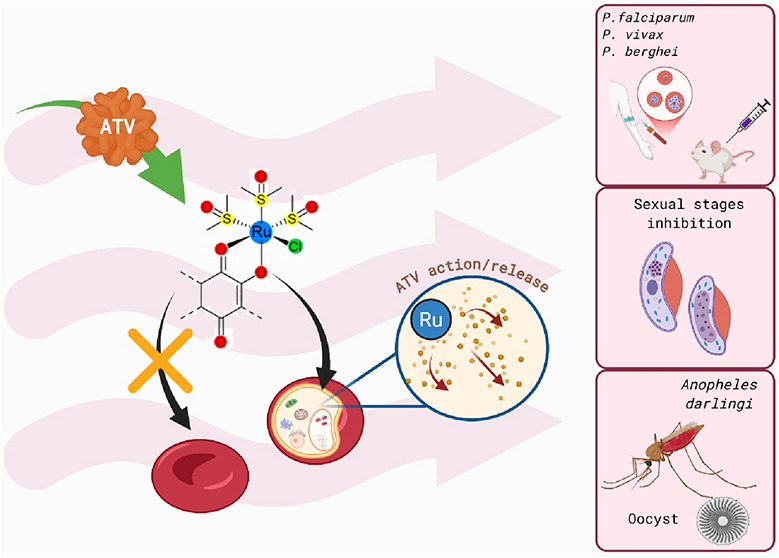

## Introduction

Malaria is an infectious parasitic disease, with an estimated 282 million cases and 610,000 deaths in 2024, mostly among children in Africa. *Plasmodium falciparum* and *Plasmodium vivax* are the most prevalent and dangerous malaria parasite species. Multidrug resistance in *P. falciparum* has spread worldwide over the last three decades. Moreover, chloroquine (CQ) resistance in *P. vivax* exists in many areas of Asia and Oceania.^1^ Therefore, there is a pressing need for the development of new drugs for treatment as well as for blocking malaria transmission.

Atovaquone (ATV), a 1,4-naphthoquinone, is a broad-spectrum antiparasitic agent against infections caused by *P. falciparum* parasites and *Pneumocystis* spp. fungi.^2^ It is clinically used for malaria prevention and treatment, and an alternative treatment for *Pneumocystis carinii* pneumonia.^2–4^ For malaria, ATV is used in association to proguanil (trade name Malarone) as a causal and suppressive prophylactic and prescribed for protecting travelers to malaria-endemic areas.^3,5^

Against the asexual blood stages (ABS) of *P. falciparum*, ATV inhibits the parasite growth at late trophozoite stage.^6–8^ Due to this stage-specific activity, ATV is considered a relatively slow-acting drug. The mechanism of action for ATV against the ABS of *P. falciparum* is well-understood and involves the binding of ATV to the ubiquinol binding site of the cytochrome *bc*_1_, located in the mitochondrion of parasite cells. ATV competes with ubiquinol, inhibiting the redox regeneration of ubiquinol: ubiquinone, and this subsequently causes a collapse in the mitochondrial electron transport system. A major lethal effect by ATV exposure is caused by the inhibition of pyrimidine biosynthesis (Figure 1).^9,10^ The capability of the parasites to modify its ubiquinone biosynthesis can cause drug resistance to ATV. For instance, mutations in the cytochrome *b* (*cytB*) can make parasites partially independent of ubiquinone biosynthesis and in turn more resistant to ATV treatment.^11^

**Figure 1:**
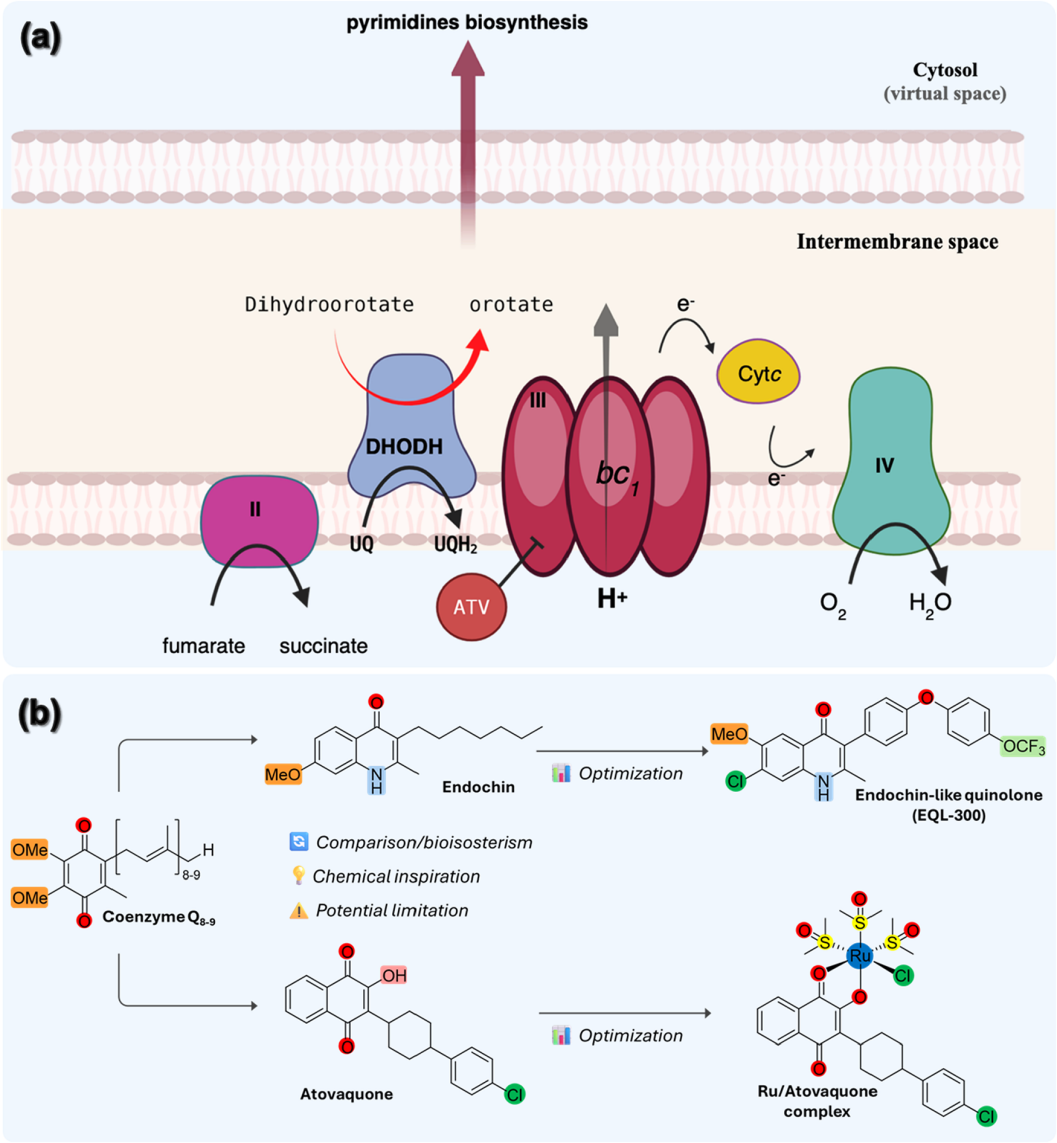
Panel (a) depicts the mechanism of action of Atovaquone (ATV) on the asexual blood stages of *Plasmodium* spp. In mitochondria, ubiquinone (UQ) functions as an electron carrier for many UQ-dependent dehydrogenases. This electron transport system is mediated by the subsequent reduction of UQ to ubiquinol (UQH_2_). Among these UQ-dependent dehydrogenases, succinate-coenzyme Q reductase (complex II) catalyzes the oxidation of succinate to fumarate as does the dihydroorotate dehydrogenase (DHODH), which oxidizes dihydroorotate into orotate. The cytochrome *bc*_1_ (complex III) and complex IV are involved in the steps of oxidative phosphorylation of ATP. The antiplasmodial drug ATV is a competitive inhibitor of ubiquinol (UQH_2_) for the quinol oxidation (Q_o_) site located in the cytochrome *b* subunit. This competition prevents UQ redox recycling, causing a collapse in the electron transport system. Panel (b) shows the structure of coenzyme Q (CoQ), an essential cofactor within the cytochrome *bc*_1_ complex of electron transport system. As a lipophilic naphthoquinone molecule, CoQ has inspired drug design as well as guided the identification of the mode of action of antimalarials. This has inspired the identification of the naphthoquinone ATV, as well as the optimization of 4­(1*H*)-quinolones, from endochin to the endochin-like quinolone EQL-300.

ATV is considered a drug with an effect against multiple stages of the *Plasmodium* spp. life cycle. In fact, there is convincing evidence from multiples laboratories that ATV can inhibit the proliferative forms of tissue schizonts (liver-stage malaria) as well as the conversion from gametocyte maturation to the zygote form.^12–15^ This may be achieved at drug concentrations that are relevant in the context of the clinical use of ATV. The exact mechanism of action of ATV against these sexual stages and tissue schizonts remains relatively rudimentary, albeit it is presumed to involve drug binding to cytochrome *bc*_1_. Exquisitely, ATV has a poor activity against the nonproliferative form of tissue schizonts (hypnozoites caused by *P. vivax* and *Plasmodium ovale* infections) and does not efficiently kill mature gametocytes of *Plasmodium* spp.^16–19^

Despite the fact that ATV is a drug of slow-acting property and with a lower barrier for the emergence of resistance within the asexual blood stages (ABS) of *P. falciparum* in comparison to other antimalarials, it offers important advantages. First, it is a drug of proven efficacy against multiple stages of the *Plasmodium* spp. life cycle. Second, there is no transmissibility of *cytB* mutant parasites by mosquitoes.^20–22^ In light of these facts, there has been a resurgence of interest in studying novel therapeutical strategies based on ATV. This includes the development of nanocarriers containing ATV,^23^ pro-drug strategy based on ATV^24^ as well as synthetic derivatives.^25,26^ Most of these approaches aim to enhance its relatively low drug bioavailability, its low water solubility, its instability under light exposure, or its excessive lipophilic property.

Recently, the utility of ATV as a ligand for forming mononuclear complexes with transition metals was described, denoting its versatility as *O*-monodentate or *O,O*-bidentate ligand, which depends on the metallic precursor employed for the synthesis.^26,27^ It was disclosed that gold­(I) or silver­(I) complexes with ATV have retained comparable antiplasmodial activity against multiple strains of the ABS of *P. falciparum* in comparison to ATV alone. Importantly, these complexes presented chemical stability in cell culture medium and did not exhibit ligand-exchange reactions prior incubation with parasites or mammalian cells. Mechanistically, it was suggested that a silver­(I) complex with ATV can result in a better antiplasmodial activity profile by displaying a lipophilicity character similar in both acid and neutral pH conditions, a feature not reproduced by ATV alone.^26^ This knowledge prompted renewed interest in not only targeting the ABS of *P. falciparum* parasites with ATV, but also targeting the sexual stages of *Plasmodium* spp. responsible for malaria transmission. To address these aims, it was hypothesized that any designed metal complex with ATV would modulate the excessive lipophilicity of ATV, achieved by coordinating ATV as an *O,O*-bidentate to provide sufficient aqueous stability. Within this complex design, ATV would behave as a redox noninnocent ligand. Ruthenium complexes with ATV were considered a promising strategy, given their potential to compare Ru­(III) and Ru­(II) species and the versatility of ruthenium in coordinating lipophilic or amphiphilic coligands. Here, novel Ru­(II) and Ru­(III) complexes containing ATV in their coordination spheres were deliberately designed.

## Results

### ATV Coordinates to Ruthenium as an *O,O*-Bidentate Ligand

To enhance the therapeutic potential of the ruthenium center for delivering the antiparasitic ATV payload inside parasite cells, the synthesis aimed to employ ATV as an *O,O*-bidentate ligand. A previous study of metal complexes with ATV showed that this bidentate ATV ligand enables efficient recognition of its molecular target while remaining tightly bound to the metal center,^26,27^ which is consistent with the literature.^28,29^ Complex *cis*-[RuCl_2_(ATV)­(dppb)] (**1**), where dppb = 1,4-bis­(diphenylphosphine)­butane, was designed to understand the contribution of Ru­(III) species, presumably capable of undergoing ligand-exchange reactions under a hypoxic environment.^29,30^ In contrast, complexes *cis*-[RuCl­(dmso-S)_2_(ATV)­(PPh_3_)] (**2**), where PPh_3_ = triphenylphosphine, and *fac*-[RuCl­(dmso-S)_3_(ATV)] (**3**), were designed to understand the contribution of Ru­(II) species, presumably more capable of undergoing ligand-exchange reactions under an oxygen-replete environment.^31,32^ This phosphine was placed in complex (**2**) to investigate the contribution of replacing an amphiphilic ligand (dmso-*S*) with a lipophilic ligand for antiplasmodial activity. In all cases, chloride was retained as a labile coligand to enable rapid substitution under biological conditions.

These complexes (**1**–**3**) were synthesized by reacting equimolar amounts of ATV with Ru­(II) and Ru­(III) precursors under an inert atmosphere at room temperature, affording neutral and air-stable solids (Figure 2A). A full description of physicochemical and the discussion of spectral characterization are provided in the supporting material section (Tables S1 and S2 and Figures S1–S33). Here, it is worth mentioning that the crystal structures of all three complexes were solved by single-crystal X-ray diffraction.

**Figure 2:**
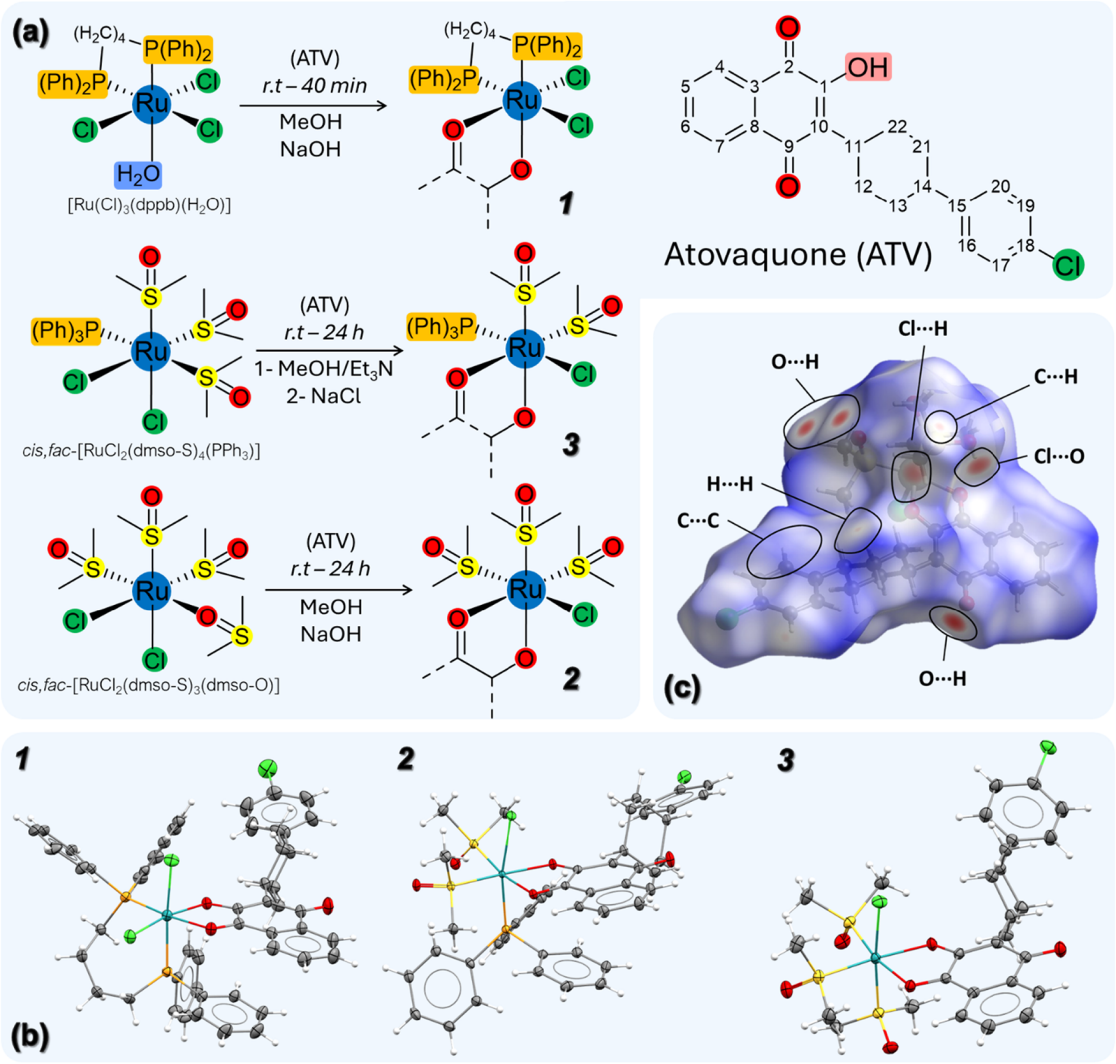
Panel (a) shows the reaction schemes and molecular structures for complexes Ru­(III)­(**1**), Ru­(II)­(**2)** and Ru­(II)­(**3**). Panel (b) shows the crystal structures of complexes (**1**–**3**), with thermal ellipsoids drawn at 30% probability. Panel (c) shows the Hirshfeld surfaces of complex (**3**) mapped with the d_norm_ function and the corresponding atom types responsible for the most relevant contacts.

The molecular structures and intra/intermolecular interactions of complexes (**1**–**3**) were solved using single-crystal X-ray diffraction from dark block-shaped crystals (Figure 2B,C and Table S1). Complex (**1)** crystallized in the monoclinic system (*P*2_1_/*n*) with a single molecule in the asymmetric unit, while complex (**2**) crystallized in the orthorhombic system (*Pbca*), containing one molecule and a dichloromethane solvent. Complex (**3**) crystallized in the monoclinic system (*P*2_1_/*c*) with two molecules in the asymmetric unit, one coordination complex, and one chloroform solvent.

All structures feature a distorted octahedral Ru coordination environment (Figure 2B). ATV coordinates as a deprotonated *O,O*-bidentate ligand through the enolate (O_1_^–^) and carbonyl (O_2_) groups, forming a five-membered chelate with bite angles below 90° (Table S2). In complex (**3)**, the remaining sites are occupied by three *fac*-arranged dmso-*S* ligands and one chlorido coligand, yielding a neutral Ru­(II) species. Complex (**2**) contains two *cis* dmso-*S* ligands and one triphenylphosphine *trans* to the chlorido, whereas in complex (**1**), neutrality is maintained by two *cis* chlorides and a dppb ligand, with P atoms *trans* to Cl_1_ and O_2_. In comparison to unbound ATV,^33^ these complexes show systematic bond rearrangements upon coordination: C_1_–O_1_ shortens, C_2_=O_2_ elongates, while C_9_=O_3_ remains essentially unchanged; all these observations were consistent with the infrared spectra data. The Ru–O distances follow the expected trend of shorter Ru–O_1_ and longer Ru–O_2_, reflecting π* back-donation into the coordinated carbonyl. This effect is accentuated in complex (**1**), where the harder Ru­(III) center interacts strongly with the enolate O1^–^, shortening Ru–O1 and lengthening Ru–O2, in line with a Pearson’s HSAB principle. These observations are consistent with previously reported ruthenium–dmso complexes containing structurally related ligands.^34^

### Chemical Stability and Metal Speciation in Solution

The stability of the ruthenium complexes in solution was investigated by UV–vis spectroscopy over 96 h in pure DMSO and in 50% DMSO/H_2_O mixtures. In DMSO, complexes (**2)** and (**3**) remained soluble, and their absorption profiles were unchanged throughout the experiment, indicating stability even in a strongly coordinating medium. An aqueous stability was also observed, despite the presence of potentially labile chlorido ligands *trans* to PPh_3_ in the case of complex (**2**) and *trans* to dmso-*S* in the case of complex (**3**). In contrast, complex (**1**) showed a distinct behavior: although soluble for the entire period of incubation, its solution color gradually shifted from deep purple to light violet, and this was accompanied by spectral modifications starting approximately 4 h after solubilization. These changes suggest partial substitution of chlorido ligands by DMSO molecules. Any reduction of Ru­(III) to Ru­(II) can be ruled out, as no ^31^P­{^1^H} NMR signals were detected even after 5 days. When it was assayed in 50% DMSO/H_2_O, complexes (**1**) and (**2**) underwent spectral changes associated with precipitation, with most of the material deposited within 5 h. Complex (**3**) remained fully soluble for at least 24 h under these mixed conditions. This time frame is close to the typical drug incubation in cell culture medium. Under these conditions, no measurable changes in the spectra were observed (Figures S28–S33).

Additional stability experiments in solution were conducted on the diamagnetic complexes (**2**) and (**3**) by monitoring their ^1^H NMR spectra for 46 h. No ^1^H NMR spectra changes were observed for either complexes in pure DMSO-*d*_6_ within the first 24 h, as no signal duplication or the emergence of new peaks was detected. This agreed with the UV–vis assays performed in the same solvent. After this period, the addition of D_2_O (10%; v/v) allowed for the evaluation of their behavior in the presence of water. Phosphine-containing complex (**2**) displayed a gradual appearance of new signals in both the aromatic and aliphatic regions (Figure S34). These changes were consistent with alterations in the chemical environment of the coordinated DMSO and ATV ligands, likely arising from speciation processes involving solvent coordination. Under the same conditions, complex (**3**) remained spectroscopically unchanged for an additional 24 h (Figure S35).

To further characterize the metal speciation pathways, chloride abstraction experiments were carried out by adding excess AgClO_4_ in DMSO-*d*_6_. Formation of an insoluble AgCl was expected to generate a vacant coordination site, subsequently occupied by a solvent molecule. In line with this reasoning, the ^1^H NMR spectrum of complex (**2**) exhibited additional signals, particularly in the aliphatic region, indicating the formation of new cationic species, which we may attribute to species such as [dmso-2]^+^ or [aqua-2]^+^. Notably, this process was time-dependent and incomplete, as part of the intact and neutral chloro complex remained unaltered (Figures S34 and S35). In contrast to complex (**2**), no spectral changes were detected for complex (**3**) upon silver addition, although a precipitation of AgCl was visually detected. This suggests that speciation occurs rapidly upon dissolution in DMSO-*d*_6_, yielding [dmso-3]^+^ as the predominant and remaining species throughout the reaction. Although the overall coordination framework in both complexes (**2**) and (**3**) was maintained, a *trans* effect played a key role in labilizing the coordinated chloride in highly coordinating media. Such ligand-exchange reactions are particularly relevant for interactions between coordination compounds and biomolecules and may directly influence their pharmacological property.^35,36^

Interaction between metal complexes and serum proteins plays a key role in modulating their metal speciation, biodistribution and intracellular transport.^37–41^ In view of this, human serum albumin (HSA) was employed as a model to assess protein-drug binding through spectroscopic approaches. Complexes (**1**–**3**) efficiently quenched the intrinsic fluorescence of HSA upon titration, as evidenced by a concentration-dependent decrease in emission intensity without spectral shifts (Figure S36). This is an indication of complex–protein interaction. Stern–Volmer analysis revealed moderate binding affinities dominated by a static quenching mechanism, which is supported by *K*q values exceeding the diffusion-controlled limit.^42,43^ The observed negative Δ*G* values have confirmed that all interactions are spontaneous (Table S3). Thermodynamic analysis showed that complexes (**1**) and (**2**) predominantly interact through hydrophobic forces, as indicated by positive Δ*S* and Δ*H* values. Differently, complex (**3**) exhibited positive Δ*S* but negative Δ*H* values, suggesting a mixed interaction mode involving both hydrophobic contributions and polar interactions, such as hydrogen bonding and electrostatic forces. These differences correlate with the molecular structures of complexes: phosphine-containing complexes (**1)** and (**2**) present enhanced hydrophobic regions, while complex (**3**) displays a higher density of electron-acceptor sites, in agreement with the crystallographic observations.^41,44^

### Complexes Have Antiplasmodial Activity against the ABS of *P. falciparum*

After chemical characterization and determination of chemical stability in solution, analysis of ATV and its complexes (**1**–**3**) was guided by *in vitro* activity against the asexual blood stages (ABS) of *P. falciparum* and mammalian cell toxicity in murine macrophages of J774 lineage and human hepatocellular carcinoma of HepG2 lineage (Tables 1 and S4).^45^ Chloroquine (CQ) was tested as a reference drug.

**Table 1:** Antiplasmodial Activity of ATV and Its Ruthenium Complexes (**1**–**3**) against the Asexual Blood Stages of *P. falciparum* ^c^ ^,^ ^d^

	*P. falciparum*, IC_50_ ± SEM [nM]^a^			
	asynchronous cultures			
compounds	NF-54	3D7	W2	synchronized into rings stages, 3D7 strain
Atovaquone, ATV	3.2 ± 1.4	1.4 ± 0.11	1.3 ± 0.6	0.53 ± 0.10
Ru(III) (1)	526 ± 126	513 ± 169	192 ± 79*	490 ± 80
Ru(II) (2)	34.0 ± 3.8	19.7 ± 2.5	45.0 ± 16.3	4.0 ± 0.66
Ru(II) (3)	13.4 ± 2.9	11.6 ± 0.70	41.0 ± 19.0	1.2 ± 0.19
[RuCl_3_(dppb)]	>1000	>1000	>1000	N.D.
Chloroquine, CQ	12.5 ± 0.67	15.5 ± 1.7	470 ± 79*	23.0 ± 2.1

	cytotoxicity for mammalian cells, CC_50_ ± SEM [μM]^b^		selectivity index (S.I.) for J774 *versus* parasites	
compounds	J774	HepG2	NF-54	3D7
Atovaquone, ATV	29.6 ± 3.3	27.8 ± 0.8	9250	55849
Ru(III) (1)	>80	>80	>152	>163
Ru(II) (2)	4.8 ± 0.19	17.0 ± 0.99	141	1200
Ru(II) (3)	2.8 ± 0.2	14.6 ± 2.0	208	2333
Chloroquine, CQ	76.1 ± 3.1	>80	6088	3308

a IC_50_ values for the growth inhibition of asexual blood stages of *P. falciparum.* NF-54 and 3D7 are drug-susceptible strains; W2 is resistant to chloroquine. Data are the mean and SEM of three independent experiments using two technical replicates. Parasites were incubation with the drugs for 72 h and growth was assessed by pLDH method.

b CC_50_ values in HepG2 hepatocarcinoma cells and J774 macrophage cell were determined after 72 h incubation and readout assessed by CellTitersGlo. Data are from three independent experiments using three technical replicates. Values are in micromolar range.

c Selectivity index (S.I.) values were calculated from cytotoxicity in J774 macrophages *versus* antiplasmodial activity in NF-54 or 3D7 strain (rings stages).

d *p < 0.05 (Mann–Whitney rank test) *versus* 3D7 strain (asynchronous culture). Abbreviations: S.I. = selectivity index. SEM = standard error of the median. CQ = chloroquine; DHA = dihydroartemisinin; ATV = atovaquone; N.D. = not determined.

ATV displayed potent antiplasmodial activity in inhibiting the growth of ABS of *P. falciparum.* It was equipotent in inhibiting the growth of NF-54 and 3D7 (CQ-susceptible) and W2 (CQ-resistant) strains. Complex *cis*-[RuCl_2_(ATV)­(dppb)] (**1**) displayed IC_50_ values of 526 ± 126 nM and 192 ± 79 nM for NF-54 and W2 strains, respectively. Therefore, it was less potent than ATV in inhibiting the parasite growth, and it was considered as an inactive molecule [34]. In contrast to Ru­(III) complex, both Ru­(II) complexes, *cis*-[RuCl­(dmso-S)_2_(ATV)­(PPh_3_)] (**2**) and *fac*-[RuCl­(dmso-S)_3_(ATV)] (**3**), were potent in inhibiting the parasite growth in low nM range. For NF-54 strain, complexes (**2**) and (**3**) have displayed IC_50_ values of 34.0 ± 3.8 nM and 13.4 ± 2.9 nM, respectively, while ATV exhibited an IC_50_ value of 3.2 ± 1.4 nM. In comparison to ATV, both complexes (**2**) and (**3**) were approximately 3- to 5-fold less potent, albeit still denoted of relevant antiplasmodial potency.^45^ Importantly, it was observed an equipotency of complexes (**2**) and (**3**) in inhibiting all three parasite strains. Further examination of antiplasmodial activity revealed that ATV and complexes were approximately 3-fold more potent when assayed on synchronized cultures at ring stages than asynchronous culture (Table 1).

Regarding mammalian cell toxicity in J774 cells, ATV presented a CC_50_ value approximately 2.5-fold lower than that observed for CQ. Both ATV and CQ are considered low cytotoxic drugs. The Ru­(III) complex (**1**) presented a CC_50_ value similar to that observed for ATV and CQ. In contrast, both complexes (**2**) and (**3**) were more cytotoxic for mammalian cells than ATV and CQ. In general, similar results were found for the cytotoxicity determined in HepG2 cells (Table 1).

Analyzing the selectivity index (S.I.) for inhibiting parasite growth *versus* affecting mammalian cell viability revealed that complexes presented values lower than ATV, which shows that they are less selective than ATV. However, when the S.I. values were calculated by using the activity determined in rings stages of 3D7 strain, a selectivity above 1000 were observed for complexes (**2**) and (**3**), which shows that these were substantially more potent to inhibit parasite growth rather than affecting mammalian cell viability. Moreover, when the activity determined in CQ-resistant parasites of the W2 strain is taken for calculating the S.I., this results in a S.I. = 161 for CQ; this also reinforces that complexes (**2**) and (**3**) present an acceptable selectivity profile as antiplasmodial agents. Neither ATV nor the complexes (**1**–**3**) caused hemolysis in uninfected red blood cells (uRBCs) up to a concentration of 10 μM (Table 2).

**Table 2:** Parameters Calculated from the Efficacy in *P. berghei*-Infected Mice (Peters’ Test)

	*P. berghei*-infected mice, median ± S.D.		
groups	drug dose in mg/kg (μmol/kg)	parasitemia reduction (%)^a^	median of survival in days (% cure)^b^
Control (CTL)			20(0)
Atovaquone, ATV	1.7 (4.6)	75.0 ± 5.1	>30(80)
Ru (III) (1)	15 (15)	52.5 ± 6.6	28(0)
Ru (II) (3)	11.5 (15)	80.2 ± 7.9	29(40)
Chloroquine, CQ	25 (78)	>99	>30(100)

a Values calculated in comparison to untreated control and taken from day 9 postinfection.

b Survival monitored by 30 days postinfection. Values in parentheses are the percentage of cure, defined as animal survival at day 30.

### Complex (**3**) Has a Fast-Acting Activity against *P. falciparum*

The phenotype of activity of ATV is its peak in activity for the late trophozoite stage. Based on this, the antiplasmodial activity was determined in tightly synchronized parasites at rings stages of 3D7 strain after 24 h, 48 and 72 h of drug exposure (Figure 3A). Dihydroartemisinin (DHA) was capable of inhibiting rings (24 h) and trophozoites (48 h) stages, and its IC_50_ values were the same between the different exposure times. When ATV was incubated for 24 h, it poorly inhibited the growth from ring to trophozoite stages. This was inferred by a fold change in IC_50_ values from 24 h *versus* 72 h of 2.1. More importantly, the nonlinear curve fitting of log-transformed data shows a poorer goodness of fit for 24 h after exposure by ATV (*R* ^2^ = 0.8789 *versus* a *R* ^2^ = 0.9943 for 72 h), and this overestimated the ATV activity at 24 h. Complex (**3**) was more efficient than ATV in inhibiting the growth from the ring to trophozoite. This interpretation was based on the observation that the IC_50_ values at 24 and 48 h exhibited a goodness of fit to nonlinear curves (*R* ^2^ = 0.9938 and 0.9957, respectively), outperforming those obtained for ATV (Figure 3B–E and Table S5).

**Figure 3:**
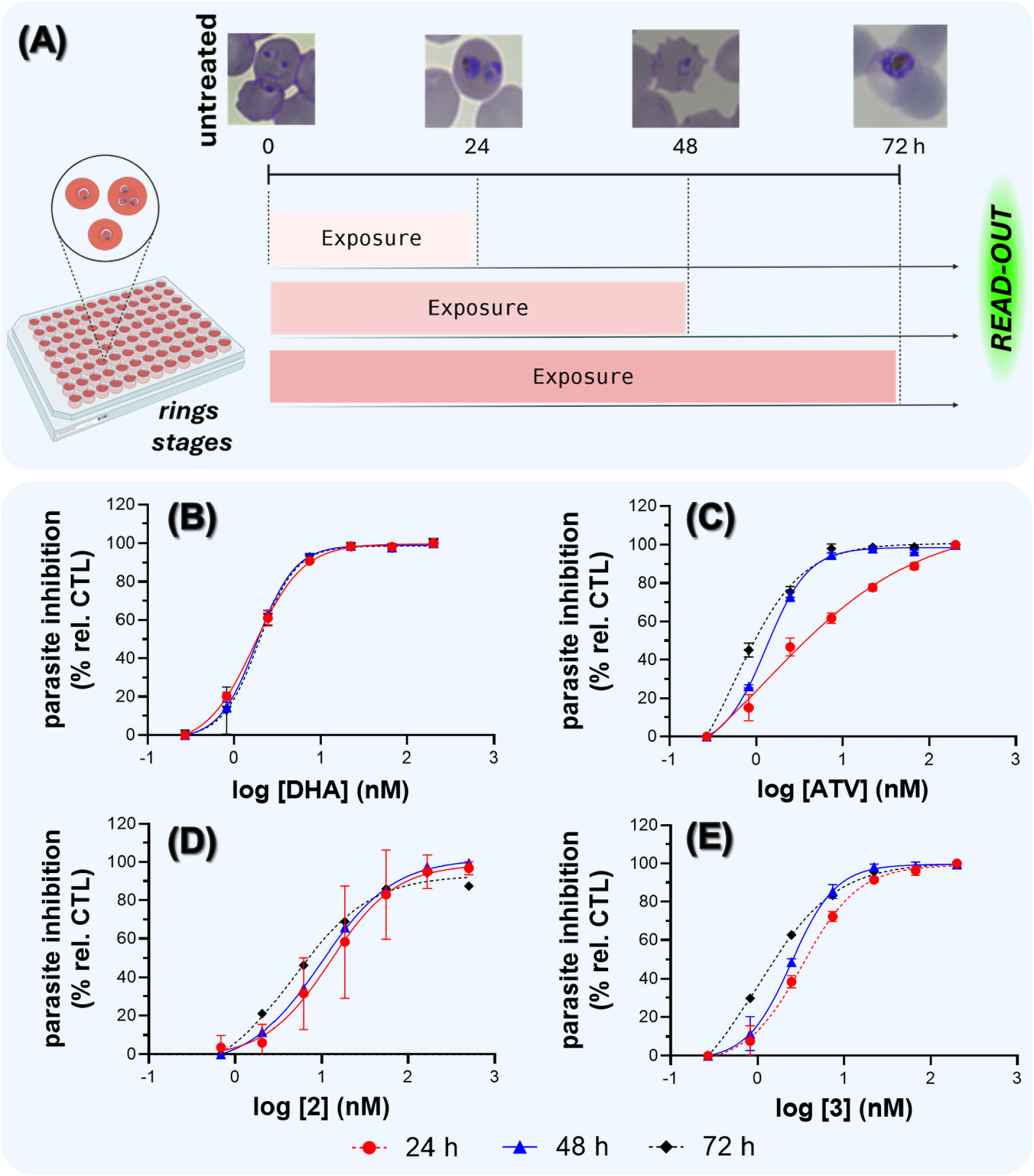
ATV and its Ru complexes (**1**–**3**) inhibit the growth of the asexual blood stages of *P. falciparum*. Panel A depicts the experimental design for determining the speed of action for asexual blood stages. Panels (B–E) show the curves of growth inhibition after 24, 48, or 72 h of drug incubation for the 3D7 strain of *P. falciparum*. Growth was assessed by SYBR green I method and dots are the mean and error bars are the standard deviation of two replicates. Abbreviations: DHA = dihydroartemisinin; ATV = atovaquone.

To confirm that complex (**3**) can inhibit the parasite development in ABS, parasite growth and morphology were visualized by Giemsa staining in thin blood slides (Figure 4). In the untreated control, parasites at 24 h have growth to late trophozoites. At 24 h after exposure with ATV, parasites displayed a morphology more similar to that of early trophozoites. In contrast, parasites under 24 h of DHA treatment presented a morphological characteristic of midrings. Similarly, at 24 h after exposure with complex (**3**), parasites presented a morphology more likely to midrings. Further determination of parasite stages in thin blood smear slides revealed that approximately 9% of parasites were at early trophozoites after 24 h of treatment with ATV (Figure S37). In contrast, no early trophozoites were identified after 24 h of treatment with complex (**3**) in thin blood smears slides.

**Figure 4:**
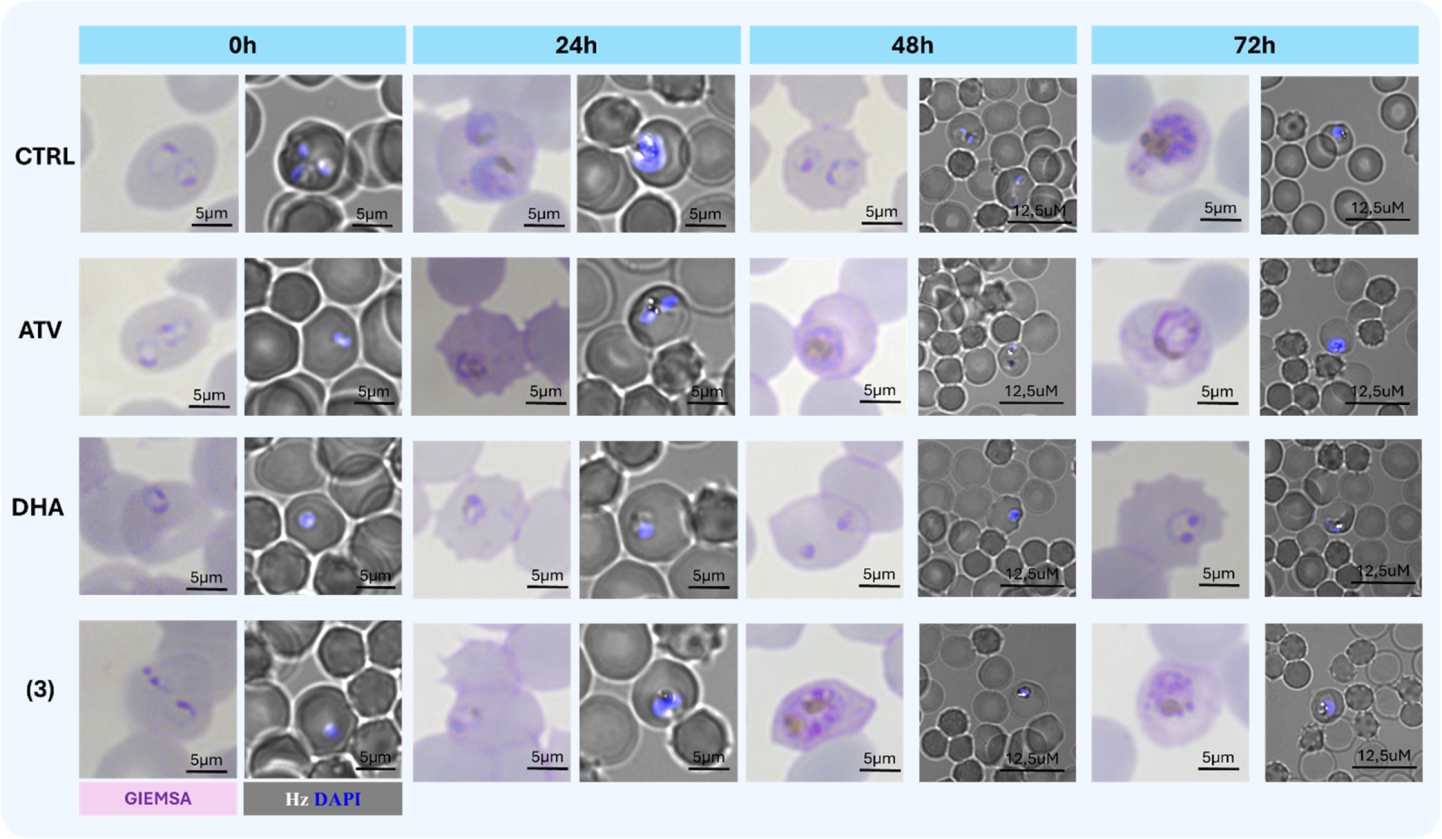
Complex (**3**) arrests the growth of ring and trophozoite stages and causes morphological alterations in parasites of the 3D7 strain of *P. falciparum*. Parasites at ring stages were incubated in the presence of drugs for 24 h, 48 or 72 h and then Giemsa staining of thin blood smear slides were mounted, or parasites were fixed, stained with DAPI and visualized by fluorescence microscopy and reflection contrast polarized light microscopy. Drugs were tested at 25 nM. Two technical replicates were employed. Parasite morphology was visualized by Giemsa-stained parasites in bright field. The presence of hemozoin (Hz) inside pRBCs was visualized by reflection contrast polarized light microscopy (birefringence) and nuclei (blue) was visualized by fluorescence microscopy. Abbreviations: CTL = untreated control; DHA = dihydroartemisinin; ATV = atovaquone; pRBCs = parasitized red blood cells; Hz = hemozoin. Calibration bars are given.

At 48 h, untreated control parasites have invaded RBCs and rings stages are observed, while under ATV treatment, both early and late trophozoites. In contrast, parasites under DHA treatment displayed arrested rings stages of condensed morphology. Under complex (**3**) treatment, parasites are at late trophozoites and presents altered morphology. After 72 h of dug exposure, both treatments with ATV and (**3**) clearly induced morphological alterations in the remaining late trophozoites. Therefore, it was observed that while ATV did not efficiently arrest the growth from rings into trophozoites, complex (**3**) arrested the parasite growth. It was inferred that complexes have a speed of activity that is faster than ATV but slower than observed for DHA (Figures 3 and 4).

Formation of hemozoin (Hz) crystals is an important hallmark of the phenotype of parasite inhibition by many antimalarial drugs.^46^ It was examined the effects of compounds on binding to hemin and inhibition on the formation of β-hematin crystals, as proxy assays of Hz formation, as well as by visualizing the Hz content in parasite cells. ATV had a relatively weak affinity for hemin, and this drug did not inhibit β-hematin formation in relevant concentrations. Likewise, these metal complexes (**1–3**) were poor inhibitors of this process (Table S4). However, in cell-based assays, it is documented that parasites under ATV treatment can produce relatively fewer (Hz) crystals in comparison to untreated control.^47,48^ Here, these Hz crystals were directed to be observed by polarized light microscopy (Figure 4). It was observed that in comparison to untreated control, parasites exposed to ATV produced smaller Hz crystals at 24 h. However, at exposure time of 48 h, smaller but remaining Hz crystals were still observed. In contrast, treatment with complex (**3**) inhibited the size of Hz crystals at 24 h and fewer Hz crystals were observed at 48 h in comparison to untreated parasites (Figure S37).

### Stage-Specificity Effects on Early Trophozoites of *P. falciparum*

A recent study determining the stage-specificity of drug susceptibility on ABS not only identified late trophozoites of *P. falciparum* as the most susceptible stage to ATV treatment, but also, reported a biphasic concentration–response in early trophozoites treated with ATV.^8^ Therefore, to confirm our above results, in which we showed a different inhibition profile of complex (**3**) against the ABS of *P. falciparum* compared to ATV, we determined the susceptibility of early trophozoites of the 3D7 strain of *P. falciparum* to ATV, complex (**3**) or CQ as a control drug (Figure 5).

**Figure 5:**
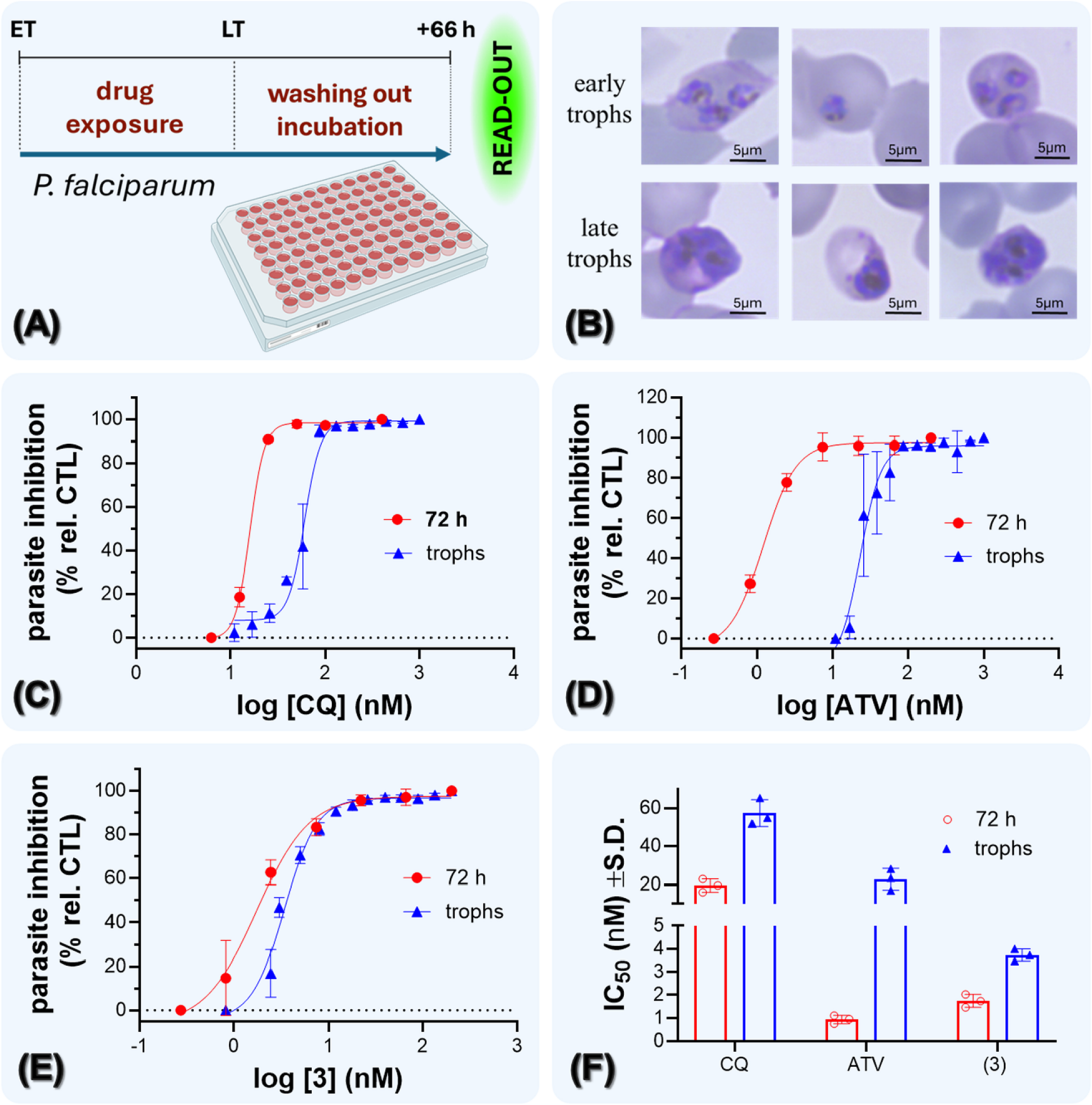
Susceptibility of early trophozoites of *P. falciparum* to treatment with ATV and the complex (**3**). Panel (A) depicts the experimental design for determining the stage-specificity in early trophozoites (trophs). Panel (B) depicts the Giemsa-stained slides of untreated parasites at the onset of drug exposure (early trophozoites) and at the point of drug removal (late trophozoites). Panels (C–E) show the growth inhibition curves for the 3D7 strain of *P. falciparum* derived from the stage-specificity assay (trophs) and standard assay for rings stages (72 h). Panel (F) shows the IC_50_ values derived from panels (C–E). Parasite growth was assessed by SYBR green I method, and dots are the mean and error bars are the standard deviation of two replicates. Abbreviations: ET = early trophs; LT = late trophs; CQ = chloroquine; ATV = atovaquone; trophs = trophozoites.

For this assay, parasite stages were carefully verified by Giemsa staining. Early trophozoites were treated with the respective drugs and incubated until the untreated early trophozoites progressed to late trophozoites (Figure 5A,B). At late trophozoites, plates were carefully washed, the cell pellets were transferred into new plates to ensure complete drug removal, and then parasites were incubated for an additional 66 h without the presence of drugs. IC_50_ values from early trophozoites were compared to standard IC_50_ values determined in rings stages (denoted as 72 h). More importantly, IC_50_ values were compared to CQ, which is a drug that has a stage-specificity to all rings and trophozoites of *P. falciparum.*

From three independent experiments, it was observed that CQ displayed a 3-fold difference in IC_50_ values from early trophozoites compared to a standard 72 h assay in ring stages. This difference is consistent with the original report of stage-specific CQ susceptibility,^8^ and may be attributable to time-dependent pharmacodynamic effects, such as the shorter duration of drug exposure between the assays. In contrast, it was observed that ATV displayed an approximately 20-fold difference in IC_50_ values from early trophozoites compared to a standard 72 h assay in ring stages, displaying a substantially reduced potency of ATV to inhibit the growth of early trophozoites. While part of this reduced potency may be attributable to time-dependent pharmacodynamic effects, a 20-fold difference is much higher than it was observed for the control drug, CQ. Based on this fold change, it was interpreted as that there is a reduced parasite susceptibility in early trophozoites for ATV. In addition to this shift in potency, we observed a difference in the shape of the curves. While we did not precisely identify a biphasic concentration–response of early trophozoites to ATV treatment as previously described,^8^ it was observed that the nonlinear curve fitting of log-transformed data has shown a poorer goodness of fit for early trophozoites than a standard 72 h (*R* ^2^ = 0.9013 for trophozoites *versus* a *R* ^2^ = 0.9900 for 72 h). Then, the effects of the complex (**3**) were examined. This treatment displayed a 2-fold difference in IC_50_ values from early trophozoites (mean 1.7 ± 0.28 nM; *R* ^2^ = 0.9759) compared to a standard 72 h assay in rings (3.7 ± 0.26 nM; *R* ^2^ = 0.9915). Based on this, it was interpreted that early trophozoites of *P. falciparum* are more susceptible to complex (**3**) treatment than ATV (Figure 5C–F).

### Complex (**3**) Has a Fast-Action Efficacy in *Plasmodium berghei*-Infected Mice

To obtain a comprehensive evaluation of the antiplasmodial activity of metal complexes, it was evaluated in *P. berghei*-infected mice using Peters’ test (Figure 6A,B and Table 2). Mice were treated *via* the intraperitoneal route at a dosage of 1.7 mg/kg (4.6 μmol/kg) of ATV, or a dosage of complexes (**1**) and (**3**) was set at 15 μmol/kg. These dosages were selected based on the estimation that complex (**3**) was approximately 3-fold less potent than ATV Against *P. falciparum*. In comparison to the untreated group, mice treated with the ruthenium­(III) complex (**1**) presented a reduced parasitemia (blood schizonts) and increased in the median in mice survival, albeit this treatment was less efficient than CQ. Treatment with complex (**3**) resulted in a suppression in parasitemia, which was more efficacious than complex (**1**). As a result of this, treatment with complex (**3**) was capable of curing 40% mice. In comparison, treatment with ATV was capable in curing 80% mice. These *in vivo* results corroborate the *in vitro* potency, where complex (**3**) is approximately 3-folds less potent than ATV.

**Figure 6:**
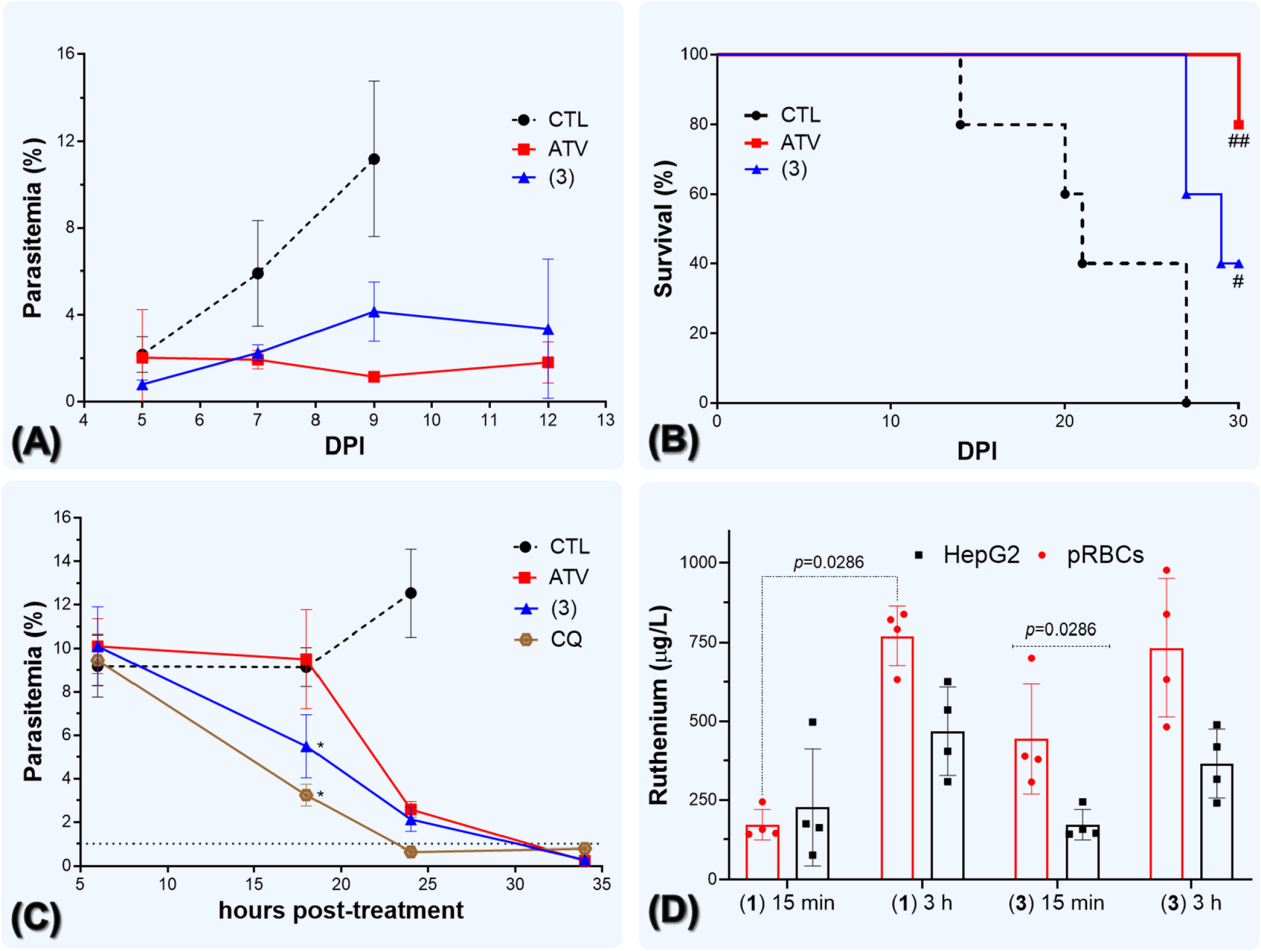
Complex (**3**) suppresses blood schizonts in *P. berghei*-infected mice, exhibits a fast onset of action, and the ruthenium content accumulates in pRBCs. Panels (A and B) show the suppressive Peters test (treatment initiated 24 h postinfection) on parasitemia and animal survival in *P. berghei*-infected Swiss mice. Drug was given daily by intraperitoneal injection for four consecutive days. Panel (C) shows the effect of a single-dose Ru (**3**) and matched ATV treatment in reducing parasitemia in *P. berghei*-infected mice. Drug dosage is indicated in Table 2. Panel (D) shows the ruthenium content in pRBCs and HepG2 cells determined by ICP-MS after drug exposure with complexes (**1**) and (**3**) at 10 μM. In panels (A–C), infection was performed in the NK65-gfp strain of *P. berghei*-infected Swiss mice (*n* = 5/group), and parasitemia was determined by flow cytometry. Panels (A and C), values are the median and error bars are the SD. Panel (D) shows the median and error bars as the SD for four technical replicates. ^#^ *p* < 0.05, ^##^ *p* < 0.01 (log-rank, Mantel-Cox test). *p < 0.05 (one-way ANOVA and Dunnett post-test). Panel D, values were calculated from one-way ANOVA and Dunnett post-test. Abbreviations: CTL, control; DPI = days postinfection; CQ = Chloroquine, ATV, Atovaquone; pRBC = parasitized red blood cells. ICP-MS = inductively coupled plasma mass spectrometry.

As the Peters’ test employs daily treatment for four consecutive days, the efficacy of complex (**3**) treatment in reducing parasitemia was examined by using one single drug dosing (Figure 6C). After 6 h of treatment, parasitemia was similar among all drugs. After 18 h, which comprehends one cycle of the *P. berghei* growth, it was observed that parasitemia was statistically different for CQ and complex (**3**) groups in comparison to the untreated group. This same observation was not observed under ATV treatment. After 24 h, which comprehends a time frame where parasite reinvasion has already occurred, it was observed that parasitemia was below the threshold of detection for CQ-receiving group, but not for treatment with complex (**3**) and ATV. It is interpreted that complex (**3**) can reduce the parasite growth faster than ATV, albeit this reduction is slower than CQ. A faster rate of antiplasmodial activity for complex (**3**) compared to ATV can overcame the observed reduction in *in vitro* potency and *in vivo* efficacy.

### Intracellular Accumulation of Ruthenium

To understand the mechanism by which the complexes exhibit fast action on ABS, we examined the contribution of the ruthenium content. First, it was observed that none of the precursors of the metal complexes presented antiplasmodial activity against the 3D7 strain of *P. falciparum* at concentrations up to 1000 nM. Second, the drug combination of the precursor of (**3**) and ATV at a 1:1 ratio did not reproduce the fast antiplasmodial activity against *P. falciparum* observed for complex (**3**) (Table S6).

Next, the intracellular concentration of ruthenium was examined after treatment by complexes (**1**) and (**3**) in parasitized RBCs (pRBCs) of *P. berghei* harvested from blood mouse as well as in HepG2 cell cultures (Figure 6D). Ruthenium concentration was quantified by inductively coupled plasma mass spectrometry (ICP-MS). Drug concentration was of 5.0 μM, and incubation times were after 15 min and 3 h. For treatment with Ru­(III) (**1**), a statically significant increase in ruthenium concentration in pRBCs was observed after 15 min to 3 h. Moreover, an increase in ruthenium concentration in HepG2 cells was observed from 15 min to 3 h, albeit this did not reach statistical significance. In contrast, there was no difference in ruthenium concentration between pRBCs *versus* HepG2 cells treated with (**1**). For treatment with Ru (II) (**3**), the ruthenium concentration was higher in pRBCs than in HepG2 cells. In contrast, an increase in ruthenium concentration from 15 min to 3 h of incubation times was less noticeable.

### Complex (**3**) Blocks the Mosquito Transmission of *P. vivax* Parasites

After having ascertained that the complexes can have strong antiplasmodial activity against the asexual blood stages and that they present a speed in antiplasmodial action dissimilar to that of ATV, the antiplasmodial activity against sexual stages was determined (Table 3 and Figure 7). First, the activity of compounds in reducing the viability of mature gametocytes of *P. falciparum* was analyzed. Methylene blue (MB) was employed as a gametocidal reference drug. It was observed that ATV has no gametocidal activity at concentrations up to 10 μM, denoting that it is inactive in this assay. In contrast, ruthenium complexes were more potent as gametocidal compounds. It was inferred that complex (**3**), the most potent against the asexual blood stages among the metal complexes, was also the most potent compound against the gametocytes of *P. falciparum*, presenting an IC_50_ value of 93 nM *versus* IC_50_ value of 60 nM observed for MB (Table 3). As a comparison, previously investigated ruthenium complexes containing antiplasmodial quinolines as ligands were several folds less potent than methylene blue against mature gametocytes.^49,50^

**Figure 7:**
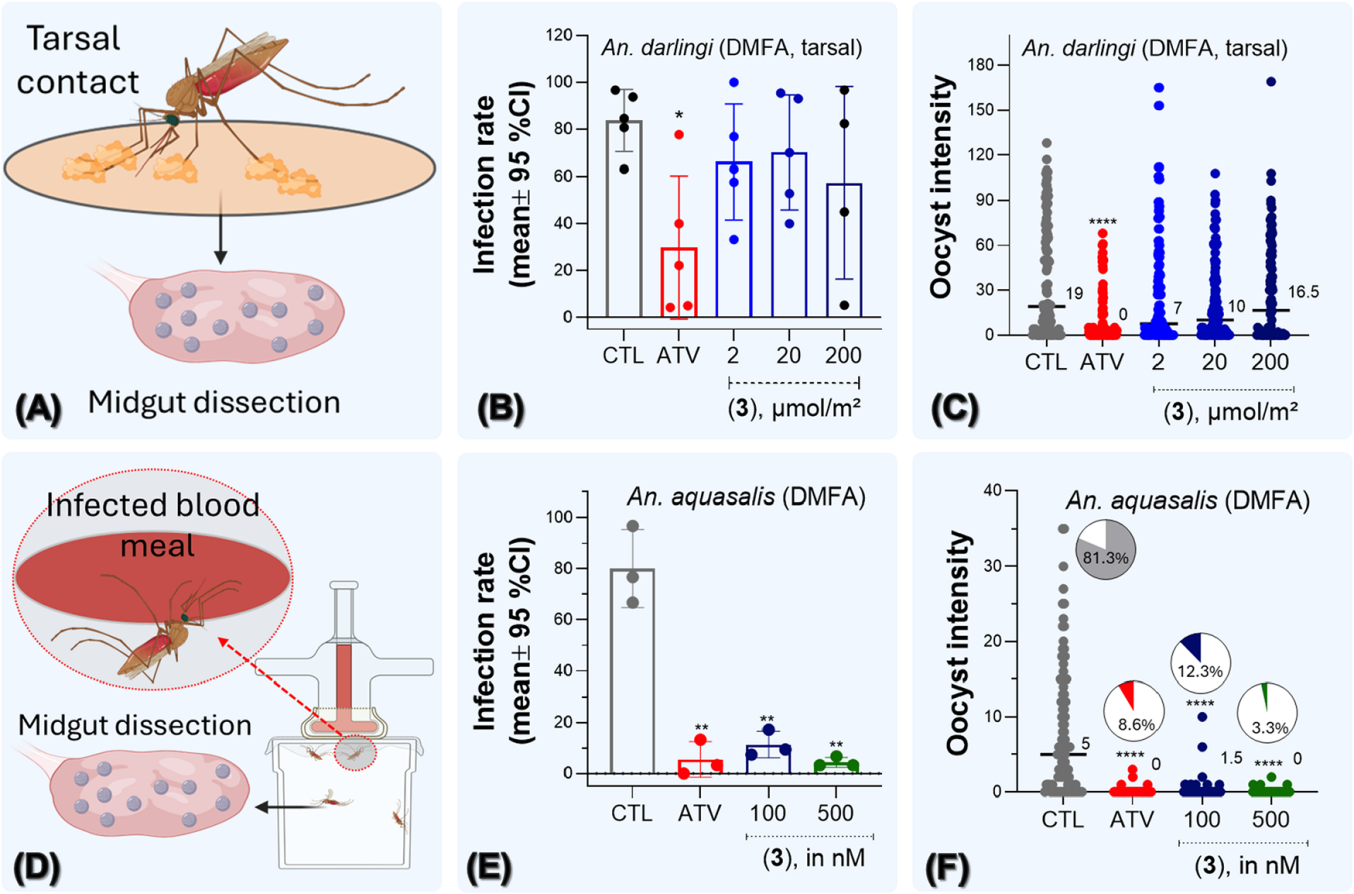
Complex (**3**) has efficacious transmission-blocking activity. Panel (A) shows a schematic representation of the direct membrane feeding assay (DMFA) from drug-coated surfaces exposure to the tarsal area of *Anopheles.* Drugs were added at range from 2 to 200 μmol/m^2^. Panel (B) shows the infection rate as the % of mosquitoes with the presence of 1 or more oocysts per midgut of assay from tarsal contact. Panel (C) shows the intensity determined as the number of oocysts per infected midgut. Panels (B and C) are pools of five clinical isolates. Panel (D) shows the DMFA by feeding of *Anopheles* with infectious *P. vivax*-blood meal containing the drugs. ATV was added at 500 nM and complex (**3**) was added at 500 and 100 nM. Panel (E) shows the infection rate, and panel (F) shows the intensity. In panel (F), pie charts illustrate the prevalence of midguts containing at least one oocyst. Panels (E and F) are from three clinical isolates. Thirty engorged mosquitoes were dissected in each group and per patient. Each midgut was dissected at 7 days postinfection to detect *P. vivax* oocysts, and oocyst intensity was determined. Each point represents the oocyst number from a single blood-fed mosquito. Median lines, values, and 95% CI values are given in panels (C and F). Supporting data are shown in Tables S7 and S8. Significance of infection rate (*versus* CTL) was calculated by one-way ANOVA with Tukey’s multiple-comparisons tests (*p < 0.05; **p < 0.01). Infection intensity (*versus* CTL) was calculated by using the Kruskal–Wallis test with Dunn’s post hoc multiple-comparisons correction (****, *p* < 0.0001).

**Table 3:**
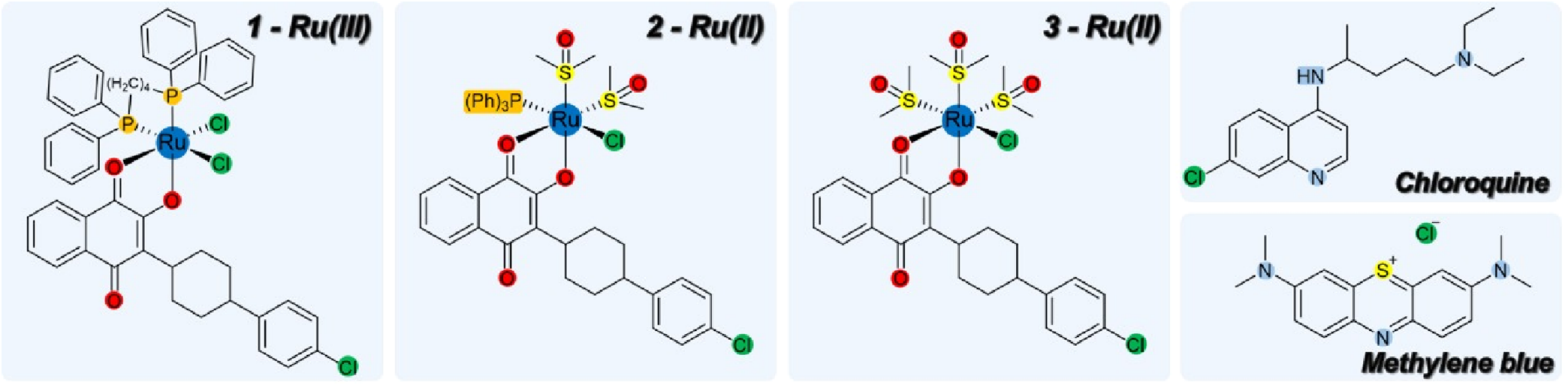
Activity of ATV and Its Ruthenium Complexes (**1**–**3**) on Mature Gametocytes of *P. falciparum* and the Hemolytic Potential in Human Uninfected Red Blood Cells (uRBCs)

compounds	gametocytes (stages IV/V) of *P. falciparum*, IC_50_ ± SD [nM]^a^	hemolysis in uRBCs (% ± S.D.)^b^	selectivity index^b^
Atovaquone, ATV	>10,000	0.57 ± 0.11	2.9
Ru(III) (1)	510 ± 200	1.0 ± 0.10	156
Ru(II) (2)	2800 ± 7300	0.70 ± 0.20	1.7
Ru(II) (3)	93 ± 22	1.2 ± 0.11	30
Chloroquine, CQ	>10,000	0.1 ± 0.1	8
Methylene blue, MB	60 ± 3	N.D.	N.D.

a IC_50_ values for the gametocytes IV/V of *P. falciparum* (3D7elo1-pfs16-CBG99 strain) determined after 72 h of incubation and viability readout determined by luminescence.

b The ratio of hemolysis in uRBCs was measured after 1 h of incubation (drugs were tested at 10 μM) and determined by absorbance reading.

c Selectivity index (S.I.) values were calculated from cytotoxicity in J774 macrophages *versus* antiplasmodial activity in gametocytes. Abbreviations: uRBC = uninfected red blood cells; N.D. = not determined; SD = standard deviation.

Given this activity against gametocytes, it was worth studying the blocking capability of the complex (**3**) to inhibit mosquito transmission of *P. vivax*. First, the endectocide activity on *Anopheles (An.) darlingi* mosquitoes was assessed by monitoring the vector survival and fertility. In comparison to the untreated control, it was observed that direct contact with ATV-coated surfaces in Petri dishes at a concentration of 200 μmol/m^2^ for 60 min on the mosquito legs (referred to as tarsal exposure) led to a reduction in the lifespan of *An. darlingi* by causing increased mortality. Mosquito fertility was not affected by ATV at this time of exposure. In this same condition, complex (**3**)-coated surfaces at a dilution series of exposures from 200 to 2 μmol/m^2^ did not cause mortality of *An. darlingi* mosquitoes (Figure S38).

Next, the activity of compounds in blocking the parasite sporogony cycle was assessed using a direct membrane feeding assay (DMFA) for *P. vivax*. Two different methods of drug exposure and delivery were employed. The first method involved the use of drug-coated surfaces exposed to the tarsal area of *An. darlingi* before the mosquitoes were allowed to feed on *P. vivax*-infected blood (Figure 7A). The second method consisted of adding drugs into the blood and allowing *Anopheles aquasalis* mosquitoes to eat blood (Figure 7D). In both methods of drug delivery, the outcome was examined by the presence of *P. vivax* oocysts in the midgut.

It was observed that *P. vivax* parasites presented a 76% reduction in infection rate in females exposed to 200 μmol/m^2^ of ATV for 60 min prior to mosquito blood meal (8.44% oocyst intensity for ATV *versus* a 36.12% for untreated control; *p* < 0.0001 by Kruskal–Wallis test followed by Dunn’s post-test). In contrast, complex (**3**)-coated surfaces at a dilution series of exposures from 200 to 2 μmol/m^2^ did not reduce the number of oocysts when compared to the control (Figure 7B,C and Table S7). A number of drugs that can inhibit transmission when added into the blood meal can fail to exhibit activity when employed in drug-coated surfaces as a drug delivery route to the tarsal area of mosquitoes.^51^

The DMFA was conducted by adding drugs into the blood and allowing *An. aquasalis* mosquitoes to be added to a blood meal. Drug dosage of ATV was of 10 μM, which is closer to a dosage of 7 μM employed for ring-survival assays in kelch-13 mutant *P. falciparum* parasites and which resembles the plasma concentration in *P. falciparum*-infected patients receiving ATV treatment.^52^ When treatment was given at 10 μM for ATV, it was observed that *P. vivax* parasites presented a reduction in 96% in infection rate, and these were efficiently reduced by complex (**3**) treatment (Table S7).

By ascertaining this drug-concentration range, DMFA was conducted using lower drug concentrations in order to examine a drug-concentration response (Figure 7E,F, and Table S8). It was observed that *P. vivax* oocysts in the intestine were efficiently reduced by complex (**3**) treatment at 0.1 μM or by ATV when given at 0.5 μM. The mean values of oocyst intensity indicated that ATV and complex (**3**) treatment almost completely eliminated infection, namely, mean values of zero observed oocysts after treatment. Subsequently, oocysts prevalence was calculated, and it was observed that in comparison to untreated control (81.3%), the oocysts prevalence was 8.6% for ATV (0.5 μM) and 3.3% for complex (**3**) at 0.5 μM. This suggests that complex (**3**) is twice as effective in reducing the prevalence of *P. vivax* oocysts as ATV (Figure 7F).

## Discussion

ATV was capable in forming metal complexes with Ru­(III) and R­(II) species *via* a *O,O*-bidentate coordination mode toward a 5-member ring. This sphere of coordination has proven to be stable in an aqueous medium. Dissociation of ATV out of these metal complexes was not observed here. Considering that ATV is an essential component for the antiplasmodial activity of these metal complexes, it was speculated that its dissociation from the ruthenium center should follow a redox noninnocent ligand behavior, namely, ATV is released chemically intact.

Variation of the electron density on the dioxo group of naphthoquinone can occur in the reduced state (hydroxyquinone) and in its oxidized form as a quinone or a semiquinonate. These different oxidation states of the ATV ligand may bear dissimilar physical properties and hence affect biological activity. In *Plasmodium* cells, it remains unclear whether ATV can undergo redox reactions. However, it is known that ATV treatment in *P. falciparum* cell culture inhibits the redox regeneration of ubiquinol: ubiquinone, and this is achieved by increasing the reduced forms of both UQ-8 and UQ-9, the respective homologues of ubiquinol and ubiquinone. Moreover, ATV is more effective in inhibiting the redox regeneration of ubiquinol: ubiquinone under low oxygen content.^9^ Presumably, this condition favors the maintenance of ATV in its oxidized state as a quinone.

The antiplasmodial activity of Ru complexes (**1**–**3**) revealed that the Ru­(II) complexes (**2**) and (**3**) were potent antiplasmodial agents, while the Ru­(III) complex (**1**) was devoid of potent activity. It is presumed that the dissociation of ATV from the Ru­(III) complex (**1**) is more difficult to achieve, hindering ATV from achieving its activity. For Ru­(II) complexes (**2**) and (**3**), ATV dissociation is more prone to occur in the parasite cellular milieu. Considering that both have similar aqueous stability, the difference in potency observed between complexes (**2**) and (**3**) may be attributed to the differences in the physicochemical properties. ATV has a high lipid solubility. The presence of phosphine coligands is well-known to increase the lipophilicity in the resulting metal complexes.^53–56^ Given that, it is possible to suggest that complex (**3**) has an ideal balance in aqueous and lipid solubility in comparison to phosphine complex (**2**), and this may explain the potency enhancement for (**3**) *versus* (**2**).

The antiplasmodial activity of Ru complexes (**1**–**3**) also revealed a shift in the phenotype of how these complexes achieved this antiplasmodial activity for the asexual blood stages of *P. falciparum*. It was observed that in contrast to ATV, which has a slow speed of activity due to its stage-specificity in killing late trophozoites, complex (**3**) had a fast speed of activity by killing all trophozoites stages. This was observed in both *in vitro* and *in vivo* models. Previously, a similar shift in cell-based phenotype of activity was observed when the structure of azithromycin, a relatively slow-acting antiplasmodial agent, was modified by attaching aryl moieties, yielding compounds with a fast speed of action.^57^ Likewise, a benzylnaphthoquinone was identified that, unlike ATV, can act as a redox cycler drug by causing an imbalance on the redox homeostasis of *P. falciparum*. Unlike ATV, this benzylnaphthoquinone displayed fast speed of action for the asexual blood stages.^58,59^ It is not clear what the underlying molecular reason for this shift from slow-acting to fast-acting drug phenotype.

Heme detoxification is an important biochemical pathway, and many antimalarial drugs exhibit a phenotype in parasite killing by inhibiting the formation of Hz crystals.^46,60^ ATV indirectly inhibits the formation of Hz crystals, but it does not completely abrogate this formation, suggesting that the inhibition of Hz is a secondary consequence of this drug’s inhibition of the cytochrome *bc*_1_ pathway. In contrast, complex (**3**) was capable of inhibiting the formation of Hz crystals in the early steps within midrings and early trophozoites. We cannot reconcile the fact that complex (**3**) can inhibit Hz crystals through inhibiting heme detoxification without exhibiting affinity for binding to hemin and for inhibiting β-hematin crystal formation. Conceivable, the phenotype of parasites presenting a few Hz contents under complex (**3**) relies on indirect mechanisms rather than on a classical mechanism of heme detoxification suppression. In support of this, we observed by using the stage-specificity assay that the complex (**3**) was more efficient than ATV in inhibiting the growth of early trophozoites into mature trophozoites. Drugs that can block the formation of Hz crystals typically present a fast speed of antiplasmodial action by killing rings and trophozoites.

One of the key questions examined here was the role of the ruthenium center in the phenotype of the antiplasmodial activity of complexes. For Ru­(III) complex, it was interpreted that the ruthenium concentration gradually increases over the time in both pRBCs and HepG2 cells, but its intracellular concentration does not correlate with the observed selectivity of complex (**1**) in inhibiting parasite growth rather than a reduction in mammalian cell viability. For the Ru­(II) complex, it was interpreted that its intracellular ruthenium concentration accumulates more in pRBCs than HepG2 cells, and therefore, the intracellular ruthenium concentration explains the observed selectivity index of the complex (**3**). The reason for a less gradual increase in the ruthenium content over time under complex (**3**) treatment is unclear. We might speculate that because it has no lipophilic phosphine ligands, the mechanism involved in its cell permeation and uptake might be different than that of complex (**1**), which contains a phosphine ligand and is more lipophilic.

An important antiplasmodial property of ATV is its effect on the sexual stages of *Plasmodium* spp. by blocking human-to-insect transmission. Consistent to the prevail literature, ATV has no potent activity in reducing the *in vitro* viability of *P. falciparum* mature gametocytes.^17^ In contrast, Ru complexes (**1**–**3**) were more potent at reducing gametocyte viability. Complex (**3**) was the most potent against both the asexual blood stages and gametocytes of *P. falciparum*, presenting a potency for gametocytes similar as observed for methylene blue (MB).

ATV has a poor activity in reducing the *in vitro* viability of mature gametocytes; however, these treated gametocytes fail to differentiate into oocysts in the mosquitoes. While the exact action of ATV on gametocytes remains unclear, it is possible that it interferes with fertilization and ookinete formation rather than exerting a direct effect on gametocytes viability. Here, the *in vivo* efficacy in blocking transmission was demonstrated in DMFA using *An. aquasalis* and *An. darlingi* for *ex vivo* *P. vivax* parasites. A similar effect of ATV has been previously demonstrated for *P. falciparum* parasites^61,62^ and it is now demonstrated here for *P. vivax* parasites. ATV was capable of inhibiting malaria transmission when drug was delivered either in coated surfaces (which resembles the insecticide-treated bed nets) or directly added into the blood meal. In contrast, complex (**3**) was capable in blocking malaria transmission when drug was delivered by directly adding into the blood. None of the treatments had a consistent effect in reducing the vectors lifespan. This is consistent to a literature showing that ATV-coated surfaces did not reduce vectors lifespan in *An. gambiae* up to 1 mmol/m^2^ for 60 min of tarsal exposure.^22^ It was observed that *P. vivax* parasites in mosquito intestine were almost eliminated by complex (**3**) treatment at 0.1 μM or by ATV when given at 0.5 μM. Collectively, the effects of ATV and complex (**3**) on malaria transmission is more likely *via* direct effects on parasite’s sporogony cycle rather than by indirect effects in reducing the vectors lifespan.^22^

Finally, it is important to discuss the potential and limitations of these chemical modifications in the context of drugs acting on cytochrome *bc*_1_ of the mitochondrial electron transport chain. From a practical standpoint, a key question is the potential impact of these established complexes of ATV as fast-acting drugs. There is no doubt that the slow-acting property of the ATV is considered its main bottleneck. Overcoming this limitation may represent an important advantage and could have significant consequences for mitigating therapy failure and the rise in drug resistance.^63,64^ In fact, from a practical standpoint, there are fast-acting antiplasmodial drugs acting on cytochrome *bc*_1_,^51,65–68^ and in line with this reasoning, our work denotes the potential of chemical modifications on ATV to modulate its phenotype in antiplasmodial activity. However, much work is necessary to fully understand the scope of this phenotype observed here for the complexes. Specifically, studies addressing the antiplasmodial activity in parasite lineages harboring mutations in *cytB*, as well as the drug interactions of Ru complexes with proguanil, are warranted.

## Conclusions

We have provided new insights into the reactivity and ruthenium coordination on ATV toward the development of novel antiplasmodial metal-based drugs. An interesting annotation was that despite being isostructural, the phenotype of antiplasmodial activity between Ru­(III) and Ru­(II) complexes was different. For the asexual blood stages, Ru­(II) complex (**3**) presented a phenotype of activity different from ATV, such as a quick-killing mechanism of action and a more efficient inhibition of early trophozoites. These support the notion that the phenotype of activity of complex (**3**) is likely to encompass a much broader range of mechanisms and targets beyond ATV’s binding to cytochrome *bc*_1_ and its effect on the mitochondrial electron transport chain.

For sexual stages, our current findings demonstrate that both ATV and the complex (**3**) can exhibit inhibitory activity. Importantly, complex (**3**) had a gametocytocidal activity for *P. falciparum*, while ATV did not. A potency enhancement of complex (**3**) *versus* ATV was observed in inhibiting *P. vivax* oocysts when added to the blood meal. However, we also noticed that in drug-coated surfaces complex (**3**) did not inhibit *P. vivax* oocysts, which indicates that its physicochemical properties are not suitable for this route of topical application. In overall, metal complexes hold promising potential for positioning as broad-acting and quick-killing antiplasmodial agents.

## Experimental Section

### Materials for Synthesis

Atovaquone (ATV), RuCl_3_·H_2_O, triphenylphosphine (PPh_3_), 1,4-bis­(diphenylphosphino)­butane (dppb), and deuterated solvents were purchased from Sigma-Aldrich and used without further purification. Supporting electrolyte for voltammetry assays was tetrabutylammonium perchlorate and it was acquired from Fluka. All the solvents were purchased from Synth, besides dichloromethane, for which it was acquired from Vetec. Metallic precursors *cis-*[RuCl_2_(dmso-S)_3_(PPh_3_)], *cis,fac*-[Ru­(Cl)_2_(dmso-S)_3_(dmso-O)] and *fac*-[RuCl_3_(H_2_O)­(dppb)] were prepared according to previous published literature.^69–72^ The 1D ^1^H (300 MHz) and ^13^C NMR (75.4 MHz) spectra were recorded on a 7.0 T Varian INOVA 300 MHz spectrometer using a 5 mm internal diameter indirect probe, while ^31^P­{^1^H} (121 MHz) and 2D ^1^H–^1^H COSY, ^1^H–^13^C HSQC and ^1^H–^13^C HMBC NMR experiments were recorded on an 11.54 T Bruker AIII 300 MHz spectrometer using a 5 mm internal diameter direct probe. Microanalysis (% C, % H) were carried using a PerkinElmer 2400 Series II equipment. All infrared spectra were recorded on the Agilent Cary 630 FTIR spectrometer in Attenuated Total Reflection (ATR) mode in the range of 4000–600 cm^–1^. Ultraviolet–visible electronic absorption experiments used a Hewlett-Packard diode array 8452A scanning spectrophotometer with DMSO and deionized water as the solvents. Electrochemical measurements were carried out using a Metrohm Autolab pgstat30 potentiostat. Platinum working and auxiliary electrodes and Ag/AgCl reference electrodes in 3.5 M KCl were used in the experiments. A solution of tetrabutylammonium perchlorate 0.1 M in dichloromethane was used as supporting electrolyte, recording the voltammograms in the region between −0.5 to 1.8 V. The anode (*E*_pa_) and cathode (*E*_pc_) potentials were obtained directly from the experimental data and the redox potential (*E*_1/2_) obtained by the arithmetic mean of Epa and Epc. Mass spectrometry of complexes was conducted on a MicroTof Bruker Daltonics on positive mode, with the capillary voltage of the electronspray set to 4.5 kV, at 180 °C, and the nebulizer pressure set to 0.4 bar. The EPR spectroscopy experiments were performed in dichloromethane solution by using a Varian E109 spectrometer operating in the X-band (9.5 GHz). Measurements were carried out both at room temperature and at 77 K. Solid-state samples of complex (**1**) in dichloromethane solutions were analyzed to evaluate possible changes in spectral features under different conditions. At room temperature, the spectra showed a single rhombic component, while at 77 K in dichloromethane, two components were detected: one major species (97%) and a minor species (3%), the latter likely arising from the trans influence of the coordinated phosphine ligand, which promotes chloride labilization and solvent or water coordination. The spectra were simulated by using the EasySpin package in the MATLAB environment to extract *g*-values, line widths, and *HStrain* parameters. Purity of all metal complexes was confirmed by elemental analysis and found to be in accordance with ACS standards (purity was >95%).

### X-ray Crystallography

The single crystals of complexes (**1)**, (**2**) and (**3**) were grown from the slow evaporation of the deuterated chloroform or dichloromethane solution inside the NMR tube with the cap on (**3** and **2**, respectively), and with methanolic solution for (**1**). Diffraction data collection for complexes (**2**) and (**3**) were performed on a Rigaku Synergy-S diffractometer, equipped with the HyPix-6000HE detector and Cu Kα radiation (λ = 1.54184 Å) from a microfocus sealed tube X-ray source. These data were collected at 100 K using the temperature controller, Oxford Cryosteam 800. Data collection for (**1**) used a Rigaku XtaLAB Mini, equipped with the Rigaku Saturn724+ detector using a fine-focus sealed X-ray tube as source for the Mo Kα radiation (λ = 0.71073 Å). This data was collected at 293 K. Collection strategies and cell refinement were carried out using the CrysAlisPro software (CrysAlisPRO, Oxford Diffraction/Agilent Technologies UK Ltd., Yarnton, England). Gaussian method for absorption corrections was employed. All structures were solved using SHELXT and the intrinsic phasing method and refined with SHELXL least-squares minimization. All non-hydrogen atoms were refined with anisotropic displacement parameters, while H atom positions were calculated from SHELXT’s riding atom model. Both SHELXT and SHELXL tools are hosted in the Olex2 software, also used to make images. The Mercury program was used to visualize, make images, calculate distances, and the Full Interaction Maps. Hirshfeld surface analyses as well as their 2D fingerprint plots were obtained using the CrystalExplorer 21.5 software package.^73–79^ The *d*_norm_ maps were scaled at −0.0504 to 1.6234 for complex (**1**), −0.2885 to 1.6744 for (**2**) and −0.2476 to 1.5927 for (**3**). 2D FP plots were made combining distances d_i_ and d_e_ in ranges (Figure S25).^80^

#### Synthesis of Complex *cis*-[RuCl_2_(ATV)­(dppb)] (**1**)

In a round-bottom flask containing 10 mL of argon-degassed methanol, 41.5 mg of atovaquone (0.113 mmol) was added at room temperature. After complete solubilization, an equimolar amount of NaOH in methanol was introduced to this yellow solution, which immediately turned red, and the mixture was stirred for 40 min under the same condition. Meanwhile, 73.5 mg (0.113 mmol) of precursor [RuCl_3_(dppb)­(H_2_O)] was dissolved in 5 mL of degassed methanol in a Schlenk flask. Atovaquone solution was then added dropwise to the precursor solution and stirred for 24 h at room temperature under an inert atmosphere. The resulting dark purple solution was concentrated under reduced pressure, and the residual solvent was evaporated to dryness. The solid obtained was redissolved in dichloromethane, precipitated with cold diethyl ether, washed with water, and dried under a vacuum over silica gel. Yield: 55.3 mg (46.8%). Elemental analysis (%) calculated for C_50_H_46_O_3_P_2_Cl_3_Ru: C 62.28; H 4.81. Found: C 61.79; H 4.66. Selected IR bands (ATR, cm^–1^): ν­(C–H) 3060; ν­(C–H) 2924; ν­(C–H) 2851; ν­(C_9_=O) 1618; ν­(C_2_=O) 1570. λ_max_ (DMSO, 4.5·10^–5^ mol/L) = 555 nm.

#### Synthesis of Complex *cis*-[RuCl­(ATV)­(dmso-S)_2_(PPh_3_)] (**2**)

A volume of 10 mL of dichloromethane was deaerated with argon and partitioned in half in Schlenk and round-bottom flasks. To the latter, 40.05 mg (0.122 mmol) of atovaquone were added and after complete solubilization, a volume of 50 μL of triethylamine was added and left stirring at room temperature for 1 h. Subsequently, 65.8 mg (0.135 mmol) of the precursor *cis-*[RuCl_2_(dmso-S)_3_(PPh_3_)] were added to the Schlenk flask and once it was completely soluble, the 5 mL of deprotonated ligand were added dropwise and reacted under the same conditions for 24 h. The dark purple solution had its volume reduced to 1 mL and precipitated with cold hexanes. The dark purple solid was filtered, washed with water, and dried in vacuum over silica, yielding 56 mg of product. Further purification procedures consisted of the solubilization of the powder in 6 mL of absolute ethanol containing an excess of NaCl, under room temperature and magnetic stirring for 8 h, and stored in the fridge overnight. A dark purple solid precipitated from the solution was filtered, washed with water, and dried in vacuum over silica. Yield: 24.7 mg (19.8%). Elemental analysis (%) calculated for C_44_H_45_O_5_PS_2_Cl_2_Ru: C 57.39; H 4.93. Found: C 57.33; H 5.19. Selected IR bands (ATR, cm^–1^): ν­(C–H) 3054; ν­(C–H) 2917; ν­(C–H) 2853; ν­(C_9_=O) 1615; ν­(C_2_=O) 1555; and ν­(S=O) 1089. ^1^H NMR (300 MHz (CDCl_3_) δ, ppm): 8.02 (1H, dd, H_4_); 7.87 (6H, m, H_*o*+*m*+*p*_); 7.71 (1H, dt, H_5_); 7.61 (1H, dd, H_7_); 7.53–7.40 (1H + 9 H, m, H_6_ + H_*o*+*m*+*p*_); 7.27–7.15 (4H, m, H_16,17,19,20_); 3.62 (3H, s, dmso methyl H_23–26_); 3.26 (1H, m, H_11_); 3.04 (3H, s, dmso methyl H_23–26_); 2.73 (3H, s, dmso methyl H_23–26_); 2.60 (3H, s, dmso methyl H_23–26_); 2.50 (1H, m, H_14_); 2.35–1.54 (8H, m, cyclohexane CH_2(12,13,21,22)_). ^13^C NMR (75.4 MHz (CDCl_3_) δ, ppm): 200.44 (C_9_=O); 183.06 (C_2_=O); 168.91 (C_1_–O); 146.32 (Ar–C_18_-Cl); 136.04 (Ar–C_5_H); 135.25 (3× *o*-PPh_3_-Ar–CH); 135.12 (3× *o*-PPh_3_-Ar–CH); 133.62 (Ar–C_8_); 133.13 (C_10_); 133.01 (C_15_); 130.49 (Ar–C_6_H); 130.46 (s-PPh_3_–Ar-C); 129.35 (Ar–C_3_); 128.49 (6× *m*-PPh-Ar–CH); 128.33 (2× Ar–C_19,20_H); 128.27 (2× Ar–C_16,17_H); 128.14 (3× *p*-PPh_3_-Ar–CH); 126.83 (Ar–C_4_H); 125.93 (Ar–C_7_H); 47.92/47.07 (dmso methyl C_25,26_); 45.56/44.34 (dmso/methyl C_23,24_); 44.08 (C_14_H); 34.99/34.72/30.34/29.75 (cyclohexane C_12,13,21,22_H_2_). ^31^P­{^1^H} NMR (121.4 MHz (CHCl_2_, D_2_O capillary) δ, ppm): 41,17. λ_max_ (DMSO, 2.16·10^–5^ mol/L) = 594 nm. ESI­(+)-MS (ACN, H_2_O) [M + H]^+^ = 921.0960 *m*/*z*; [M – Cl]^+^ = 885.1186 *m*/*z*.

#### Synthesis of Complex *fac*-[RuCl­(ATV)­(dmso-S)_3_] (**3**)

In a round-bottom flask containing 10 mL of previously argon-deaerated methanol, 41.5 mg of atovaquone (0.113 mmol) were added at room temperature and after complete solubilization, an equimolar amount of triethylamine (15.0 μL; 0.108 mmol) was added to this yellow solution, which immediately turned red, and left to react for 40 min, under the same condition. Meanwhile, 54.9 mg (0.113 mmol) of the precursor *cis,fac*-[Ru­(Cl)_2_(dmso-*S*)_3_(dmso-*O*)] were solubilized in 5 mL of deaerated methanol in a Schlenk flask. The atovaquone solution was added dropwise to the solution containing the precursor and left to react under the same conditions for 24 h. This final dark purple solution had its volume reduced under pressure, and the remaining solvent evaporated until dry. The dark purple solid was resolubilized in dichloromethane, precipitated with the addition of cold diethyl ether, washed with water, and dried under vacuum over dried silica. Yield: 35.5 mg (42.5%). Elemental analysis (%) calculated for C_28_H_36_O_6_S_3_Cl_2_Ru: C 45.65; H 4.92. Found: C 45.65; H 5.01. Selected IR bands (ATR, cm^–1^): ν­(C–H) 3015; ν­(C–H) 2915; ν­(C–H) 2858; ν­(C_9_=O) 1609; ν­(C_2_=O) 1561; ν­(S=O) 1095. ^1^H NMR (300 MHz (CDCl_3_) δ, ppm): 8.03 (1H, dd, H_4_); 7.90 (1H, dd, H_7_); 7.73 (1H, dt, H_5_); 7.54 (1H, dt, H_6_); 7.28–7.15 (4H, m, H_16,17,19,20_); 3.73 (3H, s, dmso methyl H_23–28_); 3.63 (3H, s, dmso methyl H_23–28_); 3.51–3.50–3.45 (9H, 3s, dmso methyl H_23–28_); 3.29 (1H, tt, H_11_); 3.20 (3H, s, dmso methyl H_23–28_); 2.51 (1H, tt, H_14_); 2.36–1.54 (8H, m, cyclohexane CH_2(12,13,21,22)_). ^13^C NMR (75.4 MHz (CDCl_3_) δ, ppm): 198.90 (C_9_=O); 183.28 (C_2_=O); 169.11 (C_1_–O); 146.24 (C_18_–Cl); 136.31 (Ar–C_6_H); 132.87 (Ar–C_8_); 132.39 (C_10_); 131.70 (Ar–C_5_H); 131.58 (Ar–C_15_); 129,05 (Ar–C_3_); 128.54 (2× Ar–C_19,20_); 128.32 (2× Ar–C_16,17_); 127.00 (Ar–C_4_H); 126.04 (Ar–C_7_H); 48.15/47.50 (dmso methyl C_25,26_); 47.21/46.62 (dmso methyl C_23,24_); 47.01/44.09 (dmso methyl C_27,28_) 44.03 (C_14_H); 34.76 (C_11_H); 35.02/34.59/30.14/29.66 (cyclohexane C_12,13,21,22_H_2_). λ_max_ (DMSO, 3.88·10^–5^ mol/L) = 577 nm. ESI­(+)-MS (DMSO, MeOH) [M + H]^+^ = 737.0155 *m*/*z*.

### Human Serum Albumin (HSA) Binding Experiments

Interaction between HSA (Sigma-Aldrich) and complexes were performed by a fluorescence quenching experiment, where the concentration of HSA in buffer (4.5 mM Tris–HCl, 0.5 mM NaOH, and 50 mM NaCl) at pH 7.4 and maintained constant (2.5 μM), while the concentration of the complexes was increased from 2.5 to 20 μM. Extinction of the emission intensity of the HSA tryptophan residues at 305 nm (excitation wavelength 270 nm) was monitored at 298 and 310 K. Data were analyzed by using the classic Stern–Volmer equation (eq 2)­

1 $$\frac{F_{0}}{F}=1+K_{\mathrm{SV}}\left[Q\right]=1+k_{q}\tau_{0}\left[Q\right]$$

where *F*_0_ and *F* correspond to the fluorescence intensities in the absence and presence of the quencher, respectively; [Q] is the concentration of the quencher; and *K*_sv_ is the Stern–Volmer quenching constant. The binding constant (*K*_b_) as well as the number of binding sites (*n*) was determined by plotting the double log graph of the fluorescence data using eq 2

2 $$\log\left[\frac{F_{0}-F}{F}\right]=\log K_{b}+n\log\left[Q\right]$$

The thermodynamic parameters Δ*H*, Δ*S*, and Δ*G* were obtained by using eqs 3 and 4

3 $$\ln\left[\frac{K_{2}}{K_{1}}\right]=\left[\frac{1}{T_{1}}-\frac{1}{T_{2}}\right]\frac{\Delta H}{R}$$

4 $$\Delta G=-\mathrm{RT}\ln K_{b}=\Delta H-T\Delta S$$

where *K*_1_ and *K*_2_ are the binding constants at temperatures *T*_1_ and *T*_2_, respectively; and *R* is the gas constant.

### Drugs and Materials for Biological Assays

Atovaquone, chloroquine, and methylene blue were purchased from Sigma-Aldrich. For the blood stages, each drug was dissolved in dimethyl sulfoxide (DMSO) and diluted in RPMI-1640 medium into seven different concentrations. Thin blood smears were stained with Panotico Rápido (LB LABORCLIN, Paraná, Brazil) and dissected intestines of Anopheles spp. mosquitoes were stained with mercurochrome solution (Merbromin).

### Determination of Cytotoxicity for Mammalian Cells (CC_50_)

Cell toxicity was assayed for murine macrophage J774 and human hepatocellular carcinoma HepG2. Cell lines were maintained in RPMI-1640 (HepG2) and in DMEM (J774) containing 10% fetal bovine serum and supplemented with l-glutamine, vitamins, and amino acids in 75 cm^2^ flasks at 37 °C, with the medium changed twice weekly. Cell cultures from 60% confluence were trypsinized, washed in complete medium, and 4 × 10^4^ cells were plated in 100 μL per well with complete medium in 96-well flat-bottom white plates for 24 h at 37 °C. Afterward, compound and the reference drugs covering six different concentrations at 2-fold dilutions in cell culture medium were added to the wells, and plates were incubated for 72 h at 37 °C. Cell viability was determined using Cell-titer-glo kit (Promega, USA). Luminescence was read at Molecular Probe FilterMax F3 microplate reader. CC_50_ values were calculated using nonlinear regression analysis of a log­(inhibitor) *versus* response function in Prism 8 for MacOS. Three independent experiments for each cell line were performed, and three technical replicates of each drug concentration were employed.

### Determination of Antiparasitic Activity for Asexual Blood Stages of *P. falciparum* (IC_50_)

parasites (NF-54, 3D7 and W2 strains) were maintained in culture in RPMI-1640 supplemented with 0.5% AlbuMAX II (ThermoFisher, Waltham, MA) and buffered with 25 mM HEPES and 25 mM NaHCO_3_. Parasites were grown in O-positive human blood under controlled atmospheric conditions of 5% O_2_, 5% CO_2_ in N_2_ at 37 °C with 95% humidity. A volume of 100 μL of drug and 100 μL of parasitized red blood cells from asynchronous culture (0.5% final parasitemia and 2.0% hematocrit) was distributed per well into 96-well plates. Plates were incubated for 72 h at 37 °C in a controlled atmosphere. Controls without drugs or without parasites were included. Parasite growth was determined using the method of parasitic lactate dehydrogenase (pLDH). In brief, cell pellets from the plates were carefully resuspended, and a volume of 20 μL aliquots were removed and added to 100 μL of Malstat reagent in a 96-well microplate. The Malstat reagent was made by a solution of 0.125% Triton X-100, 130 mM lactate, 30 mM Tris buffer, and 0.62 μM 3-acetylpyridine adenine dinucleotide. A volume of 20 μL of nitro blue tetrazolium (NTB, 1.9 μM) and 0.24 μM phenazine ethyl sulfate were added to the plate. Plate was read at an NBT OD of 650 nm using a Synergy 4 microplate reader (BioTek, Santa Clara, USA). The concentration at which the drugs were able to inhibit 50% parasite growth (IC_50_) was calculated using the inhibitory effect sigmoid Emax model, estimating the IC_50_ value through nonlinear regression using a standard function of the software package R (ICEstimator version 1.2). IC_50_ values were calculated in three independent experiments with each drug concentration in two technical replicates.

### Activity against the Gametocytes of *P. falciparum*

The parasite line 3D7elo1-pfs16-CBG99 of *P. falciparum* was employed. Gametocytes were obtained from cultures of asexual parasites by increasing parasitemia without the addition of fresh red blood cells. Gametocyte stage was determined by Giemsa. Drugs were serially diluted at a concentration range of 10–0.05 μM in a volume of 100 μL per well in a 96-well flat-bottom plate. Then, a volume of 100 μL 3D7elo1-pfs16-CBG99 gametocytes at 0.5–1% parasitemia and 2% hematocrit were dispensed into a 96-well flat-bottom plate. Each drug was tested in duplicate and in seven different concentrations. The employed DMSO concentration was not toxic to the gametocytes. Methylene blue was used as a positive control. Plates were incubated for 72 h at 37 °C in a controlled atmosphere (1% O_2_, 5% CO_2_ in N_2_ at 37 °C). Afterward, a volume of 100 μL of culture medium was removed from each well to increase hematocrit and a 70 μL resuspended culture was transferred to a black 96-well plate. A volume of 70 μL of d-luciferin (prepared 1 mM in citrate buffer 0.1 M, pH 5.5) was added to reach a final concentration of 0.5 mM of D-luciferin. Luminescence measurements were performed after 10 min with a 500 ms integration time (multiplate luminometer, model Synergy 4, Biotek). Luciferase activity was taken as a measure of gametocyte viability, and IC_50_ was extrapolated from the nonlinear regression analysis of the concentration–response curve. Percentage gametocyte viability was calculated as 100 × ([OD treated sample–OD blank]/[OD untreated sample−μc-blank]), where “blank” was the sample treated with 500 nM methylene blue, which completely kills gametocytes. Two independent experiments were employed.

### Drug-Induced Hemolysis

Freshly collected uninfected human O^+^ erythrocytes (uRBC) were washed three times with sterile phosphate-buffered saline (PBS), adjusted for 1.5% hematocrit, and 100 μL was dispensed in a 96-well round-bottom plate. Then, a volume of 100 μL of drugs previously diluted in DMSO and suspended in PBS were dispensed in the respective wells. Each drug was assayed in triplicate at 10 μM. Untreated cells received 100 μL of PBS containing 0.25% DMSO (negative control), while positive controls received saponin (Sigma-Aldrich) at 1% v/v. Plates were incubated for 1 h at 37 °C under 5% CO_2_. Plates were centrifuged at 1500 rpm for 10 min, and 100 μL of supernatant was transferred to another plate, in which absorbance was measured at 540 nm using a Molecular Probe FilterMax F3 microplate reader. Percentage hemolysis was calculated in comparison to the untreated control and plotted against drug concentration generated using Prism. One single experiment was performed.

### Determination of the Speed of Activity in *P. falciparum* Parasites

rings stages of the 3D7 strain were obtained after two cycles of synchronization with d-sorbitol. In round-bottom 96-wells plates, a volume of 100 μL of drug and a 100 μL of parasitized RBC (ring stages) at a final parasitemia of 1.0% and 2.0% hematocrit were dispensed. Plates were incubated at 37 °C in a standard gas mixture. At each time frame of 24 h and 48 h, plates were centrifuged, supernatant was removed and replaced by fresh supplemented medium without drugs and returned for the incubator. One plate was maintained without medium replacement (denoted as a 72 h plate). All plates were maintained in the incubator for a total of 72 h and then these were frozen at −20 °C. Parasite growth was determined by the SYBR green I readout method. Briefly, a solution of SYBR green I (ThermoFisher Scientific) were prepared at a 2× concentration in a lysis buffer (20 mM TRIS base, pH 7.5, 20 mM EDTA, 0.008% w/v saponin, 0.08% w/v Triton X-100). After thawing, a volume of 100 μL of cell lysate was collected and transferred to a new black plate, a volume of 100 μL of SYBR green I solution was added, and the plates were incubated for 1 h at room temperature in the dark. Fluorescence was measured at 485 nm excitation and 528 nm emission in a microplate reader (Molecular Probe FilterMax F3 microplate reader). IC_50s_ values were as described above. Two independent experiments were performed using replicates of each drug concentration.

### Parasite Visualization by Microscopy

In a 24-well plate, parasites at rings stages of *P. falciparum* (3D7 strain) were dispensed to a final 1% parasitemia and 2% hematocrit in a 1 mL volume. Each drug concentration was added in two different wells, at a final concentration of 25 nM. Atovaquone was tested at 25 and 100 nM. Plates were incubated at 37 °C in a standard gas mixture. At each indicated time, the cell suspension was aspirated and centrifuged, and the cell pellet was divided in two parts. One part was employed for mounting thin blood smear slides and stained with Giemsa (Panotico Rápido). Another part of the cell pellet was suspended in glutaraldehyde (0.0025% in PBS, v/v) for 1 h at room temperature and then kept at 4 °C until analysis (within a 24 h time frame). Fixed parasite cells were mounted in slides and stained with ProLong Glass Antifade Mountant with NucBlue Stain (Invitrogen). Nuclei were visualized in a DAPI channel, and hemozoin crystals were visualized by reflection contrast polarized light microscopy (DMi8 S inverted microscope, Leica, Germany). Giemsa-stained slides were visualized by bright field in this same microscope by using another camera.

### Affinity Constant for Hemin (log *K*) and β-Hematin Inhibitory Activity (BHIA)

It was performed as described in reference 49.

### Stage-Specificity Activity for Early Trophozoites

It was performed as described in reference 8.

### Mice

Male Swiss-Websters were housed at Instituto Gonçalo Moniz (Fiocruz Bahia, Brazil), maintained in sterilized cages under a controlled environment, receiving a rodent balanced diet and water *ad libitum*. All experiments were conducted in 2019 in accordance with the recommendations of Ethical Issues Guidelines and were approved by the Animal Ethics Committee at Fiocruz Bahia (IGM, Salvador, Brazil), reference number 020/2018.

### *In Vivo* Blood Schizontocidal Activity

Male Swiss mice (4–6 weeks) were infected by intraperitoneal injection of 10^6^ *P. berghei-*infected erythrocytes (strain NK65/GFP) and randomly divided into groups of five. Each drug was solubilized in DMSO/dispersant solution (5:95, v/v) prior to administration. A dispersant solution was prepared using Kolliphor (Cremophor, 2%), Polysorbate 80 (2.5%), D-Sorbitol (2.5%), glucose (5%), and Tween 20 (0.5%) to a remaining volume in phosphate buffer solution (PBS 1×). Treatment was initiated within 24 h postinfection and given daily for four consecutive days by intraperitoneal injection of a volume of 100 μL. Chloroquine phosphate (25 mg/kg) was used as a positive control group, while untreated infected mice were used as a negative control group. The following parameters were evaluated: parasitemia at 5, 7, 9, and 12 days postinfection, and a 30-day postinfection (DPI) survival. Parasitemia was determined by flow cytometry by gating GFP^+^ parasites and costaining with Mitotracker deep red FM (20 nM for 30 min). To ensure a humane ending-point, any mouse displaying symptoms of severe anemia was euthanized prior the 30 DPI follow up. Percentage reduction of parasitemia was calculated as [(mean vehicle group) – (mean treated group)/(mean vehicle group)] × 100%). Each experiment was performed using no more than four groups.

### Quantification of Ruthenium Content in pRBCs and HepG2 Cells by Inductively Coupled Plasma Mass Spectrometry (ICP-MS)

For the experiments using pRBCs, Swiss mice (male and female, 20–25 g) were infected with *P. berghei-*infected erythrocytes (NK65 strain). After parasitemia reaches a 10%, mice received anesthesia (tribromoethanol, 200 mg/kg, intraperitoneal), and blood was gently aspirated from the brachial plexus using a heparin-coated tip and transferred into heparinized vials. Blood was washed with saline, cell culture and then dispensed in 12-well plates. A volume of 100 μL drug (at 10 μM) and a 900 μL of parasitized RBCs in RPMI medium supplemented with 5% fetal calf serum at a final parasitemia of 5.0% and 3.0% hematocrit were dispensed. Plates were incubated at 37 °C in a standard gas mixture for 15 min or 3 h. Samples were centrifuged at 500 rpm at 8 °C for 5 min, supernatant was removed, and cell pellet was washed twice with incomplete cell culture. Cells from this pellet were counted, and a suspension in sterile saline was prepared to a final number of 1 × 10^9^ cells/mL and then transferred into a clean 1.5 mL HPLC vial tubes and maintained at −80 °C until analysis. For the experiments using HepG2, cells were cultivated in 6-well plates at 5 × 10^7^ cells for 24 h at 37 °C. Afterward, drugs were added (at 10 μM) and incubated for 15 min or 3 h at 37 °C. Trypsin-EDTA was then added, and cells were removed with the assistance of a cell scraper. Cell suspension was transferred to a new vial, washed with RPMI medium, then saline. Cells from this pellet were counted in a Neubauer chamber, and a suspension in sterile saline was prepared to a final number of 2 × 10^7^ cells/mL and then transferred into clean 1.5 mL HPLC vial tubes and maintained at −80 °C until analysis.

A volume of 400 μL of each sample was added in a HNO_3_ solution at 1% (v/v). Samples were digested for 24 h at 60 °C. These resulting samples were then then diluted to a 1:50 ratio using a solution of HCl (1.0%, v/v) and further diluted to a 1:50 ratio with ammonium hydroxide (1.0%, m/v), EDTA and Triton X-100 (both at 0.05, % m/v). The same procedure was carried out for the standard solution of ruthenium and blanks. All determinations of metal content were conducted by monitoring the mass signals (^101^Ru, ^102^Ru and ^104^Ru) on a NeXion 300D ICP-MS (PerkinElmer) equipped with a concentric nebulizer and a Scott double pass spray chamber. Concentration of Ru in each sample was calculated from an analytical standard curve using a linear regression.

### *Anopheles* spp. Colony

Mosquitoes of the species *An. aquasalis* and *An. darlingi* were obtained from distinct colonies established at the approved insectary of Unidade de Entomologia Nelson Ferreira Fé at Fundação de Medicina Tropical Dr. Heitor Vieira Dourado (FMT-HVD, Manaus, Brazil). These colonies were maintained at constant temperature (26–28 °C) and relative humidity of 70–80%, under a 12:12 photoperiod. Larvae were hatched in room temperature water and fed with fish food (TetraMin). Larvae were allowed to pupate and emerge into adults in an enclosed mesh-covered cage with water and 10% sucrose to *An. aquasalis* or 15% of honey solution to *An. darlingi* at *ad libitum* condition. Female *Anopheles* spp. used for the experiments were 4–6 days old.^81,82^

### Recruitment and Samples of *P. vivax*-Infected Patients

Patients were recruited after formal consent at FMT-HVD in Manaus, Brazil. These were diagnosed with a monoinfection of *P. vivax* malaria by presenting a parasitemia higher than 1000 parasites per microliters of blood. Criteria included nonpregnant and in the absence of antimalarial treatment in the last 60 days. A ten mililiters volume of peripheral blood was collected from each patient in heparin tubes. After blood collection, patients received antimalarial treatment as established by the Brazilian Malaria Guidelines.^83^ All patients with *P. vivax* infection included in the project gave written informed consent in protocols approved by the FMT-HVD ethical board committee (CAAE: 57291122.5.0000.0005, approval number 5.357.919).

### Drug Exposure to *An. darlingi*

After the first dilution in DMSO, respective drug stocks were solubilized in the desired concentrations using pure acetone as a vehicle. One milliliter of each dilution was added for impregnation in glass Petri dishes with a diameter of 150 × 25 mm^2^ in the following concentrations: 200 μmol/m^2^, 20 μmol/m^2^ and 2 μmol/m^2^. One Petri dish was impregnated solely with acetone as a control group. The Petri dishes remained on a shaker (MARCONI) until the volatile vehicle evaporated and then stored at 4 °C until the day of performing the experiment of insect exposure to drug-coated surfaces (drug impregnation). For the exposure of mosquitoes, a translucent plastic container was placed on the impregnated surface and sealed with tape and cotton to prevent females from escaping during exposure. A hole was opened in the central part to allow the introduction of mosquitoes and removing after exposure. Groups containing at least 80 *An. darlingi* females were placed in contact with the surface for 60 min, shaking every 15 min to prevent them from resting on the walls without the drug. Subsequently, each group was transferred to larger cages to perform direct membrane feeding assay (DMFA), survival or fecundity assays.^22,81^ Two independent experiments were performed.

### Mosquito Fecundity and Survival

After mosquito exposure to drug-coated surfaces (drug impregnation), 30 *An. darlingi* females from each group were transferred to larger cages previously identified in a room with controlled temperature (26–28 °C) and humidity (70–80%) and with sucrose or honey solution at *ad libitum* conditions. The daily mortality of each group was counted for 15 days. Four independent experiments were evaluated. To evaluate fertility, 30 *An. darlingi* females from each group exposed to different concentrations were fed with uninfected blood. After 3 days of exposure, 20 females from each exposed and control group were forced to lay eggs, placing each female individually inside a Petri dish (35 mm × 10 mm) with filter paper moistened with water. Between 24 and 72 h, the number of eggs were counted using a magnifying glass. Three independent experiments were performed for fecundity and two for survival.

### Direct Membrane Feeding Assay (DMFA) from Drug-Coated Surfaces

A volume of 1.0 mL of blood collected in heparinized tubes containing *P. vivax* was offered to around 80 *An. darlingi* females deprived of 10% sugar solution for 24 h and immediately exposed for each drug concentration as previously described in drug exposure methodology.^82^ After feeding for 60 to 120 min, the engorged females were separated into larger cages and kept in a room with controlled conditions (26–28 °C temperature; 70–80% humidity) and with sugar water at *ad libitum* conditions. Seven days postinfection, specimens from each group had their intestines dissected in PBS 1×, stained with a commercial 2% mercurochrome solution (Merbromin), the intestines were observed under an optical microscope, and the number of oocysts was counted. Thirty engorged mosquitoes were dissected in each group and per patient. The infection rate (prevalence) was determined by the number of intestines containing one or more oocysts divided by the total number of dissected intestines. The intensity of infection was determined by means of oocysts in the dissected intestines. Five different *P. vivax* isolates (biological replicates) were evaluated for each compound. Prevalence was calculated as defined in literature.^22^ Five different *P. vivax* isolates (biological replicates) were evaluated for each compound.

### DMFA from Drugs Added into the Blood

Blood samples were collected in heparinized tubes and centrifuged to remove the plasma. Erythrocytes were washed twice with RPMI-1640 and resuspended in inactivated human serum and then adjusted to a 40% hematocrit. Afterward, drugs at 10, 1, 0.5, and 0.1 μM were added in a 1 mL volume of this blood suspension and finally offered to the mosquitoes *via* membrane feeder. Between 100 to 120 females of *An. aquasalis* previously deprived of 10% sugar solution for 24 h were added in the cages *via* DMFA.^82^ All of the further procedures were performed as described above. Three different *P. vivax* isolates (biological replicates) were evaluated for each drug.

### Statistical Analysis

Normality was evaluated by the Shapiro-Wilk test. The comparison between groups was performed by ANOVA followed by Dunnett′s or Kruskal–Wallis followed by Dunn’s multiple-comparisons post-test. Mosquitoes and animal survival were analyzed using Log-rank (Mantel-Cox) test. Statistical significance was defined as *p* < 0.05. All analyses were conducted using GraphPad Prism 9 version 9.5.1 software (GraphPad Software Inc., San Diego, CA). Parts of Figures 1, 3, 5–7, and graphical abstract (ToC) were created in BioRender (www.biorender.com).

## Supplementary Material





## Notes

Complementary text of the Results section, NMR, EPR, and UV–vis spectra, and voltamogramms of the metal complexes (Tables S1–S3; Figures S1–S36), raw data of pharmacological results (Tables S3–S7 and Figures S37, S38) (PDF)
Molecular formula strings (CSV)

## ○

Camila Fabbri Universidade Federal do Amazonas, Faculdade de Ciências Farmacêuticas, Manaus, 69077-000, AM, Brazil

## Abbreviations Used

*An*: *Anopheles*
ATV: atovaquone
CC_50_: cytotoxic concentration required for 50% inhibition *in vitro*
CQ: chloroquine
DHA: dihydroartemisinin
DMFA: direct membrane feeding assay
DMSO: dimethyl sulfoxide
HepG2: hepatocellular carcinoma cells
IC_50_: inhibitory concentration required for 50% inhibition *in vitro*
ND: not determined
NMR: nuclear magnetic resonance
pRBCs: parasitized red blood cells
*P. berguei*: *Plasmodium berguei*
*P. falciparum*: *Plasmodium falciparum*
*P. vivax*: *Plasmodium vivax*
SAR: structure–activity relationship
SEM.: standard error of the median
SD.: standard deviation
