# Supplementary material for: Ruthenium Complexes of Atovaquone Acting on Multiple Stages of the Plasmodium Life Cycle
Source: J Med Chem. 2026 Apr 11;69(9):10083–103. doi: 10.1021/acs.jmedchem.5c02978 (PMC13181773; doi:10.1021/acs.jmedchem.5c02978)
Supplement: Supplementary file 1 [file jm5c02978_si_001.pdf]

Supporting information for

Ruthenium complexes of atovaquone acting on multiple stages of the *Plasmodium* life cycle

Camila Fabbri[1,2#,♠], Pedro Henrique S. Marcon[3#], Aline de Sousa Santiago[1#], Caroline Conceição Sousa[4], Helenita Costa Quadros[4], Larissa de Sena Lamar Nunes[2], Dione D. Maciel de Menezes[2], Rosa Amélia Gonçalves Santana[1], Silvia Cássia B. Justiniano[1], Sarah D'Alessandro[5], Nicoletta Basilico[6], Diogo R. M. Moreira[4,*], João Honorato de Araujo-Neto[3,*], Stefanie Costa Pinto Lopes[1,2,*]

[1] Fundação de Medicina Tropical Dr. Heitor Vieira Dourado (FT-HVD). Unidade de Pesquisa Clínica Carlos Borborema, Manaus, 69040-000, AM, Brazil.

[2] Instituto Leônidas e Maria Deane. Fiocruz Amazônia, 69057-070, Manaus, AM, Brazil.

[3] Departamento de Química Fundamental. Instituto de Química. Universidade de São Paulo (USP). São Paulo, 05508-000, São Paulo, SP, Brazil.

[4] Instituto Gonçalo Moniz. Fiocruz Bahia, 40296-710, Salvador, BA, Brazil.

[5] Dipartimento di Scienze Farmacologiche e Biomolecolari, Università degli Studi di Milano, 20133 Milan, Italy.

[6] Dipartimento di Scienze Biomediche, Chirurgiche e Odontoiatriche, Universitá degli Studi di Milano, 20133 Milan, Italy.

[#]Contributed equally.

*Corresponding authors: D.R.M.M. at diogo.magalhaes@fiocruz.br, J.H.A.N. at joaohonorato@usp.br, and S.C.P.L. at stefanie.lopes@fiocruz.br

♠ Present address: Universidade Federal do Amazonas, Faculdade de Ciências Farmacêuticas, Manaus, 69077-000, AM, Brazil.

Summary (table of content)

Entry	Description	Page number
	Complementary text of result section	S6
**Table S1**	Crystal data and refinement details for (**1-3)**	S12
**Table S2**	List of bond angles and lengths in the complexes' structures and the free atovaquone (ATV).	S13
**Figure S1**	Mass spectrometry spectra for complexes (**2, left**) and (**3, right**) alongside with their simulated designated peaks.	S13
**Figure S2**	Fourier-transform infrared spectroscopy (FTIR) spectrum of ATV with most relevant attributed signals.	S14
**Figure S3**	FTIR spectrum of complex (**3**) with most relevant attributed signals.	S14
**Figure S4**	FTIR spectrum of complex (**2**) with most relevant attributed signals.	S15
**Figure S5**	FTIR spectrum of complex (**1**) with most relevant attributed signals.	S15
**Figure S6**	Ultraviolet-Visible absorption spectra of complexes (**3, TATV**) (black line), next to the spectra of its precursor *cis,fac*-[RuCl_2(dmso-S)$_3$(dmso-O)] (green) and the free protonated (red) and deprotonated ATV (blue) forms of atovaquone, in DMSO (respective molar concentrations in parenthesis).	S16
**Figure S7**	Ultraviolet-Visible absorption spectra of complexes (**2, MATV**) (black), alongside with the spectra of its precursor *cis,fac*-[RuCl_2(dmso-S)$_3$(dmso-O)] (green) and the free protonated (red; ATVH) and deprotonated (blue; ATV) forms of atovaquone, in DMSO (respective molar concentrations in parenthesis).	S16
**Figure S8**	Ultraviolet-Visible absorption spectra of complexes (**1, AqATV**) (black) and the free protonated (red; ATVH) and deprotonated (blue; ATV) forms of atovaquone, in DMSO (respective molar concentrations in parenthesis).	S17
**Figure S9**	1D ^{1}H NMR spectrum of complex (**3**) and the free atovaquone (ATV) ligand ($CDCl_3$).	S17
**Figure S10**	1D ^{13}C NMR spectrum of complex (**3**) ($CDCl_3$).	S18
**Figure S11**	2D NMR ^{1}H-^{1}H COSY contour map obtained for complex (**3**) ($CDCl_3$).	S18
**Figure S12**	2D NMR ^{1}H-^{13}C HSQC correlation contour map obtained for complex (**3**) (red: CH_2; blue: CH/CH_3; $CDCl_3$).	S19
**Figure S13**	2D NMR ^{1}H-^{13}C HMBC correlation contour map obtained for complex (**3**) ($CDCl_3$).	S19
**Figure S14**	1D ^{1}H NMR spectrum of complex (**2**) and the free atovaquone ligand ($CDCl_3$).	S20
**Figure S15**	1D ^{13}C NMR spectrum of complex (**2**) ($CDCl_3$).	S20

**Figure S16**	2D NMR ^{1}H-^{1}H COSY contour map obtained for complex (**2**) ($CDCl_3$).	S21
**Figure S17**	2D NMR ^{1}H-^{13}C HSQC correlation contour map obtained for complex (**2**) (red: CH/CH_3; blue: CH_2; $CDCl_3$).	S21
**Figure S18**	2D NMR ^{1}H-^{13}C HMBC correlation contour map obtained for complex (**2**) ($CDCl_3$).	S22
**Figure S19**	$^{31}P\{^{1}H\}$ NMR spectrum of complex **(2**) in dichloromethane with D_2O capillary.	S22
**Figure S20**	Cyclic and differencial pulse voltamogramms of the free ligand ATV in dichloromethane, PTBA 0.1 M, Pt WE/CE and Ag/AgCl reference electrode.	S23
**Figure S21**	Cyclic and differencial pulse voltamogramms of complex (**1**) in dichloromethane, PTBA 0.1 M, Pt WE/CE and Ag/AgCl reference electrode.	S23
**Figure S22**	Cyclic and differencial pulse voltamogramms of complex (**2**) in dichloromethane, PTBA 0.1 M, Pt WE/CE and Ag/AgCl reference electrode.	S24
**Figure S23**	Cyclic and differencial pulse voltamogramms of complex (**3**) in dichloromethane, PTBA0.1 M, Pt WE/CE and Ag/AgCl reference electrode.	S24
**Figure S24**	(a) Experimental electron paramagnetic resonance (EPR) spectra of complex (**1**) recorded at 77 K in the solid state and in CH_2Cl_2 solution. (b) Deconvolution and simulation of the EPR spectra in solution, showing the contribution of two components to the overall signal.	S25
**Figure S25**	Hirshfeld surfaces of complexes (**1-3**) mapped with d_{norm} function and the respective atom trypes responsible for the most relevant contacts (top). Quantitative two-dimensional FingerPrint Plots (middle) and the graph of the percentage fractions (bottom) of individual atom contacts present on the Hirshfeld surface of the complexes. The color of the regions marked on the FP plots match the color of the bars in respect to the following reciprocal contacts: H···H in dark blue; H···C in red; H···Cl in green; C···C in orange; H···O in yellow; Cl···O in pink and others and black.	S26
**Figure S26**	Full interaction maps for complexes (**3**, panel A); (**2**, panel B) and (**1**, panel C). The calculated clouds surrounding the complexes' environment are color coded: uncharged NH nitrogen probe in blue; RNH_3 nitrogen probe in purple; carbonyl oxygen probe in red; aromatic CH carbon probe in yellow.	S27
**Figure S27**	Torsion angles of the atovaquone's chlorophenyl ring (green) in respect to the naphthoquinone rings (red) for complexes (**1-3**). Hydrogen atoms were omitted for clarity.	S28
**Figure S28**	Stability study by Uv-Vis absorption of complex (**3**) in DMSO.	S29
**Figure S29**	Stability study by Uv-Vis absorption of complex (**2**) in DMSO.	S29
**Figure S30**	Stability study by Uv-Vis absorption of complex (**1**) in DMSO.	S30
**Figure S31**	Stability study by Uv-Vis absorption of complex (**3**) in 50% DMSO/H_2O.	S30

**Figure S32**	Stability study by Uv-Vis absorption of complex (**2**) in 50% DMSO/H_2O.	S31
**Figure S33**	Stability study by Uv-Vis absorption of complex (**1**) in 50% DMSO/H_2O.	S31
**Figure S34**	^1H NMR spectra employed for monitoring the stability of complex (**2**) in solutions containing DMSO-d_6 and D_2O. The two lowest spectra display the chloride abstraction from the reaction upon adding $AgClO_4$. The initial chloride containing complex is depicted with a green star, and the speciated complexes acqua-**2** and dmso-**2** with a red circle and yellow heart, respectively.	S32
**Figure S35**	^1H NMR spectra employed for monitoring the stability of complex (**3)** in solutions containing DMSO-d_6 and D_2O. The two lowest spectra display the chloride abstraction from the reaction upon adding $AgClO_4$. The initial chloride containing complex is depicted with a green star, and the speciated complexes acqua-**3** and dmso-**3** with a red circle and yellow heart, respectively.	S33
**Figure S36**	Fluorescence spectra of HSA solution (2.5 μM) in Tris–HCl buffer (0.1 M NaCl, pH 7.4) in the absence and presence of different concentrations of complexes. Panel A, complex (**3**); panel B, complex (**2;**) panel C, complex (**1**). Conditions: a = 0; b= 2.5 μM; c = 5.0 μM; d = 7.5 μM; e = 10.0 μM; f = 12.5 μM; g = 15.0 μM; h = 17.5 μM and i = 20.0 μM at 298 K. Inset: Stern–Volmer plots for the quenching of HSA fluorescence by complexes, 298 and 310 K.	S34
**Table S3**	Stern–Volmer quenching constant (K_{sv}, L mol^{-1}); biomolecular quenching rate constant (K_q, L mol^{-1} s^{-1}); binding constant (K_b, L mol^{-1}); number of binding sites (*n*), ΔG° (KJ mol^{-1}), ΔH° (KJ mol^{-1}) and ΔS° (J mol^{-1} K) values for the complex–HSA system at different temperatures.	S35
**Table S4**	Cell toxicity for HepG2 and J774 cells, association constant (log *K*) for hemin, β-hematin inhibitory activity (BHIA) and selectivity indexes (S.I) for Atovaquone and the metal complexes (**1-3**).	S35
**Table S5**	ATV and its Ru complexes (**2 and 3**) inhibit the growth of asexual blood stages of *P. falciparum*. Growth was assessed by SYBR green I method. Goodness-of-fit of non-linear model was assessed using the coefficient of determination (R^2). Values associated to Figure 3B.	S36
**Table S6**	Activity of metal complexes in inhibiting the growth of asexual blood stages of *P. falciparum*.	S36
**Table S7**	Direct membrane feeding assay (DMFA) from drug-coated surfaces which were exposure to the tarsal area or drugs added into the bloodmeal of mosquitoes.	S37
**Table S8**	Direct membrane feeding assay (DMFA) from drugs added into the bloodmeal of *An. aquasalis*.	S37
**Figure S37**	Panel A) Quantification of parasite stages in 3D7 strain of *P. falciparum* at 24 h. At least five fields from each slide were counted from giemsa staining. Values are the median. Panel B) Quantification of parasites (iRBCs) with visible hemozoin crystals determined by polarized light microscopy in 3D7 strain of *P. falciparum* at 48 or 72 h h. Eight micrographs from each group were counted. Values are the mean and S.D. Data associated with Figure 4.	S38

**Figure S38**	Survival of *An. darlingi* from drug-coated surfaces exposure to the tarsal area. Black lines show control, where mosquitoes were solely exposed with compound diluent. Panels A-D are from one experiment, while panels E-H are from another independent experiment. Panels A and E: Atovaquone at 200 µmol/m^2; Panels B and F: complex (**3**) at 200 µmol/m^2; Panels C and G: complex (**3**) at 20 µmol/m^2; Panels D and H: complex (**3**) at 2 µmol/m^2. Indicated *p* values were calculated using log-rank (Mantel-Cox test).	S39
	References	S40

Complementary text for result section

Complexes *cis*-[$RuCl_2$(ATV)(dppb)] (**1**), *cis*-[RuCl(dmso-S)$_2$(ATV)(PPh_3)] (**2**) and *fac*-[RuCl(dmso-S)$_3$(ATV)] (**3**) were synthesized by reacting equimolar amounts of ATV with Ru(II) and Ru(III) precursors under inert atmosphere at room temperature, affording neutral and air-stable solids.

The use of triethylamine and a non-coordinating solvent during the preparation of *cis*-[RuCl(dmso-S)$_2$(ATV)(PPh_3)] (**2)** promote an efficient deprotonation of ATV and this condition favored its binding to the ruthenium center; this complex underwent additional purification in NaCl/ethanol solution, whereas complexes (**1)** and (**3**) were directly obtained from methanol without further steps.

All compounds were soluble in DMSO, methanol, ethanol, dichloromethane, and chloroform, but insoluble in water. Elemental analyses confirmed their proposed formulations (purity > 95 %). The design strategy aimed to enhance the therapeutic potential of ruthenium by employing ATV as an *O,O*-bidentate ligand, since previous studies demonstrated that such coordination favors target recognition while maintaining strong metal binding.

The recorded molar conductivity values confirmed their 1:1 electrolyte nature in DMSO. The structural assignments were supported by elemental analysis and full spectroscopic characterization. Importantly, the crystal structures of the three complexes were determined by single-crystal X-ray diffraction, providing definitive confirmation of their molecular architectures.

One of the structural characterization techniques employed in this work was electron-spray ionization mass spectrometry in positive mode (ESI(+)-MS) to determine the mass to charge ratio of the complexes. Indeed, complexes (**2)** and (**3)** were presented as their protonated species $[M+H]^+$ with monoisotopic *m*/*z* values of 921.0960 and 737.0155, respectively. All the assigned values and the spectrogram peak pattern match simulated data and are found in **Figure S1**. It was not possible to obtain a clear monoisotopic pattern of the intact complex (**1)**, and the interpretation of the residual fragmentations was not attempted.

The infrared vibrational spectra (IR) recorded for the complexes, as well as for the free protonated atovaquone, led to insights about the molecular changes that occur upon coordination to the metallic centers of Ru(II) and Ru(III). IR spectrum of ATV presents a sharp band in 3370 cm^{-1} assigned as $\nu(O_1\text{-}H)$, engaged in

hydrogen bonding (Pavia, 2015), which in turn becomes absent in the spectra of (**1–3**), suggesting the deprotonated state of the chelate ligand bonded to the metallic centers. Stretching of C-H can also be seen in the range from 2964 to 2855 cm^{-1} for all species analyzed. Two bands in ATV were assigned as the carbonyl stretching bands ν(C_2=O) and ν(C_9=O) at 1655 and 1633 cm^{-1}. Due to coordination via C_2=O carbonyl, it is possible to observe the shift to lower wavenumbers as 1570 cm^{-1} (**1**), 1561 cm^{-1} (**2**) and 1555 cm^{-1} (**3**), in accordance with similar ruthenium complexes with naphthoquinone ligands (Oliveira, 2021).

The bond C_9=O, although not directly involved in coordination, shows modest shift to lower wavenumber values, 1618 cm^{-1} (**1**), 1615 cm^{-1} (**2**), 1609 cm^{-1} (**3**), because of the electron cloud reorganization and the previously unaccounted resonance from the deprotonated O_1 electron, a phenomenon not observed on the free ligand IR spectrum. Finally, one feature commonly found in the spectra of **2** and **3** is the stretching vibration ν(S=O) at 1089 cm^{-1} and 1095 cm^{-1} respectively, since dimethyl sulfoxide ligands are present in their molecular structures (Gaur, 2012), in turn this is absent in ATV and (**1**).

The UV-Vis spectrum of ATV was recorded in DMSO, before and after deprotonation with a methanolic solution of NaOH. Prior to the addition of the base, the yellow atovaquone solution presented two strong absorption bands at 332 nm and 393 mn. When deprotonated, a significant bathochromic shift occurs in the form of a single, strong and broad absorption band at λ = 519 nm, leaving the solution with a strong red color. As for the complexes, in comparison to the deprotonated ATV ligand, it is observable a small bathochromic shift at λ_{max} of 555 nm (**1**), 594 nm (**2**), and 577 nm (**3**). We assigned those values to metal-to-ligand charge transfer (MLCT) bands, since the majority of ligands are π-acceptors with low energy π* orbitals. In addition, the formation of new bands indicates an expected behaviour regarding the formation of new species due to newly rearranged molecular orbitals. The spectra containing all discussed absorptive species and their metallic precursors are depicted in **Figure S2-S4**.

Nuclear magnetic resonance spectroscopy analyses were carried out for both diamagnetic Ru(II) complexes (**2**) and (**3**). 1D ^{1}H, ^{13}C and 2D ^{1}H-^{1}H COSY, ^{1}H-^{13}C HSQC and ^{1}H-^{13}C HMBC NMR spectra contain some description of the coordination mode of ATV and the chemical environment of the coordination compounds (**Figures S9-S19**). Both ^{1}H NMR spectra can be divided into three major regions concerning groups of δ values (a) for the main ligand's aromatic protons, (b) the dmso-*S* methyl protons and (c) the remaining H atoms of the cyclohexyl moiety of ATV. The first set of peaks can be found in the range of 8.03 – 7.15 for both

(**2)** and (**3**), and were attributed to the ligand aromatic hydrogen atoms H_4 through H_7 and $H_{16,17,19,20}$. In comparison to the free ligand, it is noted a larger chemical shift for the first group of aromatic H atoms as a consequence of coordination and, notably, such observed δ values shift upfield. In addition, spectrum of complex (**2**) contains signals from the triphenylphosphine rings, namely the *ortho*, *meta* and *para* H atoms.

We highlight the absence of the singlet observed in ATV spectrum at 7.50 ppm in the two complexes' spectra, which is assigned as the enolic -O_1H hydrogen atom, suggesting and corroborating what was previously noted by the FTIR analysis that the ligand binds to the ruthenium centers in a deprotonated fashion carrying one negative charge. The second set of peaks concerns the singlets in the high field region originated from the bonded dmso-S methyl groups. Complex **(3)** showed six singlets, each from one -CH_3, ranging from δ 3.73 – 3.20 ppm. In turn, complex (**2**) presented four singlets in the region from 3.62 ppm to 2.60 ppm, the number of such signals and the amount of hydrogen atoms they integrate to agree with the quantity dmso-*S* bonded to the central Ru(II) as shown in the proposed structures. The third set of peaks concern the region containing the chemical shifts of the cyclohexyl group of ATV, ranging from δ 3.26 to 1.50 ppm and, virtually, remain on the same values of δ as the free ligand, except for H_{11} shifting downfield.

$^{31}P\{^1H\}$ NMR was used to characterize the chemical shift of the phosphorous atom present in the structure of (**2**), which was a singlet at a δ 41.17 ppm (in CH_2Cl_2/D_2O capillary). 2D NMR experiments were also fundamental in assigning the carbon atoms in the structures of the complexes. The most deshielded peaks were attributed as the quaternary carbon atoms directly bonded to oxygen and chlorine atoms, such as C_1, C_2, C_9, and C_{18}. Later, in the aromatic region of the spectra, all phosphine (*s+o+m+p*) and naphtoquinone ($C_{3\text{-}8}$, $C_{15\text{-}20}$, C_{10}) peaks. Towards the low field region, the dmso-S methyl carbon atoms tend to be grouped between δ 50-40 ppm, with **2** and **3** clearly showing six and four peaks, respectively. In that same space lies the chemical shift of the cyclohexyl C_{14}H and down to δ 26 ppm we assigned the group's remaining -CH_2. Overall, the NMR spectra closely resemble each other, despite the clear differences such as the presence of the PPh_3 ligand, and in themselves correspond to the expected and proposed molecular structures.

The electrochemical behavior of the complexes studied in this work were based on the oxidation potential shift Ru(II)/Ru(III) after the substitution of one chloride (good σ-π donor) and water (σ donor) ligands by the bidentate naphthoquinone in (**1**) and the substitution of one chloride (good σ-π donor) plus one dmso-S (good π acceptor) ligands by the same naphthoquinone in **(2)** and **(3)**. It was possible to observe a common

feature between the latter: the irreversible reduction of the process involving the metallic center. Complex (**3)** cyclic voltammogram closely resembles that of its precursor, evidencing the capacity of the remaining dmso-S coligands in stabilizing the complex electrochemically. The irreversibility process in **2**, happened after the binding of ATV to the precursor, suggesting a protective action against the Ru(II)/Ru(III) oxidation by the ligand. Differently, complex (**1)** showed a quasi-reversible one-electron redox process in the -0.2 to 0.4 V region. The value of $E_{\frac{1}{2}}$ for that Ru(III)/Ru(II) couple was 0.14 V, while E_p = 125.9 mV and the peak current ratio of 0.549. Nevertheless, all complexes demonstrated the irreversible oxidation process that occurs on the ligand at around 1.5 V. All voltammograms regarding the precursors, obtained complexes and the free ATV are represented in **Figure S20-S23**.

The electron paramagnetic resonance (EPR) spectra of complex (**1)** were recorded at 77 K, both in the solid state and in dichloromethane solution. In the solid state, the spectrum displays a single rhombic component, whose *g* anisotropy is fully consistent with the distorted octahedral geometry determined from single-crystal X-ray diffraction. In frozen solution, however, two components were required: the dominant one (97 %) corresponding to the intact complex, and a minor contribution (3 %) attributed to partial chloride substitution. This minor species is likely formed due to the strong trans influence of the phosphine ligand, which weakens the Ru–Cl bond, enabling limited coordination of solvent or water molecules.

The crystallographic studies were essential to determine the molecular structures and intra/intermolecular interactions of complexes (**1–3)**, using single-crystal X-ray diffraction from dark block-shaped crystals. Complex 3 crystallized in the monoclinic system ($P2_1/c$) with two molecules in the asymmetric unit, one coordination complex and one chloroform solvent. Complex **2** crystallized in the orthorhombic system (Pbca), containing one molecule and a dichloromethane solvent, while complex **1** crystallized in the monoclinic system ($P2_1/n$) with a single molecule in the asymmetric unit. All structures feature a distorted octahedral Ru(II) coordination environment, as illustrated in **Figure 2(b).** Across all three complexes, atovaquone coordinates as a deprotonated O,O-bidentate ligand through the enolate (O_1^-) and carbonyl (O_2) groups, forming a five-membered chelate with bite angles below 90° (**Table S2**). In complex (**3)**, the remaining sites are occupied by three *fac*-arranged dmso-S ligands and one chlorido coligand, yielding a neutral Ru(II) species. Complex (**2)** contains two *cis* dmso-S ligands and one triphenylphosphine trans to the chlorido, whereas in complex (**1)**

neutrality is maintained by two *cis* chlorides and a dppb ligand, with P atoms trans to Cl_1 and O_2. Comparison with free ATV (Nayak, 2013) shows systematic bond rearrangements upon coordination: $C_1–O_1$ shortens, $C_2=O_2$ elongates, while $C_9=O_3$ remains essentially unchanged, consistent with FTIR data. The Ru–O distances follow the expected trend of shorter Ru–O_1 and longer Ru–O_2, reflecting π* back-donation into the coordinated carbonyl. This effect is accentuated in complex **1**, where the harder Ru(III) center interacts strongly with the enolate $O1^-$, shortening Ru–O1 and lengthening Ru–O2, in line with Pearson's HSAB principle. These observations are consistent with previously reported ruthenium–dmso complexes with related ligands (Prajapati, 2010).

They share one common feature, the atovaquone ligand coordinates as a bidentate and deprotonated chelate by the O_2 carbonyl and O_1^- enolate atoms, generating a new five membered ring with bite angles smaller than 90°, as shown in **Table S2**. Three other coordination sites of **3** are occupied by sulfur-bonded dimethyl sulfoxide coligands in a *fac* geometry and the coordination sphere is completed with a chlorido ligand (Cl_1), and together with ATV, balances out the central bivalent charge of this neutral complex. As for complex (**2**), only two dmso-S ligands are found bonded to Ru(II), here in a *cis* geometry, while a triphenylphosphine ligand sits *trans* to the negatively charged chlorido ligand (P_1-Cl_1). In turn, neutrality in (**1**) is achieved by the combination of ATV and two Cl^- (in a *cis* geometry), since the central ruthenium is a trivalent cation. The remaining two coordination sites are thus occupied by one dppb ligand having its phosphorus atoms *trans* to one chlorido (P_1-Cl_1) and the ketone oxygen atom (P_2-O_2). Some relevant bond lengths were compared to a previously published atovaquone crystal structure (Nayak, 2013) and depicted in **Table S1**. The enolate C_1-O_1 bond gets shorter after coordination, while the $C_2=O_2$ carbonyl bond elongates, mostly because it accepts electronic density from the metallic center in its π* orbitals, across all complexes. On average, $C_9=O_3$ didn't show significant bond length difference from the free ligand, once again corroborating FTIR data. The hardness/softness of the central metals play an important role on the length of Ru_1-O_1 and Ru_1-O_2. The three complexes showed the same tendency of shorter Ru_1-O_1 bonds and longer Ru_1-O_2 bonds, which was expected since $C_2=O_2$ accepts electronic density from relative electron rich Ru(II) into its π* orbitals, such behavior matches previously published ruthenium-dmso complexes with similar ligands (Prajapati, 2010). However, the difference between those bond lengths is larger for (**1**) than (**2**) and (**3**), since the harder Ru(III) acid and the hard base enolate O_1^-'s orbitals show a larger

overlap, shortening Ru_1-O_1 in the expense of enlarging Ru_1-O_2 to be able to accommodate the new five-membered ring, highlighting Pearson's hard/soft acid and base relationship towards the balance in bond lengths.

To rationalize the biological behavior of the complexes, it is essential to examine their intermolecular interactions. Hirshfeld surface (HS) analysis provides a qualitative and quantitative view of atomic contacts within the crystal structures (Spackman, 2009). Among the different mapping functions, d_{norm} is particularly useful, as it combines the normalized distances of the nearest atoms inside (di) and outside (de) the surface with their van der Waals radii, highlighting close contacts as red spots, and longer contacts as white or blue regions. In **Figure 2 (panel C) and S25**, the red-spotted areas on complexes (**2**) and (**3**) arise mainly from non-classical C–H···A hydrogen bonds with oxygen and chlorine atoms as acceptors. Examples include C_{17}–$H_{17}\cdots O_3$ (3.252(2) Å/159°) and C_{26}–$H_{26A}\cdots O_4$ (3.264(2) Å/144°) in **2**, and C_{24}–$H_{24B}\cdots O_4$ (3.261(3) Å/142°) and C_{25}–$H_{25B}\cdots O_5$ (3.216(3) Å/123°) in (**3**). Additional contacts involve solvent molecules in the lattice, such as C–H···Cl and C–H···O. In contrast, complex (**1**) exhibit fewer of these interactions, consistent with its bulkier dppb ligand, which increases molecular separation within the crystal. Quantitatively, H···H contacts dominate the surfaces (43.6% for **1**, 55% for **2** and 54.3% for **3**), followed by H···C and H···Cl contributions, while H···O, Cl···O, and $\pi\cdots\pi$ contacts were minor but detectable. These findings indicate that oxygen and chlorine atoms play a key role in crystal packing, either by non-classical hydrogen bonding or by concentrating electron density at the periphery of the molecules.

Further insights into structure–function relationships were obtained through full interaction maps (FIMs) using Mercury (Macrae, 2006). Blue and purple regions correspond to NH and R–NH_3 probes (hydrogen-bond donors), red areas indicate carbonyl oxygen acceptors, and yellow regions highlight hydrophobic CH interactions. Across all complexes (**Figure S26**), the C_9=O_3 group consistently appears near blue/purple regions, confirming its ability to accept H-bonds. A clear trend emerges with increasing DMSO ligands: complexes **1** < **2** < **3** show progressively more hydrophilic donor regions, while the yellow hydrophobic regions decrease with the loss of phosphine ligands. This suggests that complex **3** engages predominantly in polar interactions, whereas **1** and **2** retain stronger lipophilic character due to their phosphine content.

The orientation of the *para*-substituted chlorophenyl ring of atovaquone, known to be crucial for biological activity (Baggish, 2002; Nayak, 2013), was examined through crystallographic dihedral angles. The torsion between the Arom–$C_{15–20}$–Cl plane and the naphthoquinone C_1–C_{10} plane was significantly larger in (**3**)

(27.23(11)°) than in **2** (12.21(6)°) or **1** (13.7(2)°), indicating greater rotational freedom of this ring in (**3**). This conformational feature is likely influenced by steric effects from phosphine ligands, which may alter the way the chlorine atom fits into the enzyme binding pocket and, consequently, impact biological activity (**Figure S27**).

Table S1. Crystal data and refinement details for (**1–3).**

Complex	**(1)**	**(2)**	**(3)**
CCDC number	2489676	2489677	2489675
Empirical formula	$C_{50}H_{46}Cl_3O_3P_2Ru$	$C_{45}H_{47}Cl_4O_5PRuS_2$	$C_{29}H_{37}Cl_5O_6RuS_3$
Formula weight /g mol^{-1}	964.291	1.005.853	856.139
Temperature /K	293(2)	100.15	100.15
Crystal system	monoclinic	orthorhombic	monoclinic
Space group	P21/n	Pbca	P21/c
a/Å	13.3253(3)	21.0383(1)	17.2721(2)
b/Å	10.3422(2)	16.2173(1)	23.5966(3)
c/Å	31.8007(7)	25.4380(1)	8.6314(1)
α/°	90	90	90
β/°	91.135(2)	90	97.023(1)
γ/°	90	90	90
Volume/Å^3	4381.69(16)	8679.05(8)	3491.44(7)
Z	4	8	4
ρ_{calc}g/cm^3	1.462	1.540	1.629
μ/mm^{-1}	0.657	6.814	9.178
F(000)	1978.5	4158.8	1760.1
Crystal size/mm^3	0.481 × 0.384 × 0.254	0.163 × 0.077 × 0.023	0.105 × 0.048 × 0.012
Radiation	Mo Kα (λ = 0.71073)	Cu Kα (λ = 1.54184)	Cu Kα (λ = 1.54184)
2Θ range for data collection/°	4.98 to 52	6.94 to 159.32	5.16 to 158.68
Index ranges	-21 ≤ h ≤ 20, -15 ≤ k ≤ 16, -50 ≤ l ≤ 49	-26 ≤ h ≤ 26, -20 ≤ k ≤ 19, -31 ≤ l ≤ 32	-21 ≤ h ≤ 22, -30 ≤ k ≤ 29, -10 ≤ l ≤ 8
Reflections collected	140598	121647	38932
Independent reflections	8618 [R_{int} = 0.0494, R_{sigma} = 0.0349]	9395 [R_{int} = 0.0495, R_{sigma} = 0.0229]	7474 [R_{int} = 0.0514, R_{sigma} = 0.0384]
Data/restraints/parameters	8618/0/584	9395/0/552	7474/0/428
Goodness-of-fit on *F*^{2}	1.107	1.038	1.010
Final *R* indexes [*I* ≥ 2σ(*I*)]	R1 = 0.0470, wR2 = 0.0923	R1 = 0.0237, wR2 = 0.0602	R1 = 0.0339, wR2 = 0.0876
Final *R* indexes [all data]	R1 = 0.0586, wR2 = 0.1037	R1 = 0.0250, wR2 = 0.0610	R1 = 0.0390, wR2 = 0.0906
Largest diff. peak/hole/e Å^{-3}	1.14/-0.79	0.44/-0.54	0.80/-0.48

Table S2. List of bond angles and lengths in the complexes' structures and the free atovaquone (ATV).

	Angles (°)				Bond lengths (Å)				
					C_1-O_1	C_2=O_2	C_9=O_3	Ru_1-O_1	Ru_1-O_2
**3**	**O_1-Ru_1-O_2**	**S_1-Ru_1-O_2**	**S_2-Ru_1-O_1**	**S_3-Ru_1-Cl_1**					
	77.53(7)	168.80(5)	172.24(5)	173.66(2)	1.307(3)	1.250(3)	1.233(3)	2.0894(17)	2.1253(17)
**2**	**O_1-Ru_1-O_2**	**S_1-Ru_1-O_2**	**S_2-Ru_1-O_1**	**P_1-Ru_1-Cl_1**	1.300(2)	1.250(2)	1.231(2)	2.0915(11)	2.1094(11)
	77.60(4)	170.00(3)	171.10(3)	167.405(14)					
**1**	**O_1-Ru_1-O_2**	**P_2-Ru_1-O_2**	**P_1-Ru_1-Cl_1**	**P_1-Ru_1-P_2**	1.311(4)	1.240(4)	1.222(5)	2.006(2)	2.160(3)
	78.14(10)	172.52(7)	175.21(4)	91.79(4)					
**ATV**	-	-	-	-	1.353(3)	1.224(3)	1.226(3)	-	-

Figure S1. Mass spectrometry spectra for complexes (**2, left**) and (**3, right**) alongside with their simulated designated peaks.

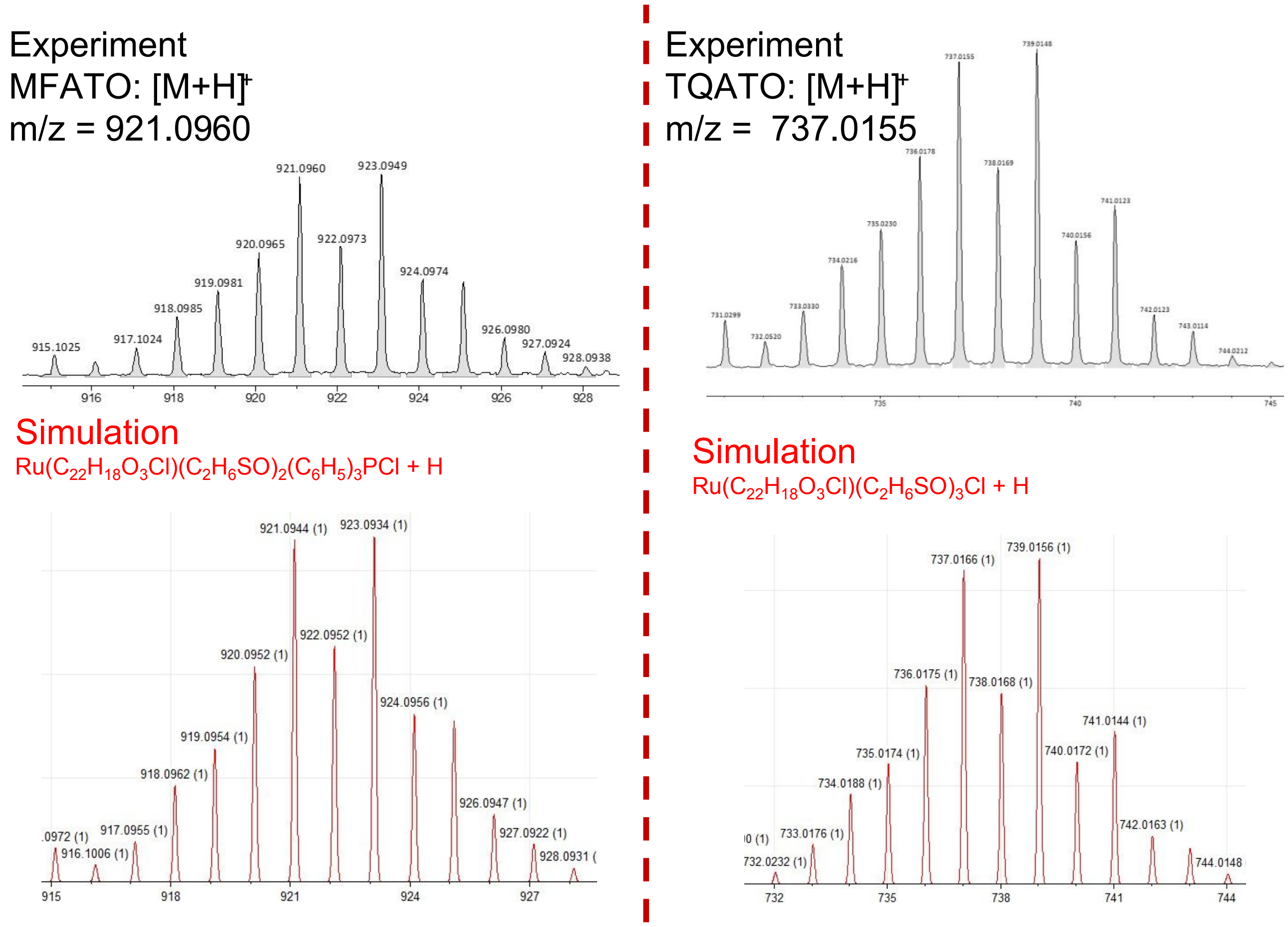

Figure S2. Fourier-transform infrared spectroscopy (FTIR) spectrum of ATV, highlighting the most relevant assigned signals.

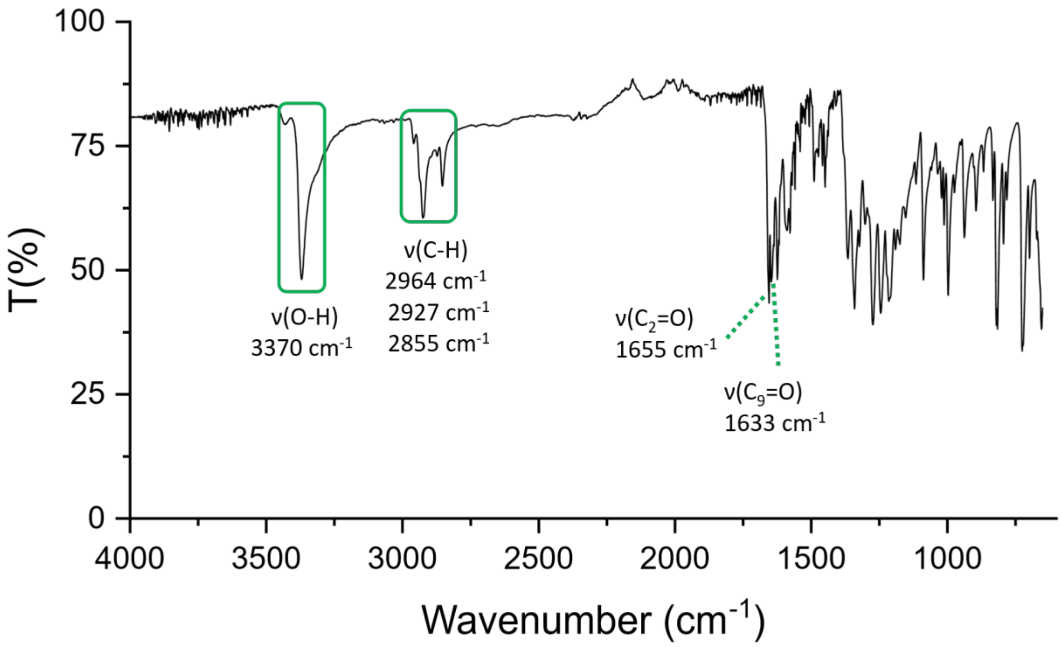

Figure S3. FTIR spectrum of complex (**3)** highlighting the most relevant assigned signals.

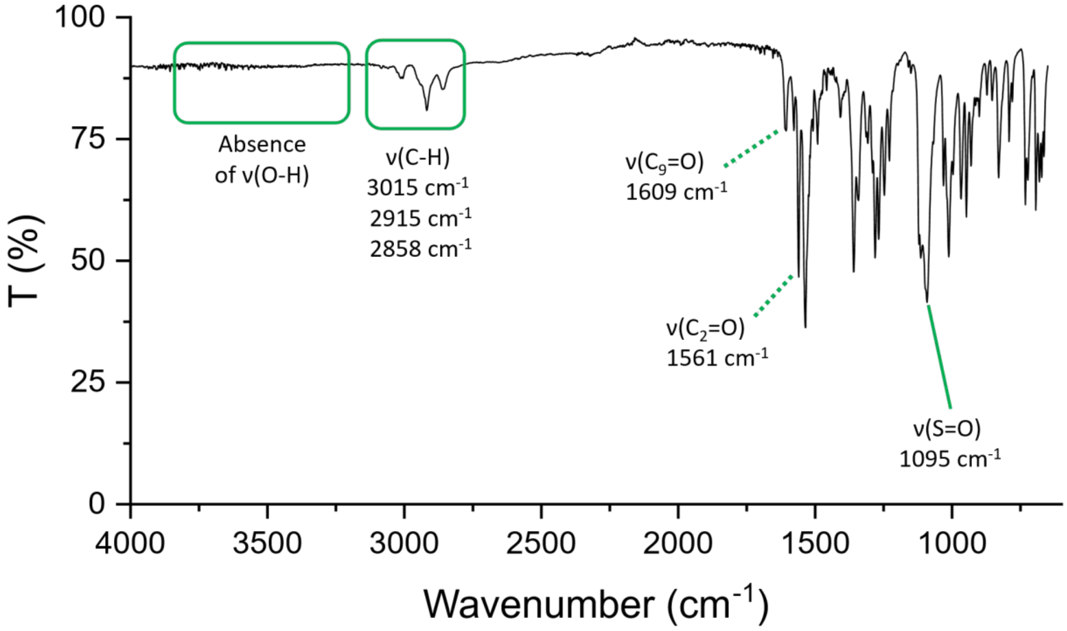

Figure S4. FTIR spectrum of complex (**2**) highlighting the most relevant assigned signals.

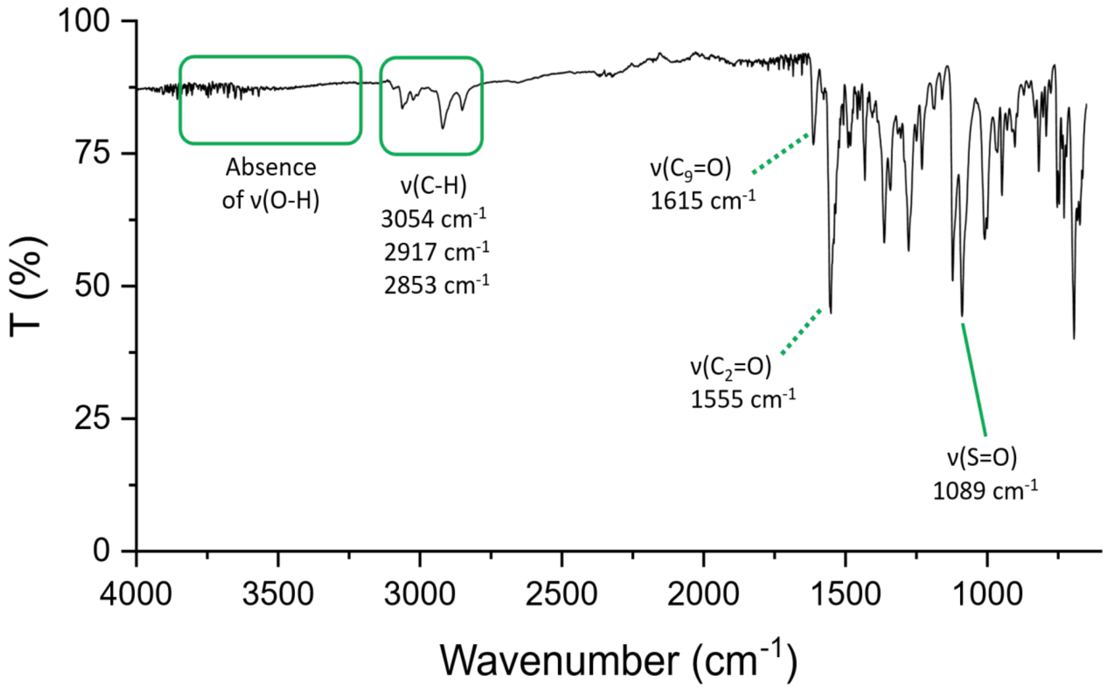

Figure S5. FTIR spectrum of complex (**1**) highlighting the most relevant assigned signals.

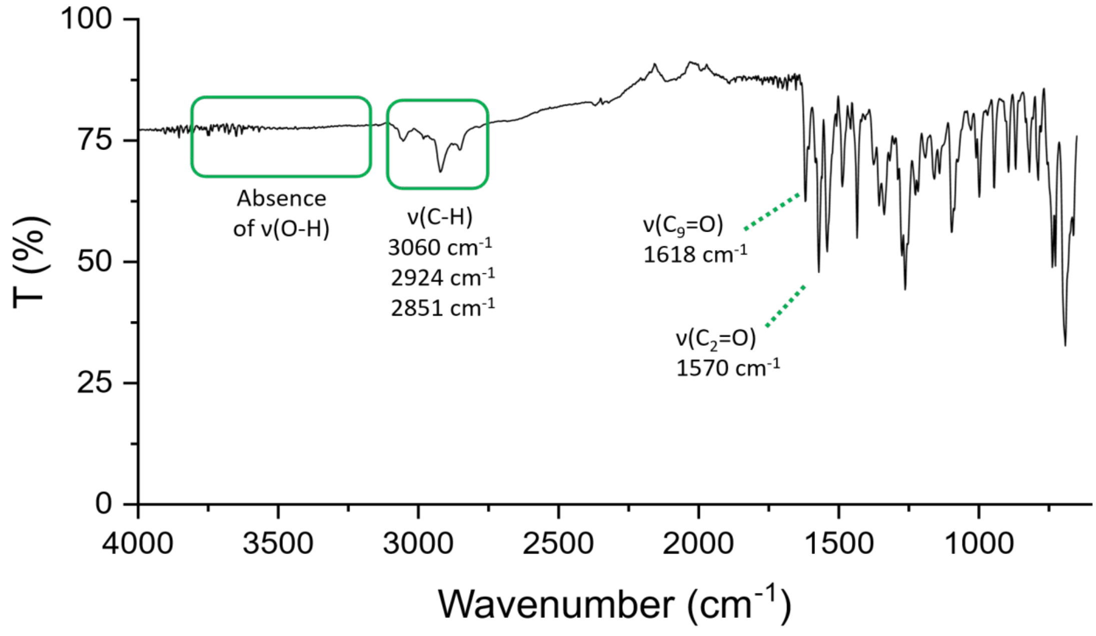

Figure S6. Ultraviolet-Visible absorption spectra of complexes (**3, TATV**) (black line), next to the spectra of its precursor *cis,fac*-[$RuCl_2$(dmso-S)$_3$(dmso-O)] (PREC) (in green) and the free protonated (red) and deprotonated (blue) forms of atovaquone (ATV), in DMSO (respective molar concentrations in parenthesis).

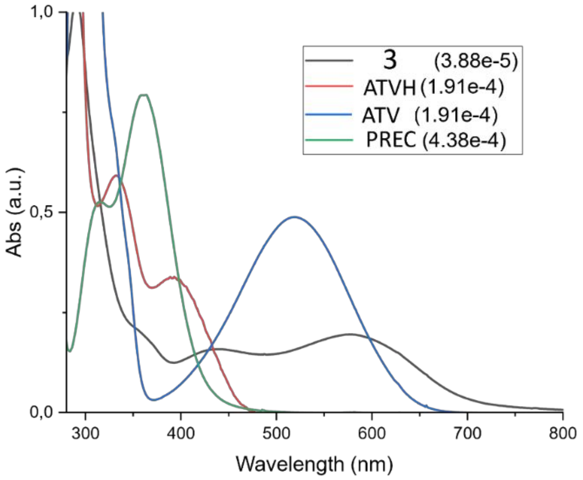

Figure S7. Ultraviolet-Visible absorption spectra of complexes (**2, MATV**) (black), alongside with the spectra of its precursor *cis,fac*-[$RuCl_2$(dmso-S)$_3$(dmso-O)] (green) and the free protonated (red; ATVH) and deprotonated (blue; ATV) forms of atovaquone, in DMSO (respective molar concentrations in parenthesis).

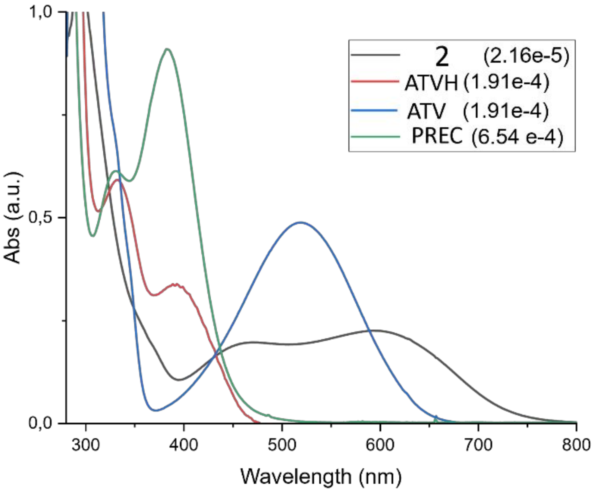

Figure S8. Ultraviolet-Visible absorption spectra of complexes (**1, AqATV**) (black) and the free protonated (red; ATVH) and deprotonated (blue; ATV) forms of atovaquone, in DMSO (respective molar concentrations in parenthesis).

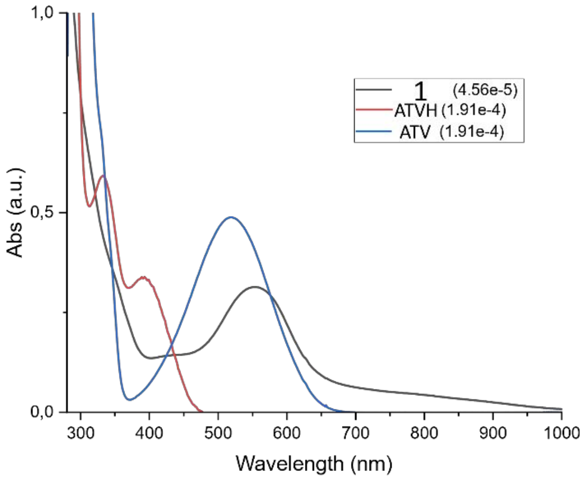

Figure S9. 1D ^{1}H NMR spectrum of complex (**3**) and the free atovaquone (ATV) ligand ($CDCl_3$).

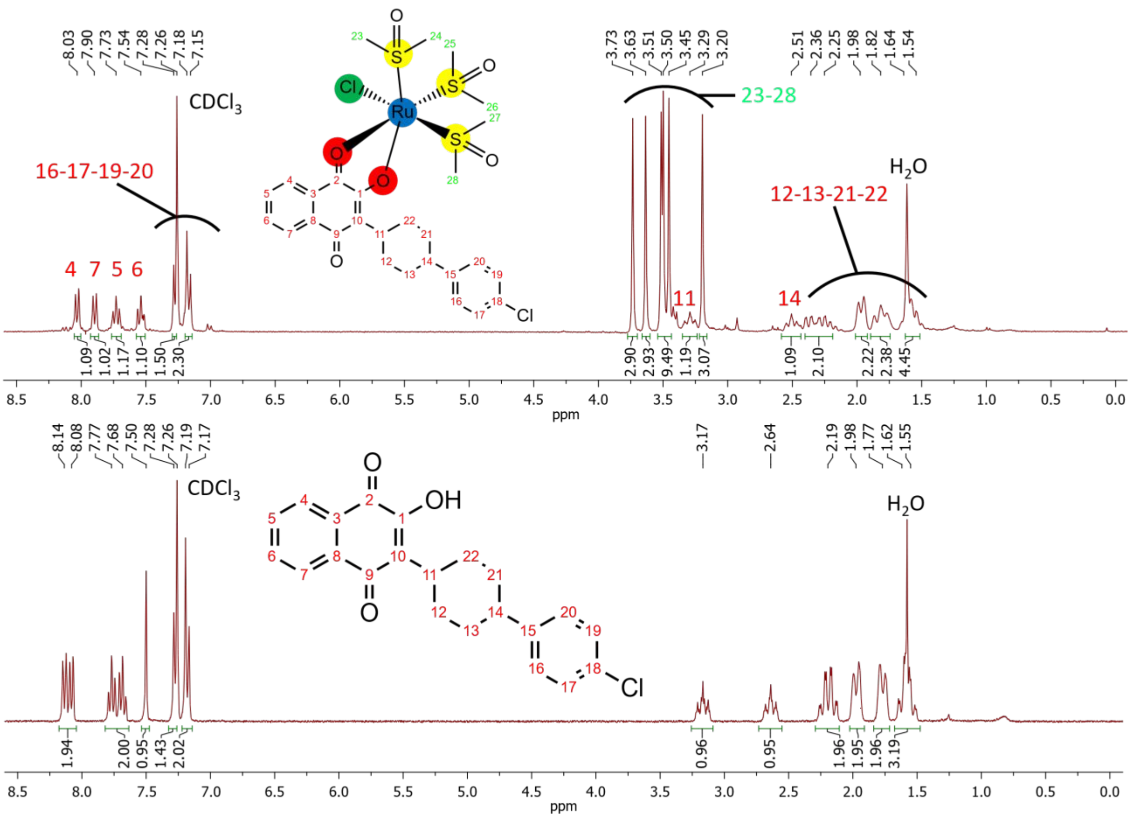

Figure S10. 1D ^{13}C NMR spectrum of complex (**3**) ($CDCl_3$).

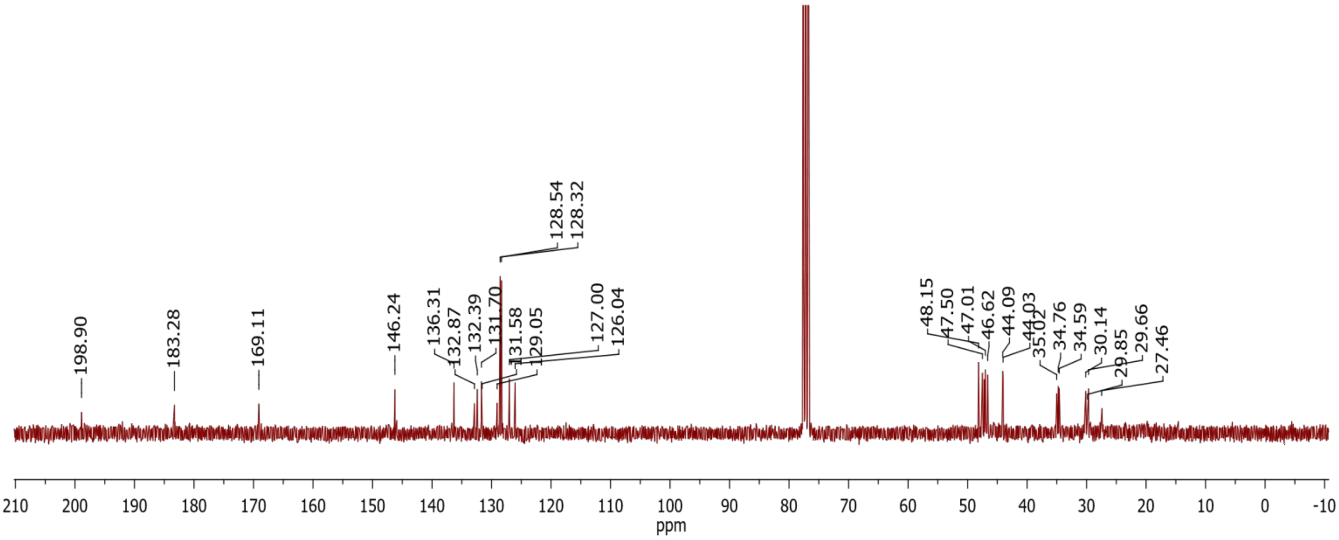

Figure S11. 2D NMR ^{1}H-^{1}H COSY contour map obtained for complex (**3**) ($CDCl_3$).

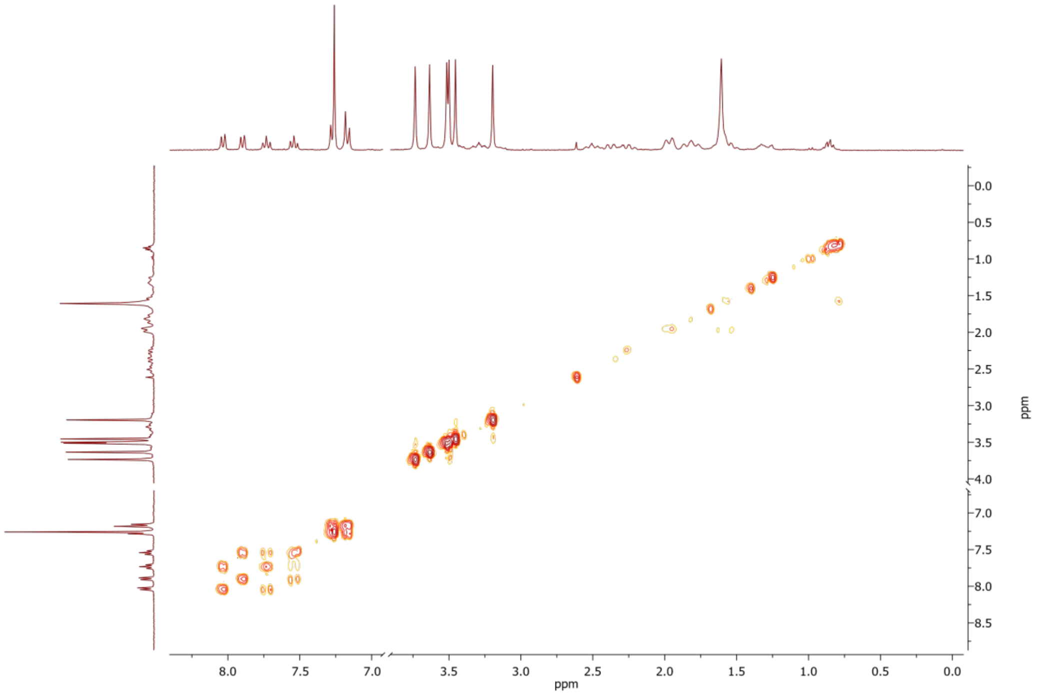

Figure S12. 2D NMR ^1H-^{13}C HSQC correlation contour map obtained for complex (**3**) (red: CH_2; blue: CH/CH_3; $CDCl_3$).

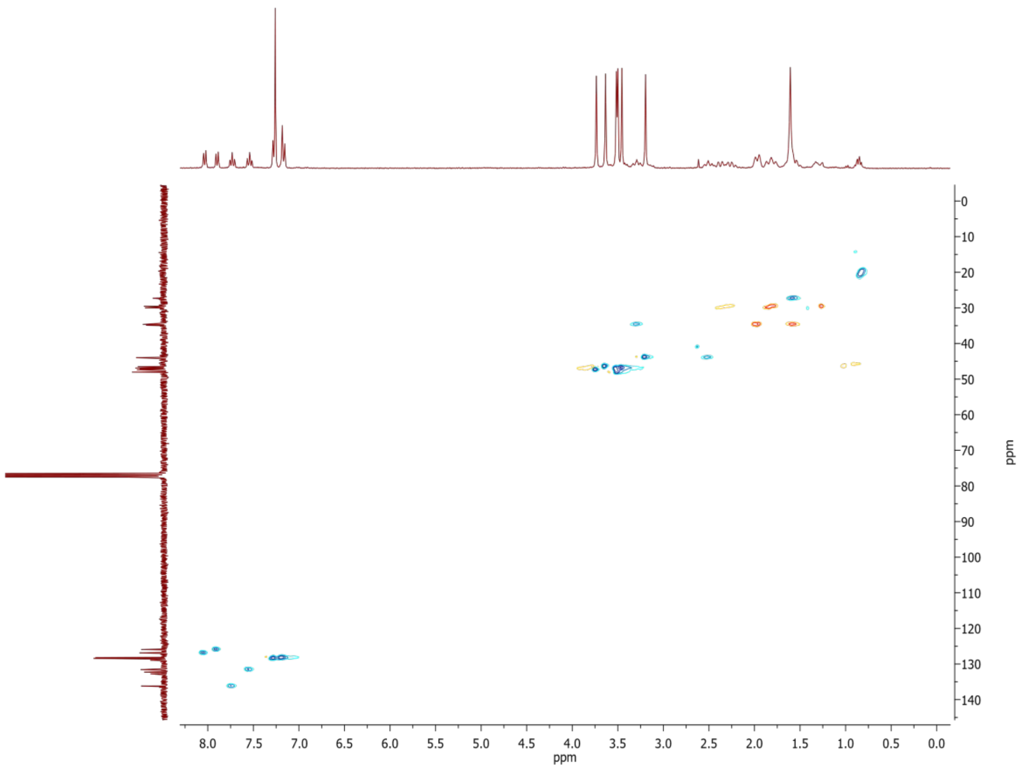

Figure S13. 2D NMR ^1H-^{13}C HMBC correlation contour map obtained for complex (**3**) ($CDCl_3$).

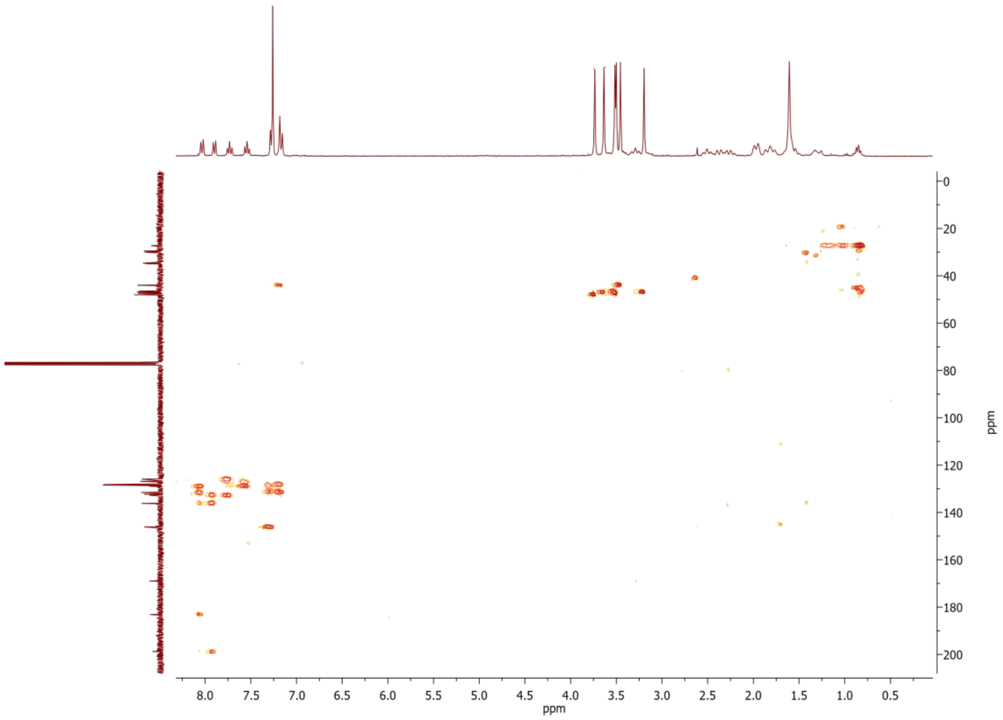

Figure S14. 1D ^{1}H NMR spectrum of complex (**2**) and the free atovaquone ligand ($CDCl_3$).

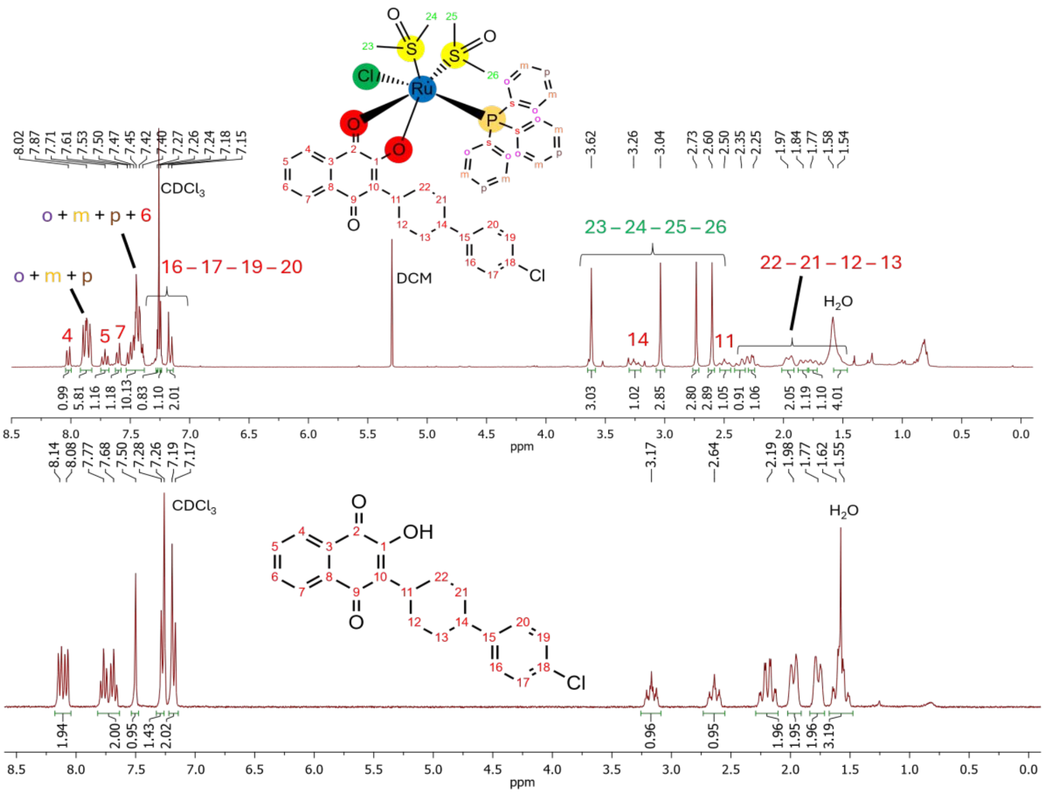

Figure S15. 1D ^{13}C NMR spectrum of complex (**2**) ($CDCl_3$).

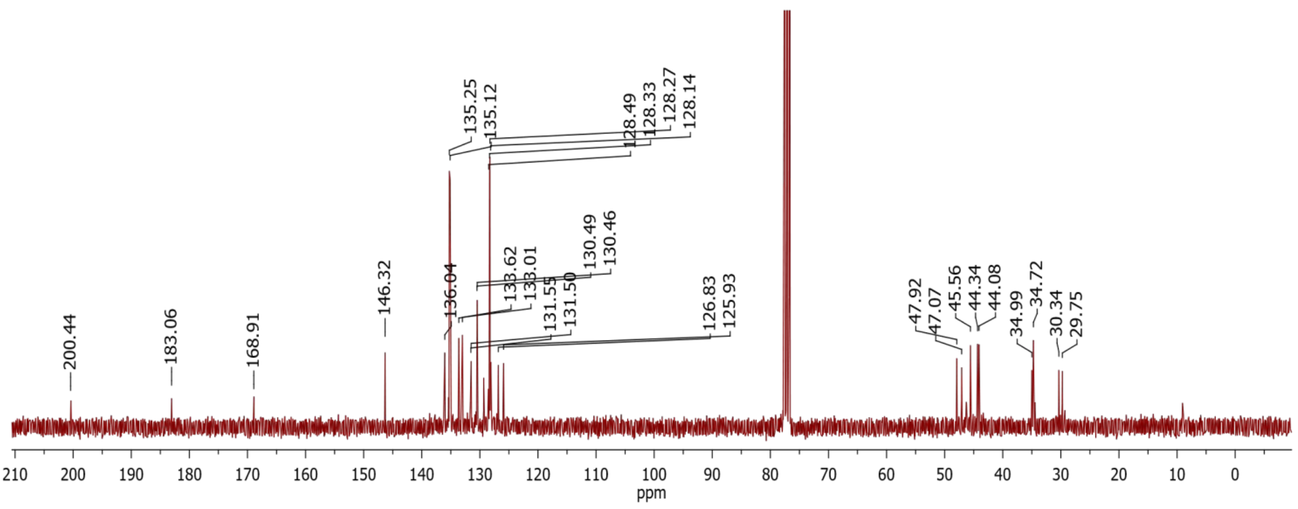

Figure S16. 2D NMR ^{1}H-^{1}H COSY contour map obtained for complex (**2**) ($CDCl_3$).

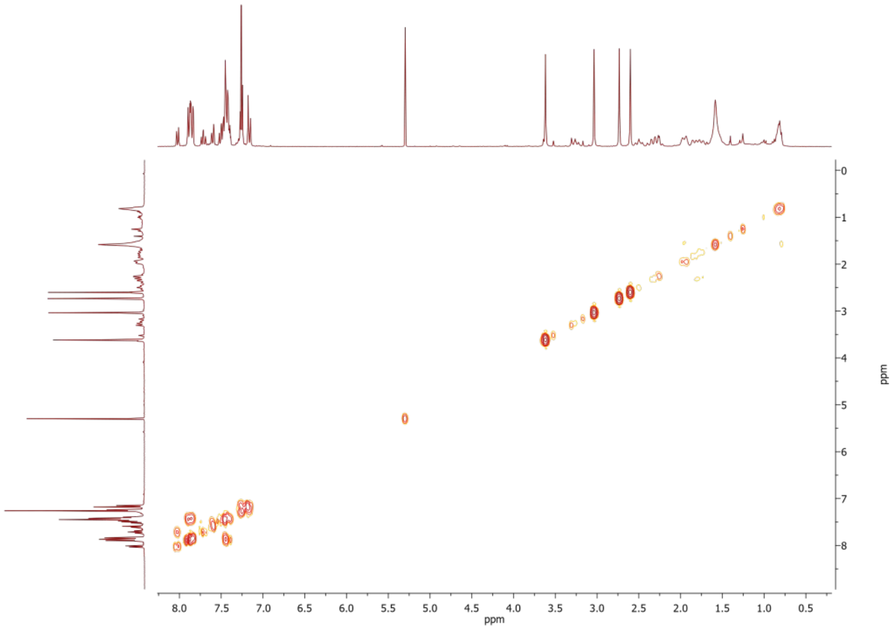

Figure S17. 2D NMR ^{1}H-^{13}C HSQC correlation contour map obtained for complex (**2**) (red: CH/CH_3; blue: CH_2; $CDCl_3$).

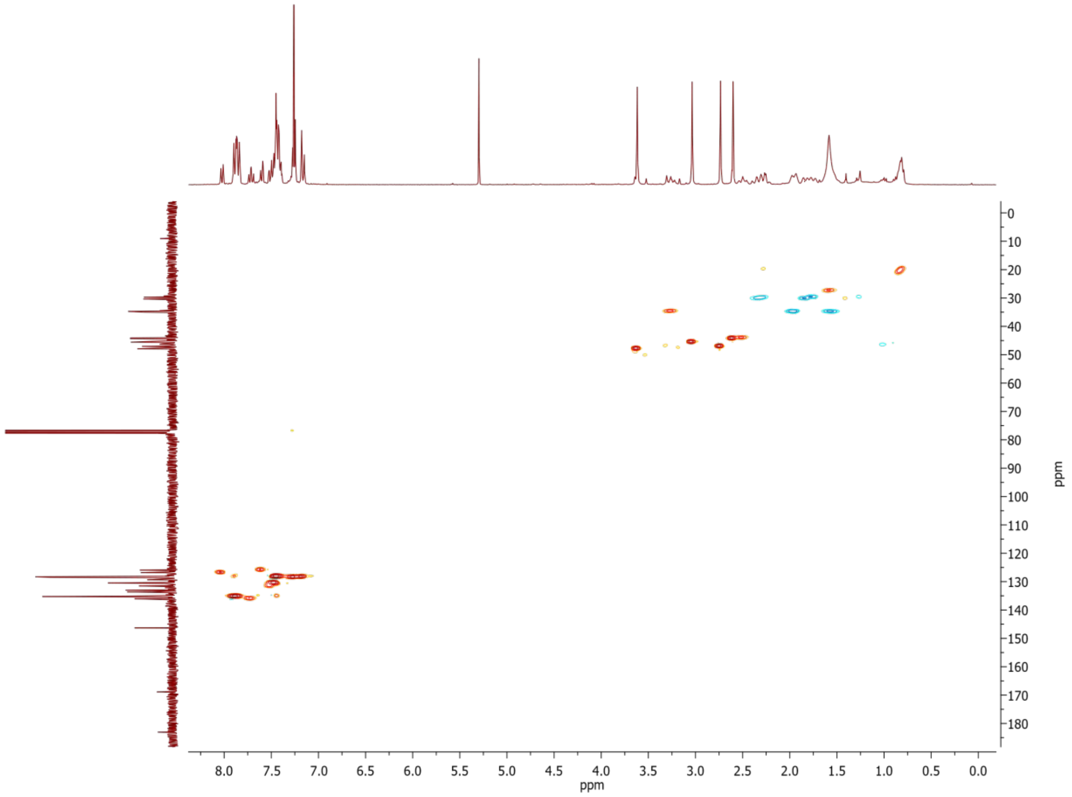

Figure S18. 2D NMR ^{1}H-^{13}C HMBC correlation contour map obtained for complex (**2**) ($CDCl_3$).

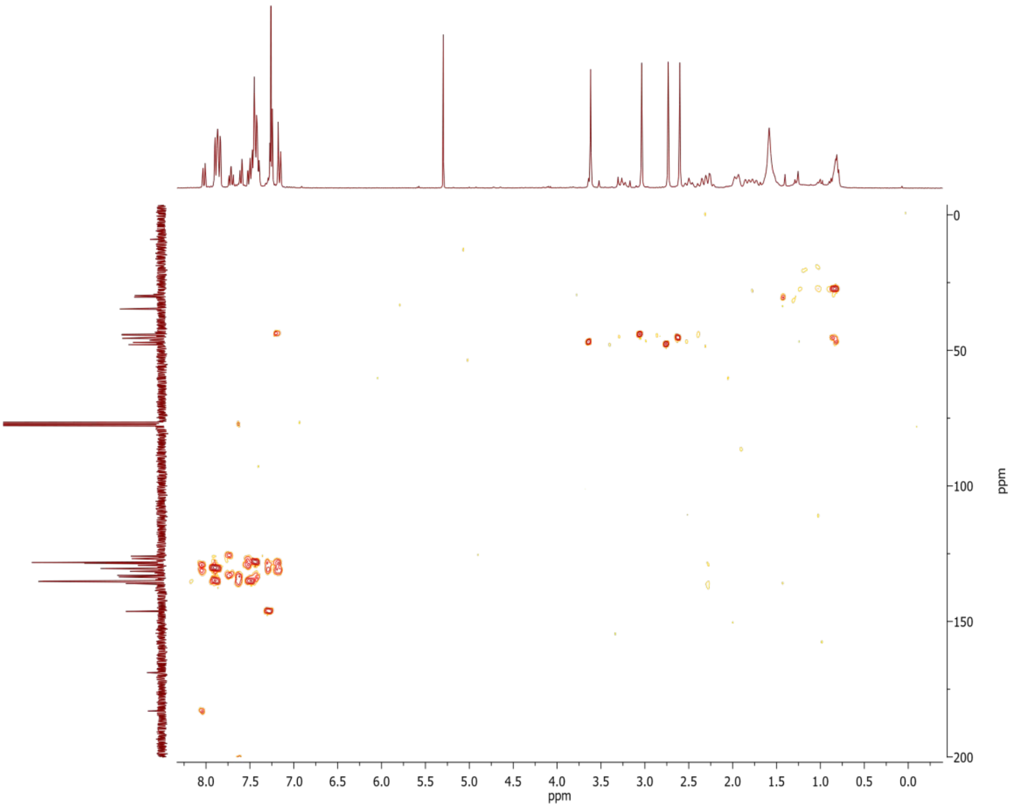

Figure S19. $^{31}P\{^{1}H\}$ NMR spectrum of complex (**2**) in dichloromethane with D_2O capillary.

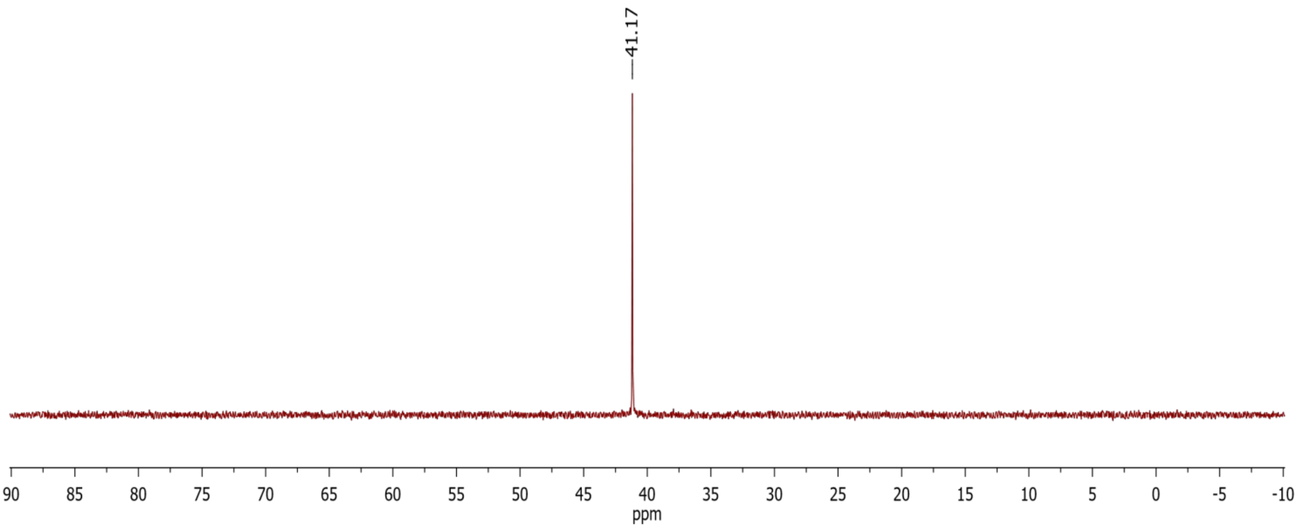

Figure S20. Cyclic and differencial pulse voltamogramms of the free ligand ATV in dichloromethane, PTBA 0.1 M, Pt WE/CE and Ag/AgCl reference electrode.

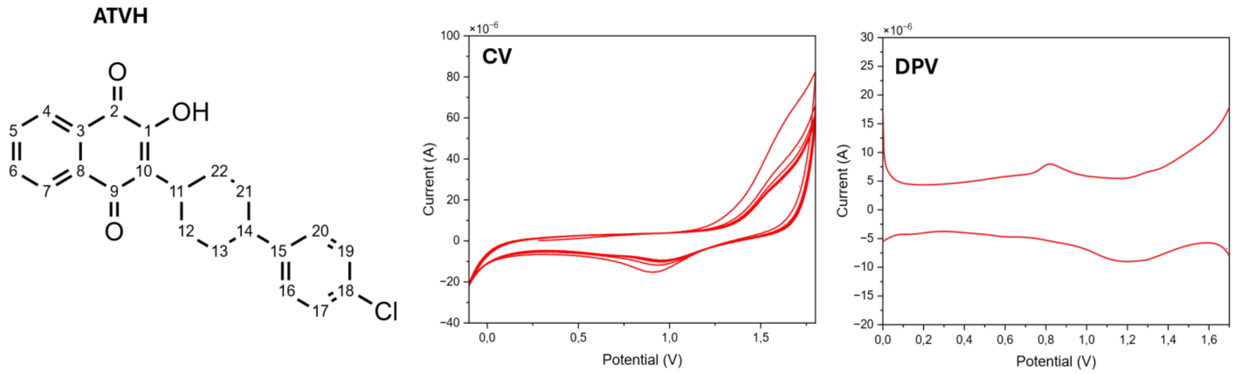

Figure S21. Cyclic and differencial pulse voltamogramms of complex (**1**) in dichloromethane, PTBA 0.1 M, Pt WE/CE and Ag/AgCl reference electrode.

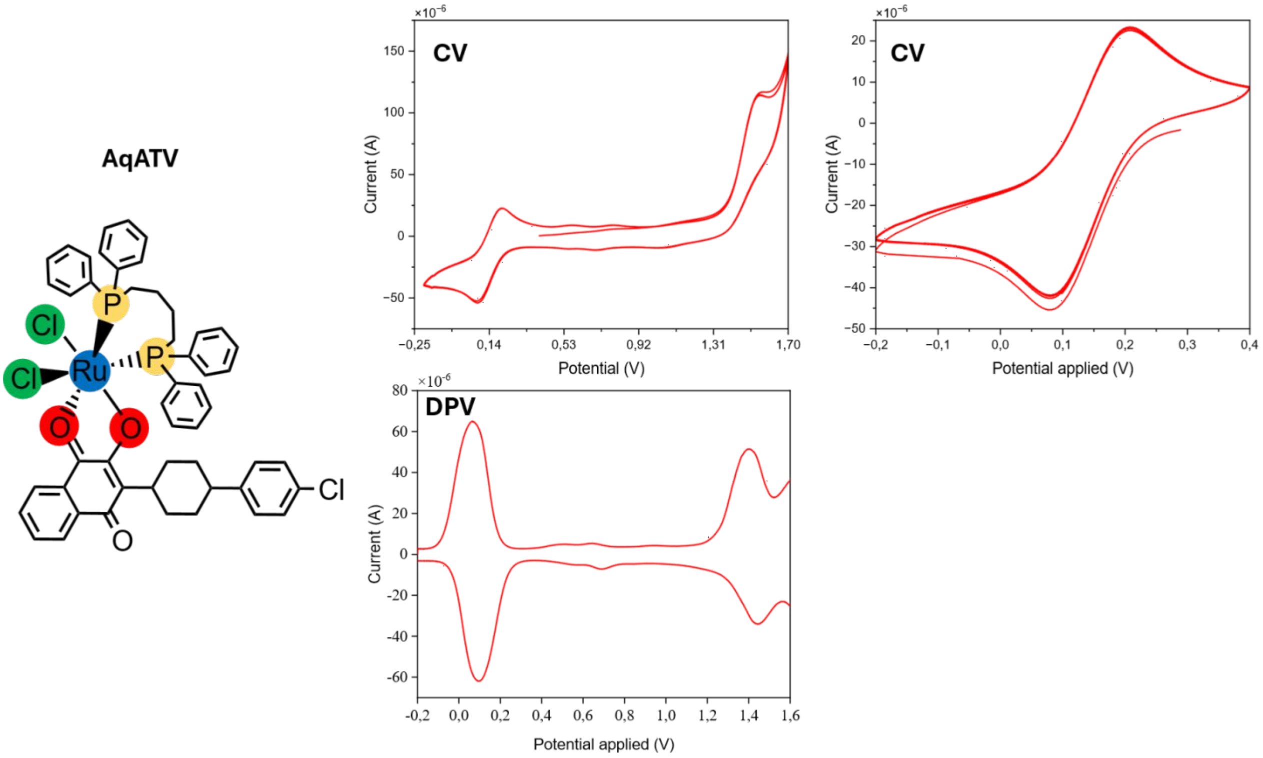

Figure S22. Cyclic and differencial pulse voltamogramms of complex (**2**) in dichloromethane, PTBA 0.1 M, Pt WE/CE and Ag/AgCl reference electrode.

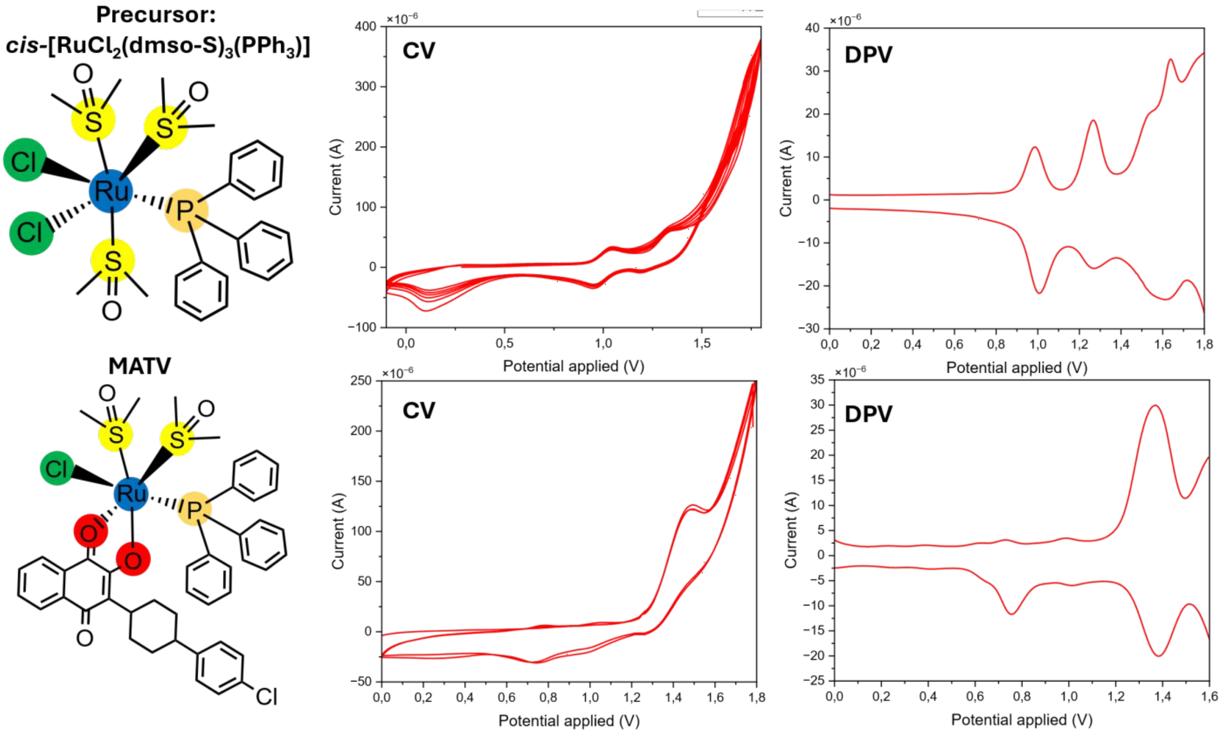

Figure S23. Cyclic and differencial pulse voltamogramms of complex (**3**) in dichloromethane, PTBA 0.1 M, Pt WE/CE and Ag/AgCl reference electrode.

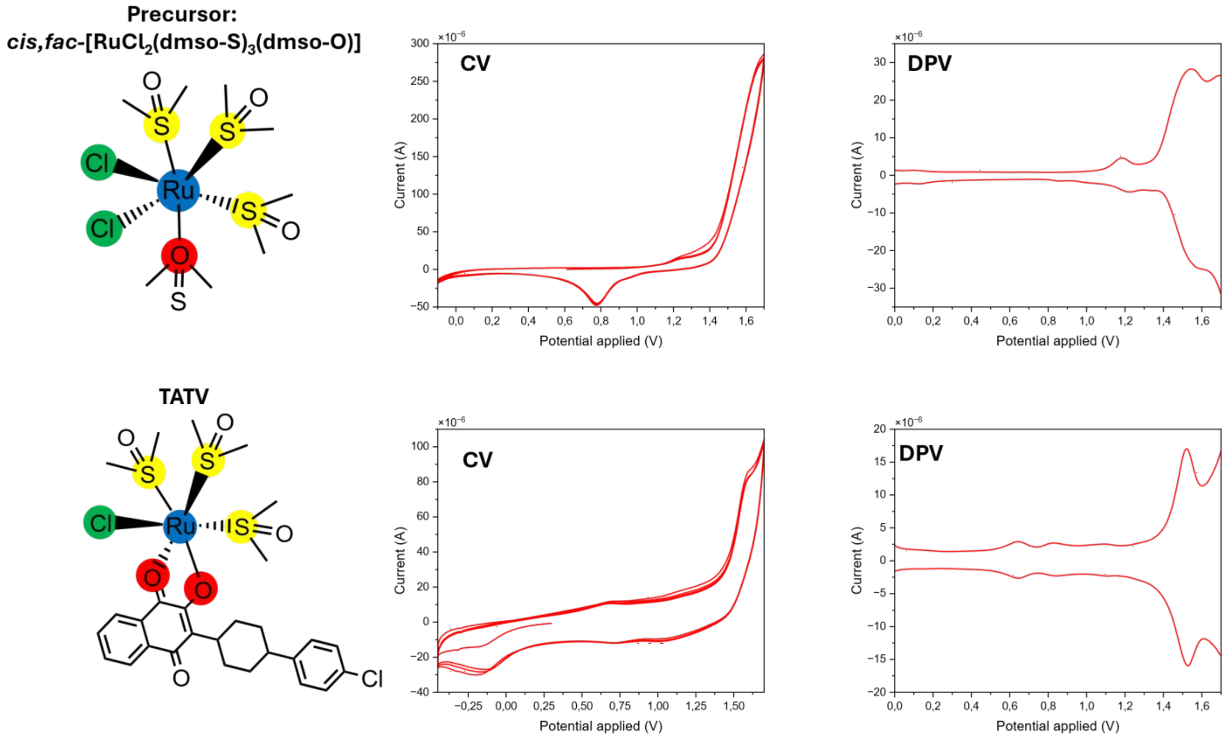

Figure S24. (a) Experimental electron paramagnetic resonance (EPR) spectra of complex (**1**) recorded at 77 K in the solid state and in CH_2Cl_2 solution. (b) Deconvolution and simulation of the EPR spectra in solution, showing the contribution of two components to the overall signal.

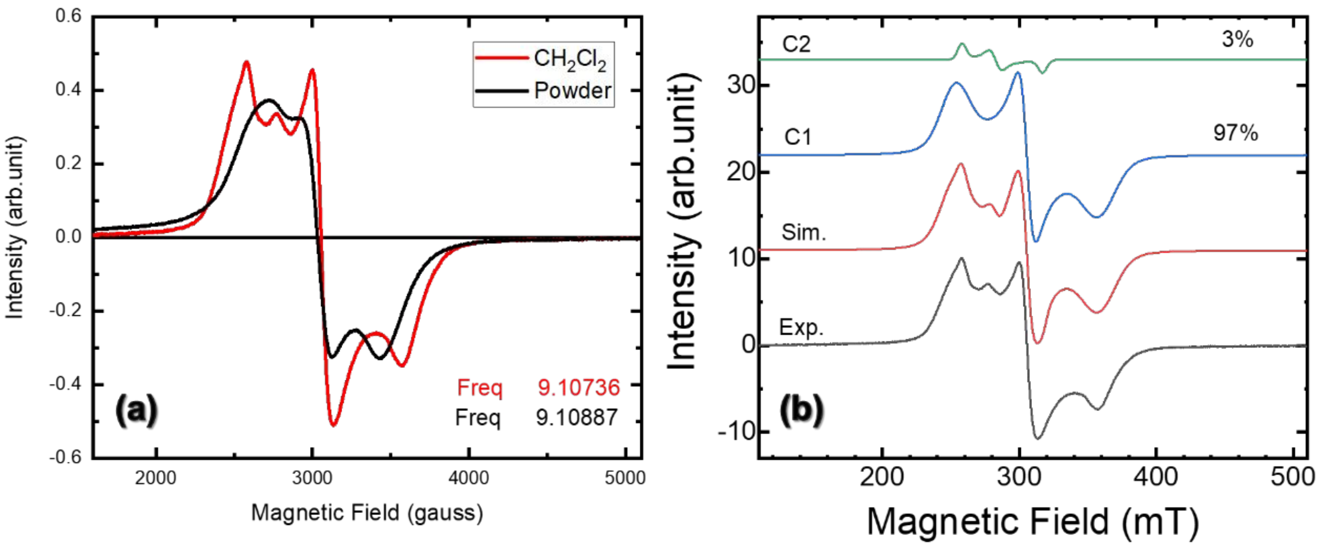

Figure S25. Hirshfeld surfaces of complexes (**1-3**) mapped with d_{norm} function and the respective atom types responsible for the most relevant contacts (top). Quantitative two-dimensional FingerPrint Plots (middle) and the graph of the percentage fractions (bottom) of individual atom contacts present on the Hirshfeld surface of the complexes. The color of the regions marked on the FP plots match the color of the bars in respect to the following reciprocal contacts: H···H in dark blue; H···C in red; H···Cl in green; C···C in orange; H···O in yellow; Cl···O in pink and others and black.

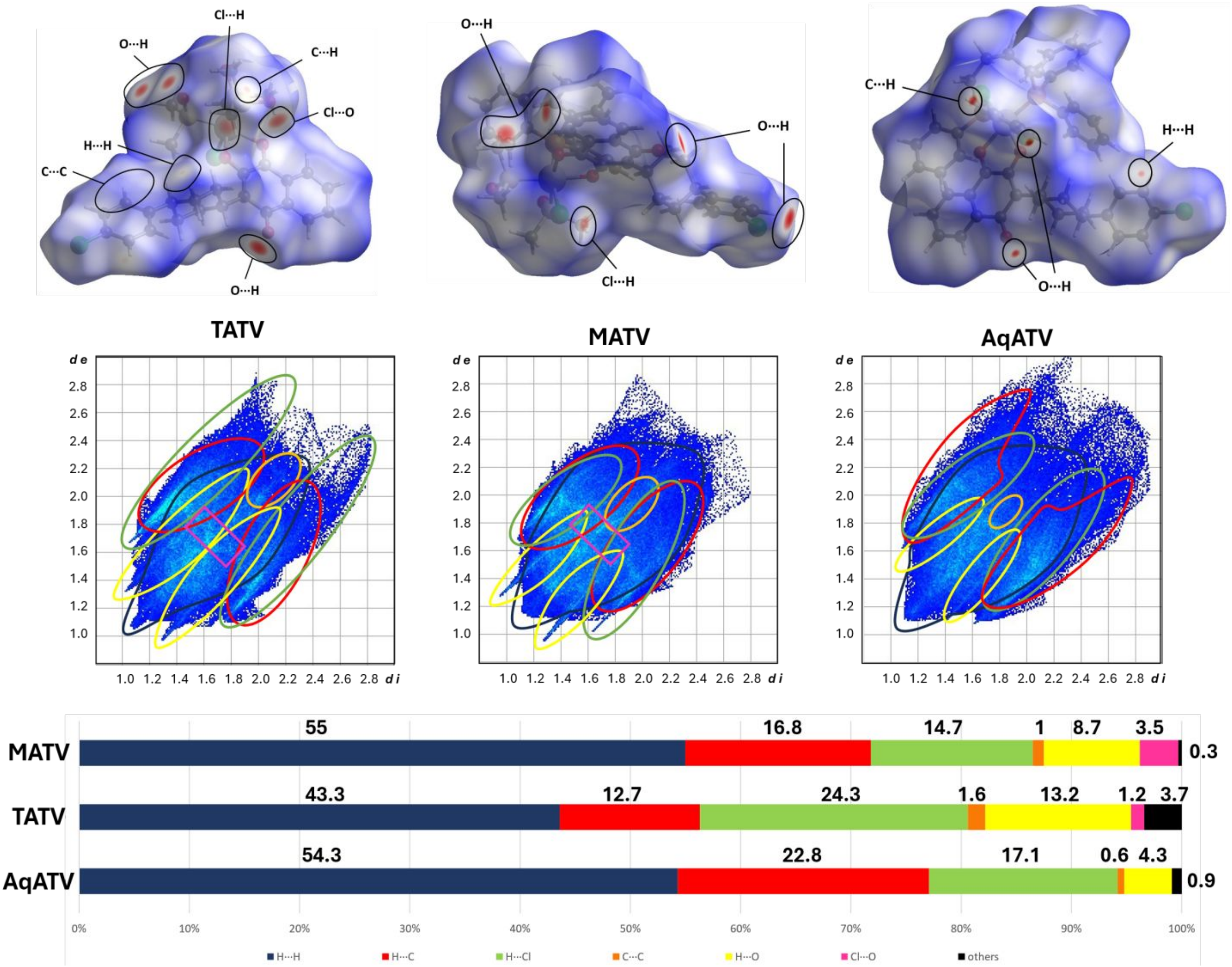

Figure S26. Full interaction maps for complexes (**3**, panel A); (**2**, panel B) and (**1**, panel C). The calculated clouds surrounding the complexes' environment are color coded: uncharged NH nitrogen probe in blue; RNH_3 nitrogen probe in purple; carbonyl oxygen probe in red; aromatic CH carbon probe in yellow.

(a)

(b)

(c)

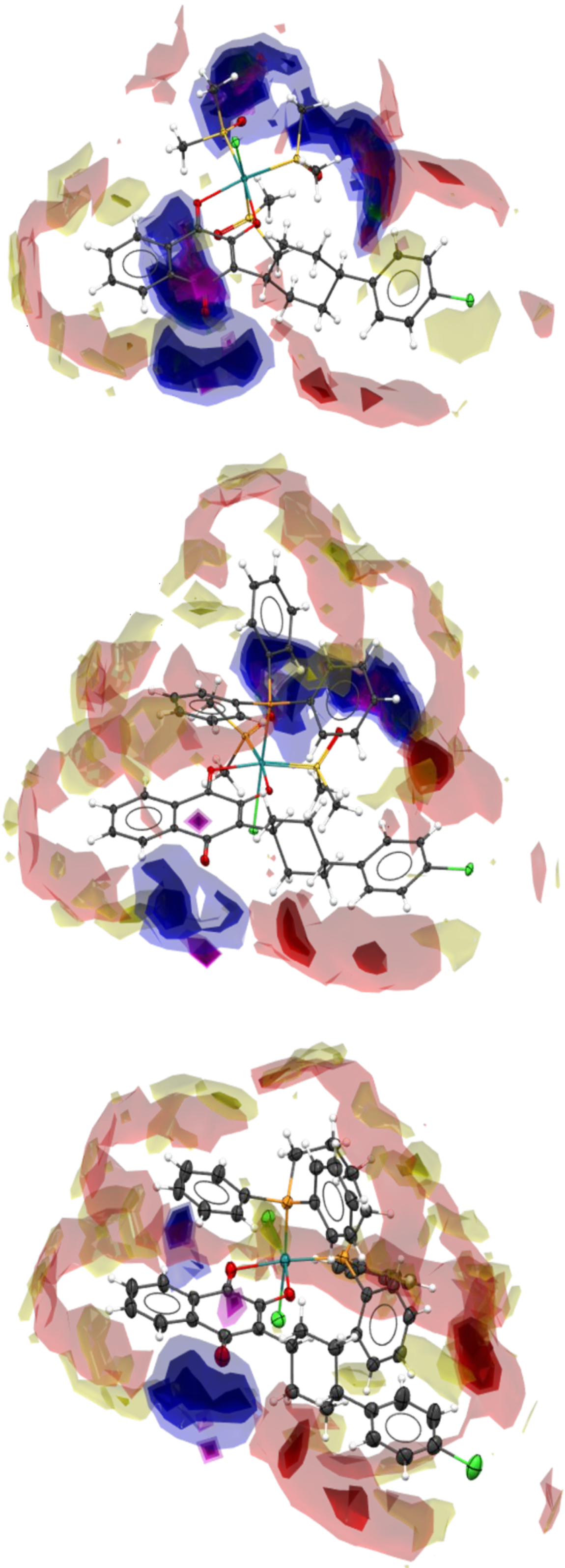

Figure S27. Torsion angles of the atovaquone's chlorophenyl ring (green) in respect to the naphthoquinone rings (red) for complexes (**1-3**). Hydrogen atoms were omitted for clarity.

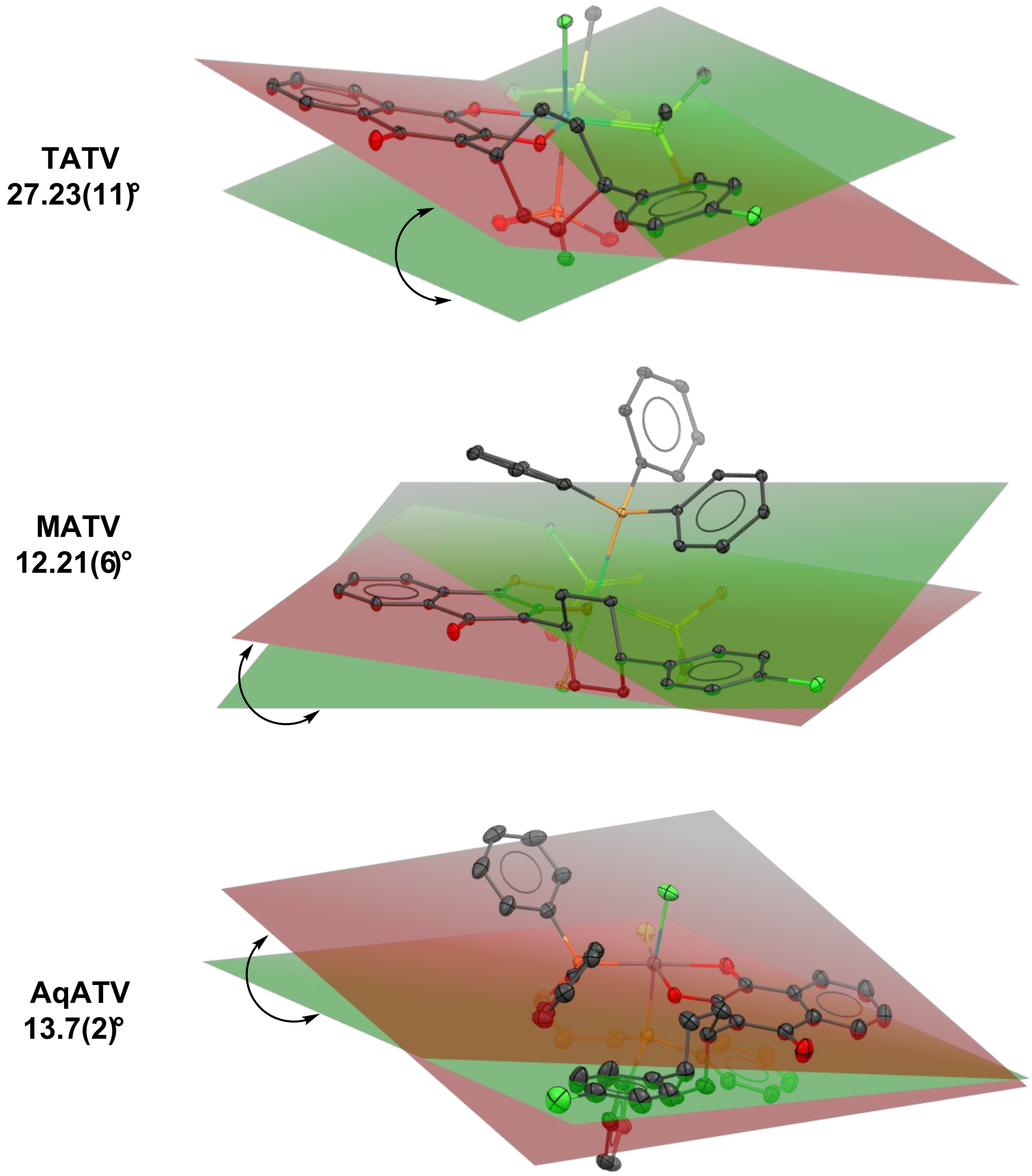

Figure S28. Stability study by Uv-Vis absorption of complex (**3**) in DMSO.

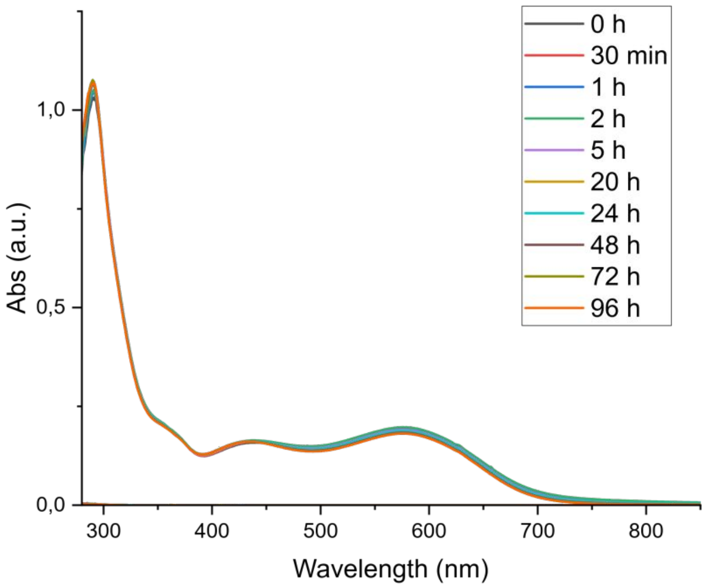

Figure S29. Stability study by Uv-Vis absorption of complex (**2**) in DMSO.

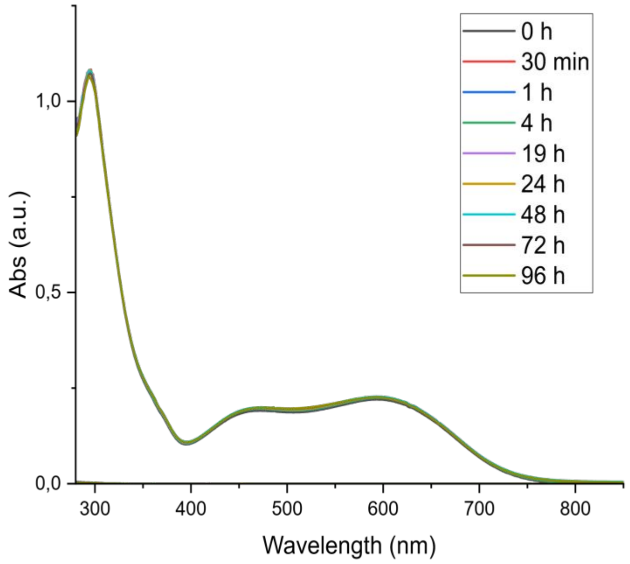

Figure S30. Stability study by Uv-Vis absorption of complex (**1**) in DMSO.

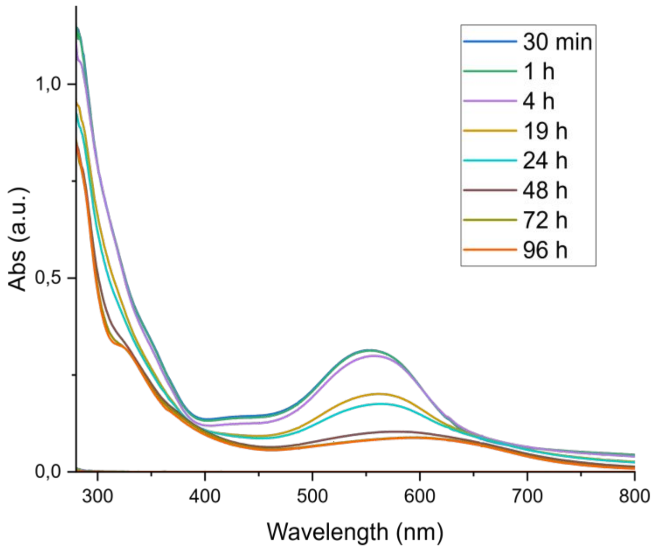

Figure S31. Stability study by Uv-Vis absorption of complex (**3**) in 50% DMSO/H_2O.

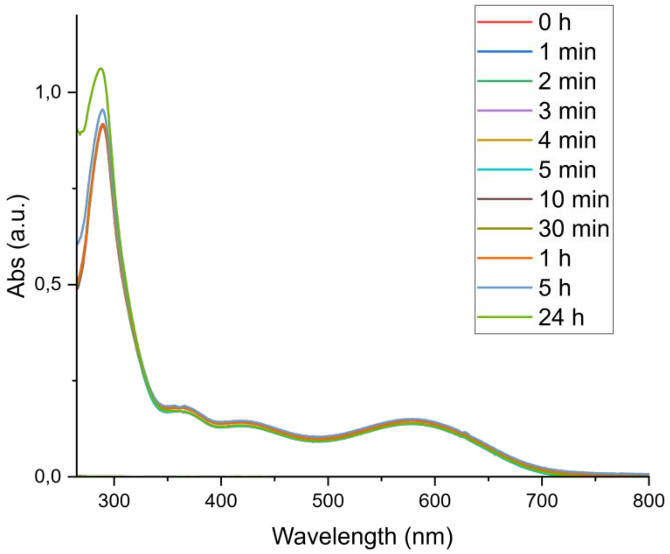

Figure S32. Stability study by Uv-Vis absorption of complex (**2**) in 50% DMSO/H_2O.

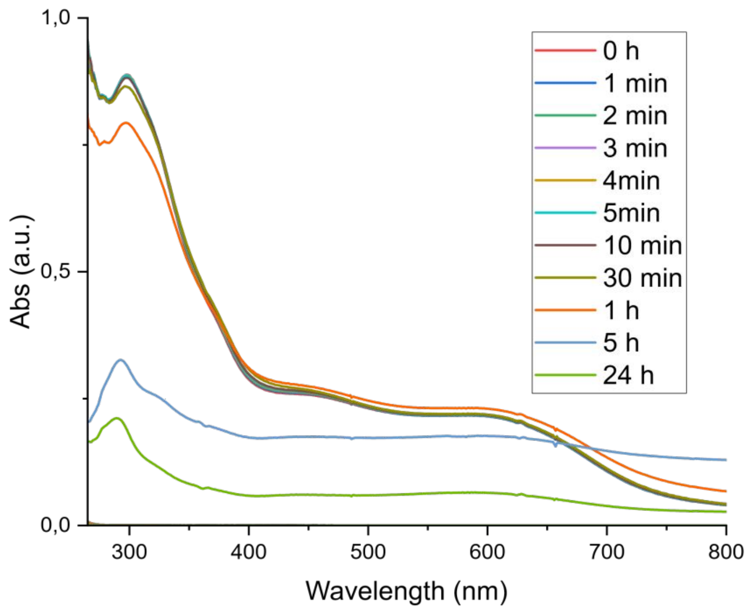

Figure S33. Stability study by Uv-Vis absorption of complex (**1**) in 50% DMSO/H_2O.

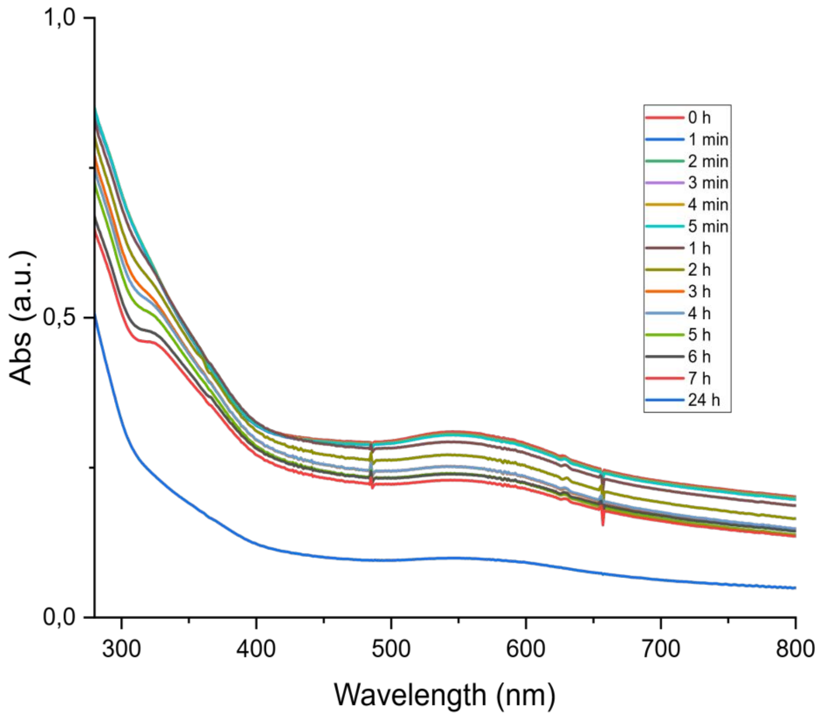

Figure S34: ^{1}H NMR spectra employed for monitoring the stability of complex (**2**) in solutions containing DMSO-d_6 and D_2O. The two lowest spectra display the chloride abstraction from the reaction upon adding $AgClO_4$. The initial chloride containing complex is depicted with a green star, and the speciated complexes acqua-**2** and dmso-**2** with a red circle and yellow heart, respectively.

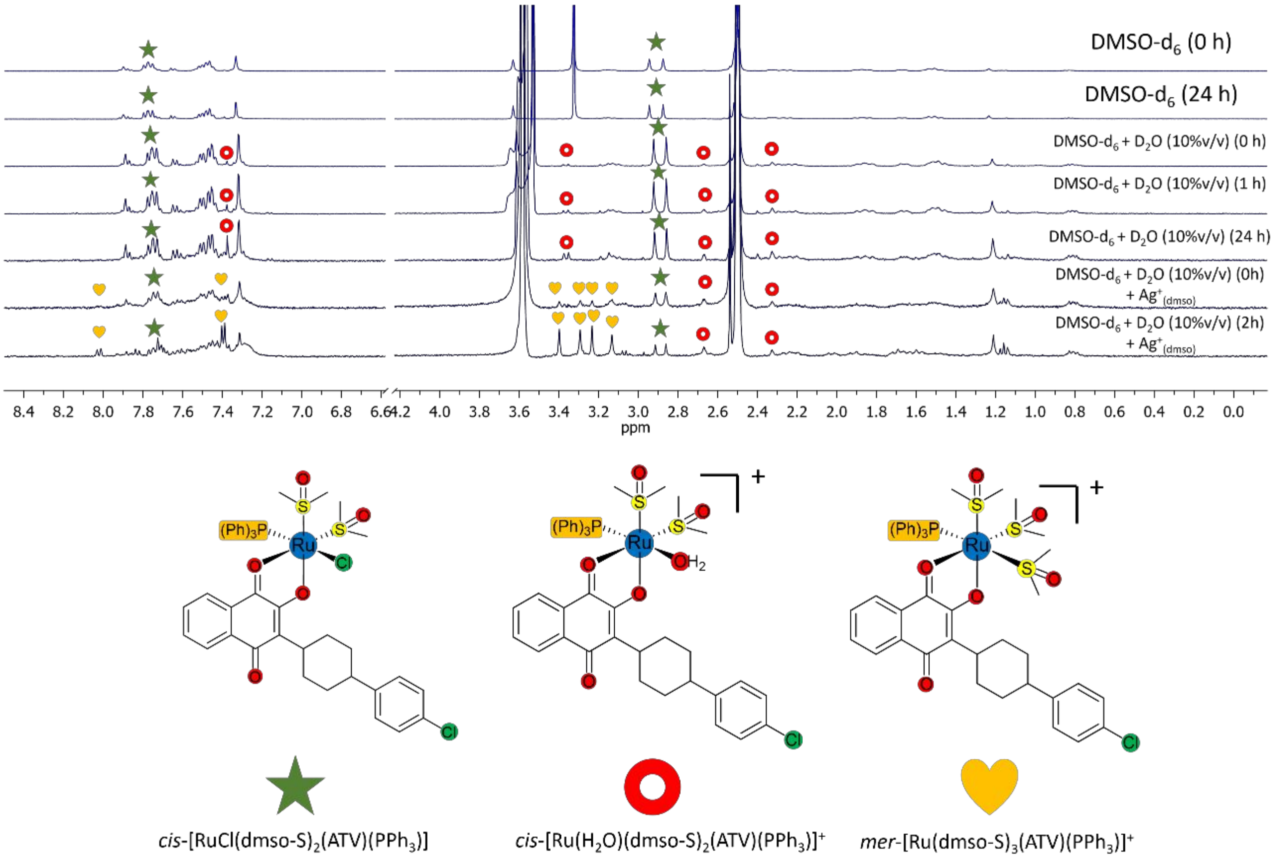

Figure S35: ^{1}H NMR spectra employed for monitoring the stability of complex (**3**) in solutions containing DMSO-d_6 and D_2O. The two lowest spectra display the chloride abstraction from the reaction upon adding $AgClO_4$. The initial chloride containing complex is depicted with a green star, and the speciated complexes acqua-**3** and dmso-**3** with a red circle and yellow heart, respectively.

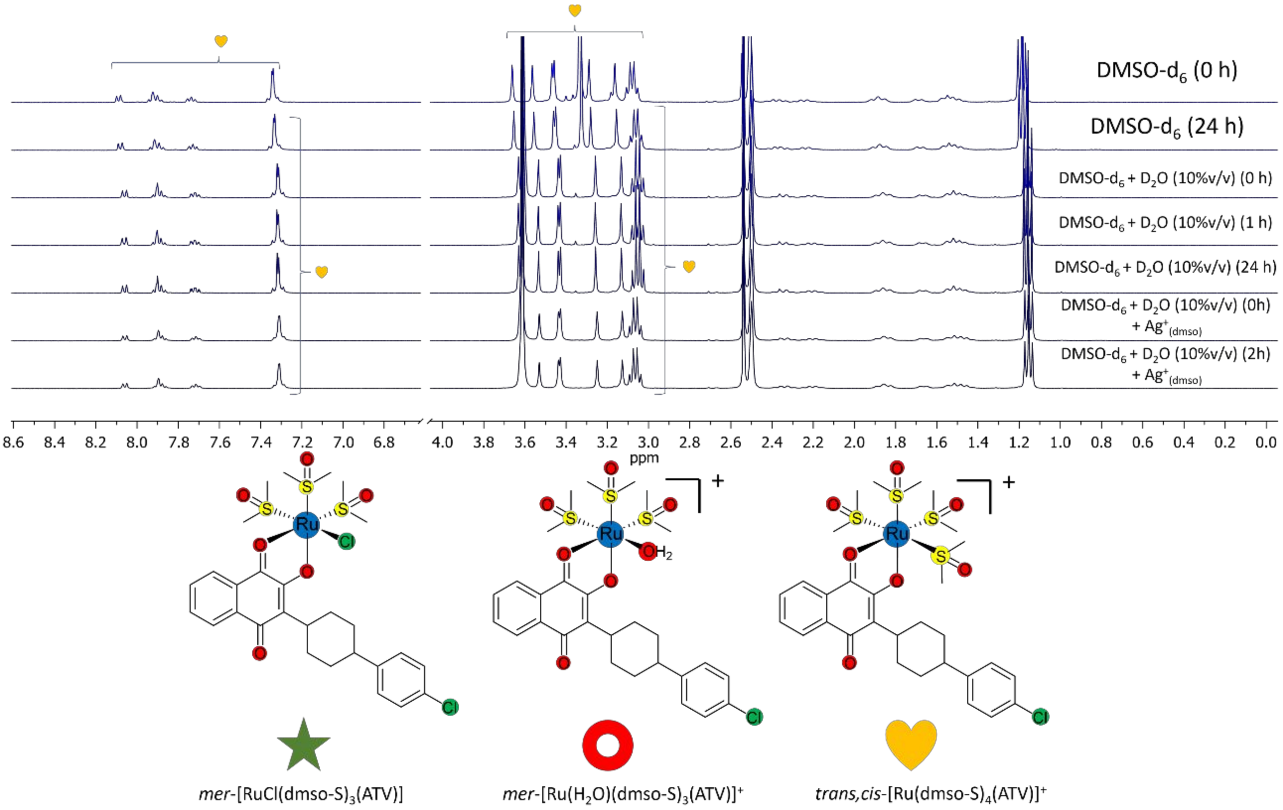

Figure S36: Fluorescence spectra of HSA solution (2.5 µM) in Tris–HCl buffer (0.1 M NaCl, pH 7.4) in the absence and presence of different concentrations of complexes. Panel A, complex (**3**); panel B, complex (**2;**) panel C, complex (**1**). Conditions: a = 0; b= 2.5 µM; c = 5.0 µM; d = 7.5 µM; e = 10.0 µM; f = 12.5 µM; g = 15.0 µM; h = 17.5 µM and i = 20.0 µM at 298 K. Inset: Stern–Volmer plots for the quenching of HSA fluorescence by complexes, 298 and 310 K.

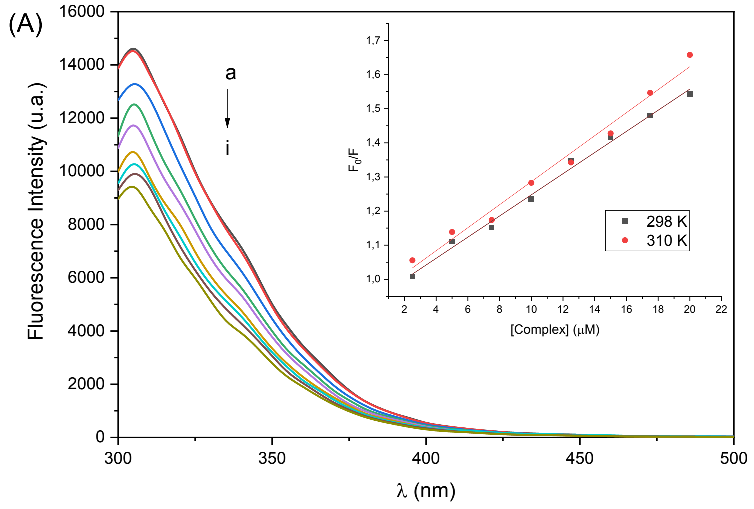

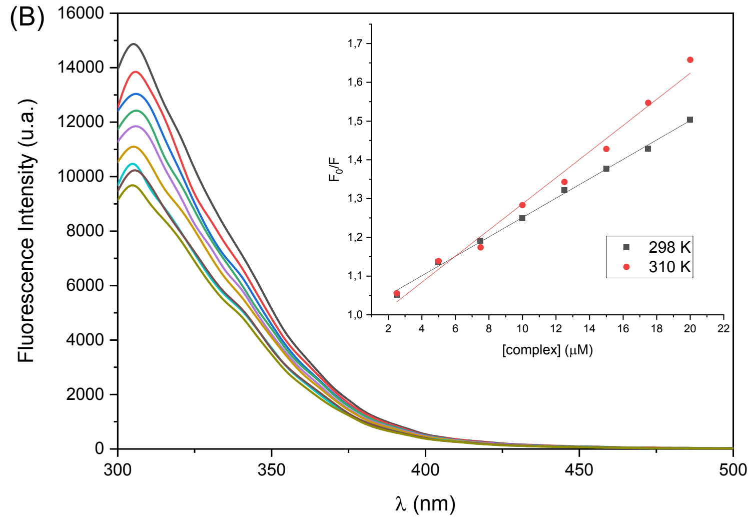

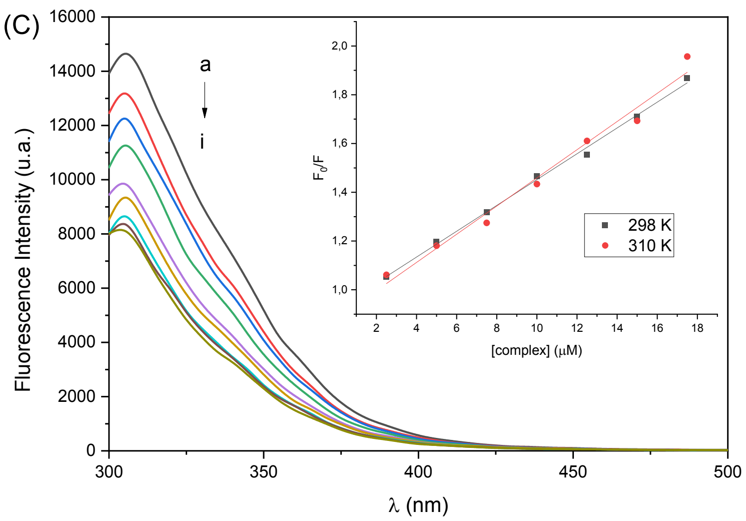

Table S3: Stern–Volmer quenching constant (K_{sv}, L mol^{-1}); biomolecular quenching rate constant (K_q, L mol^{-1} s^{-1}); binding constant (K_b, L mol^{-1}); number of binding sites (*n*), ΔG° (KJ mol^{-1}), ΔH° (KJ mol^{-1}) and ΔS° (J mol^{-1} K) values for the complex–HSA system at different temperatures.

Complexes	T (K)	K_{SV} (10^4)	K_q (10^{12})	K_b (10^4)	*n*	ΔH°	ΔS°	ΔG°
(3)	298	3.05 ± 0.04	4.92	3.93 ± 0.55	0.7	-19.7	22.0	-26.2
	310	3.28 ± 0.07	5.30	3.53 ± 0.16	0.8		23.7	-27.0
**(2)**	298	2.54 ± 0.16	4.11	2.63 ± 0.08	1.0	11.0	121.6	-25.2
	310	2.84 ± 0.38	4.58	2.95 ± 0.34	0.9		121.1	-26.5
**(1)**	298	4.51 ± 0.29	7.27	4.92 ± 0.25	0.8	16.1	143.8	-26.8
	310	4.33 ± 0.21	6.98	4.92 ± 0.47	0.8		141.7	-27.8

Table S4: Cell toxicity for HepG2 and J774 cells, association constant (log *K*) for hemin, β-hematin inhibitory activity (BHIA) and selectivity indexes (S.I) for Atovaquone and the metal complexes (**1-3**).

Compounds	HepG2 cells, CC_{50} ± S.E.M. [nM][a]	Log *K* (mean ± S.E.M.)[b]	BHIA, IC_{50} in mM (D:FePPIX ratio)[c]	J774 cells, CC_{50}±S.E.M. [nM][a]	S.I.[d]
Atovaquone, ATV	27800±800	4.21±0.2	> 2.0	29600±3300	20136
**(1)**	>80000	3.98±0.1	> 2.0	> 80000	155
**(2)**	17000±990	N.D.	> 2.0	4800±190	243
**(3)**	14600±2000	3.89±0.09	> 2.0	2800±200	241
[$RuCl_3$(dppb)]	>80000	N.D.	N.D.	> 80000	N.D.
Chloroquine, CQ	37600±3600	5.01±0.1	0.44±0.01 (1:4)	76100±3100	4909
Mefloquine, MQ	N.D.	4.51±0.1	1.79±0.02 (4:1)	N.D.	N.D.

[a] CC_{50} values in HepG2 hepatocarcinoma cells and J774 macrophage cell were determined after 72 h incubation and readout assessed by CellTitersGlo. Data are from three independent experiments using three technical

replicates. [b] Association constant to [Fe$^{(III)}$-PPIX] (hemin). Values are median±S.E.M. of three independent experiments. [c] β-hematin formation upon incubation with compounds and determined after 24 h. Values in parentheses correspond to drug:hematin equimolar ratio. Values are the mean and S.D. of two independent experiments using three technical replicates. [d] Selectivity indexes were determined in CC_{50} values for J774 cells versus IC50 values for asynchronous 3D7 strain. Abbreviations: S.D. = standard deviation. CQ = Chloroquine; S.E.M. = standard error of the median; MQ = mefloquine; ATV = atovaquone; N.D. = not determined.

Table S5: ATV and its Ru complexes (**2 and 3**) inhibit the growth of asexual blood stages of *P. falciparum*. Growth was assessed by SYBR green I method. Goodness-of-fit of non-linear model was assessed using the coefficient of determination (R^2). Values associated with Figure 3B in the main manuscript text.

**Compounds**	**3D7 strain of *P. falciparum*, IC_{50} [nM] (mean ± 95% CI) and the coefficient of determination (R^2)**		
	**24 h**	**48 h**	**72 h**
Dihydroartemisinin, DHA	1.7 (1.2-2.1)<br>R^2=0.9983	1.8 (1.6-2.0)<br>R^2= 0.9936	1.9 (1.6-2.1)<br>R^2=0.9916
Atovaquone, ATV	1.2 (N.D.-3.0)<br>R^2=0.8789	1.2 (1.2-1.3)<br>R^2=0.9873	0.57 (0.30-0.79)<br>R^2=0.9943
Ru(II) (**2**)	12.6 (0.25-31.1)<br>R^2=0.8786	9.8 (7.5-13.4)<br>R^2=0.9965	4.6 (3.7-8.1)<br>R^2=0.9636
Ru(II) (**3**)	3.3 (2.8-3.8)<br>R^2=0.9938	2.4 (2.1-2.7)<br>R^2=0.9957	1.0 (0.80-1.1)<br>R^2=0.9953

Table S6: Activity of metal complexes in inhibiting the growth of asexual blood stages of *P. falciparum*.

**Compounds**	**3D7 strain of *P. falciparum*, IC_{50} [nM] (mean ± 95% CI) and coefficient of determination (R^2)[a]**	
	**24 h**	**72 h**
Ru(II) (**3**)	3.3 (2.8-3.8)<br>R^2=0.9938	2.4 (2.1-2.7)<br>R^2=0.9957
	Precursor of complex (**1**) alone	
fac-[$RuCl_3(H_2O)$(dppb)]	N.D.	> 1000
	Precursor of complex (**1**) in combination to ATV (1:1)	
fac-[$RuCl_3(H_2O)$(dppb)] plus ATV	5.9 (5.1-7.9)<br>R^2=0.8819	1.1 (0.51-1.9)

[a] Growth was assessed by SYBR green I method. Goodness-of-fit of non-linear model was assessed using the coefficient of determination (R^2).

Table S7: Direct membrane feeding assay (DMFA) from drug-coated surfaces which were exposure to the tarsal area or drugs added into the bloodmeal of mosquitoes.

Groups	DMFA on *An. darlingi* (tarsal) [a]	
	Infection rate (mean)	Infection intensity (mean ± S.D.)
Control	83.82	36.12 ± 37.35
ATV, 200 μmol/m²	29.86	8.44 ± 17.03
(**3**), 200 μmol/m²	45	29.35 ± 34.88
(**3**), 20 μmol/m²	55.9	22.08 ± 27.04
(**3**), 2 μmol/m²	63.2	22.95 ± 33.49
	**DMFA on *An. aquasalis* (drugs on bloodmeal) (trial#1) [b]**	
**Groups**	**Infection rate (mean)**	**Infection intensity (mean ± S.D.)**
Control	58.9	13.80 ± 18.88
ATV, 10 μM	3.2	0.03 ± 0.19
Ru(II) (**3**), 10 μM	0.0	0.0 ± 0.0
Ru(II) (**3**), 0.5 μM	1.6	0.01 ± 0.13

[a] Thirty engorged mosquitoes were dissected per testing group and per patient. Each midgut was dissected at 7 days post-infection to detect *P. vivax* oocysts and oocyst intensity was determined. Values are derived from a pool of five patients.

[b] Thirty engorged mosquitoes were dissected per testing group and per patient. Each midgut was dissected at 7 days post-infection to detect *P. vivax* oocysts and oocyst intensity was determined. Values are derived from a pool of three patients.

Abbreviations: DMFA, direct membrane feeding assay; ATV, atovaquone.

Table S8: Direct membrane feeding assay (DMFA) from drugs added into the bloodmeal of *An. aquasalis*.

Groups	DMFA on *An. aquasalis* (drugs on bloodmeal) (trial#2)[a]	
	Infection rate (mean)	Infection intensity (mean ± S.D.)
Control	76.7	8.04 ± 8.66
ATV, 0.5 μM	5.5	0.09 ± 0.44
Ru(II) (**3**), 0.5 μM	4.4	0.05 ± 0.27
Ru(II) (**3**), 0.1 μM	11.3	0.29± 1.26

[a] Thirty engorged mosquitoes were dissected per testing group and per patient. Each midgut was dissected at 7 days post-infection to detect *P. vivax* oocysts and oocyst intensity was determined. Values are derived from a pool of three patients.

Abbreviations: DMFA, direct membrane feeding assay; ATV, atovaquone.

Figure S37: Panel A) Quantification of parasite stages in 3D7 strain of *P. falciparum* at 24 h. At least five fields from each slide were counted from giemsa staining. Values are the median. Panel B) Quantification of parasites (iRBCs) with visible hemozoin crystals determined by polarized light microscopy in 3D7 strain of *P. falciparum* at 48 or 72 h. Eight micrographs from each group were counted. Values are the mean and S.D. Data associated with Figure 4. Abbreviations: CTL, control; DHA, dihydroartemisinin; ATV, atovaquone; trophs, trophozoites.

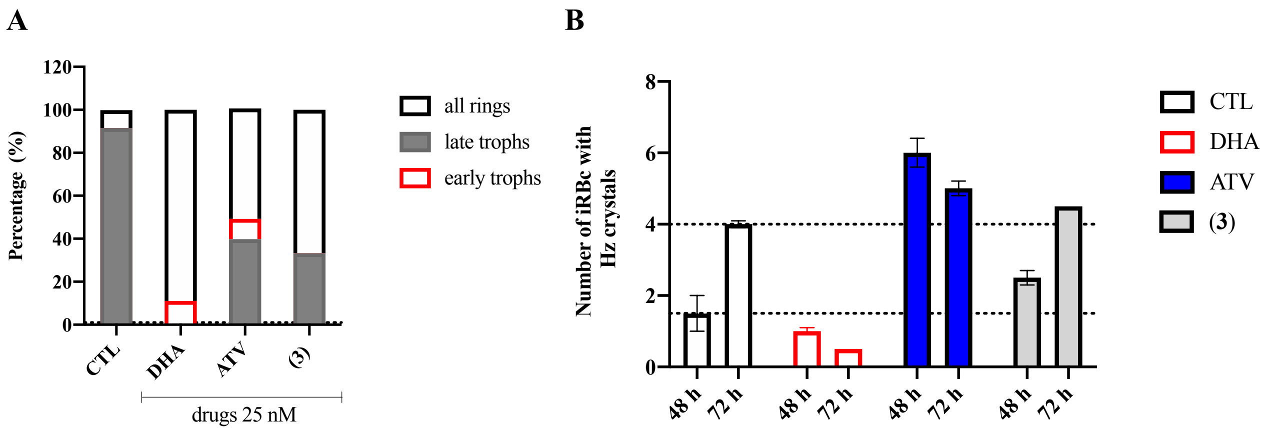

Figure S38. Survival of *An. darlingi* from drug-coated surfaces exposure to the tarsal area. Black lines show control groups, where mosquitoes were solely exposed with compound diluent. Panels A-D are from one experiment, while panels E-H are from another independent experiment. Panels A and E: Atovaquone (ATV) at 200 µmol/m^2; Panels B and F: complex (**3**) at 200 µmol/m^2; Panels C and G: complex (**3**) at 20 µmol/m^2; Panels D and H: complex (**3**) at 2 µmol/m^2. Indicated *p* values were calculated using log-rank (Mantel-Cox test).

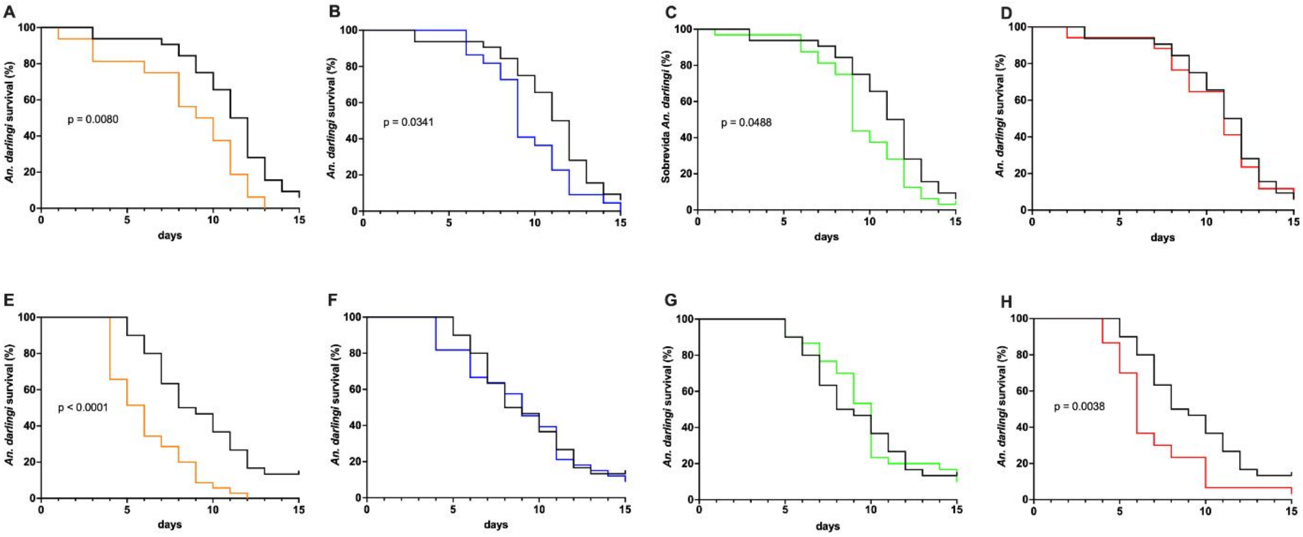

References

- Pavia, D. L., Lampman, G. M., Kriz, G. S., & Vyvyan, J. R. *Introduction to spectroscopy*. Cengage Learning, 2015.
- Oliveira, K. M.; Honorato, J.; Demidoff, F. C.; Schultz, M. S.; Netto, C. D.; Cominetti, M. R.; Correa, R. S.; Batista, A. A. Lapachol in the Design of a New Ruthenium(II)-Diphosphine Complex as a Promising Anticancer Metallodrug. *J. Inorg. Biochem.* **2021**, *214* (July 2020). https://doi.org/10.1016/j.jinorgbio.2020.111289.
- Gaur, R.; Mishra, L. Synthesis and Characterization of Ru(II)-DMSO-Cl-Chalcone Complexes: DNA Binding, Nuclease, and Topoisomerase II Inhibitory Activity. *Inorg. Chem.* **2012**, *51* (5), 3059–3070. https://doi.org/10.1021/ic202440r.
- Nayak, S. K.; Mallik, S. B.; Kanaujia, S. P.; Sekar, K.; Ranganathan, K. R.; Ananthalakshmi, V.; Jeyaraman, G.; Saralaya, S. S.; Rao, K. S.; Shridhara, K.; Nagarajan, K.; Row, T. N. G. Crystal Structures and Binding Studies of Atovaquone and Its Derivatives with Cytochrome Bc1: A Molecular Basis for Drug Design. *CrystEngComm* **2013**, *15* (24), 4871. https://doi.org/10.1039/c3ce40336j.
- Prajapati, R.; Dubey, S. K.; Gaur, R.; Koiri, R. K.; Maurya, B. K.; Trigun, S. K.; Mishra, L. Structural Characterization and Cytotoxicity Studies of Ruthenium(II)-Dmso-Chloro Complexes of Chalcone and Flavone Derivatives. *Polyhedron* **2010**, *29* (3), 1055–1061. https://doi.org/10.1016/j.poly.2009.11.012.
- Baggish, A. L.; Hill, D. R. Antiparasitic Agent Atovaquone. *Antimicrob. Agents Chemoth.* **2002**, *46* (5), 1163–1173. https://doi.org/10.1128/AAC.46.5.1163.
